# Supplementary material for: Genome-scale sequencing and analysis of human, wolf, and bison DNA from 25,000-year-old sediment
Source: Curr Biol. 2021 Aug 23;31(16):3564–3574.e9. doi: 10.1016/j.cub.2021.06.023 (PMC8409484; doi:10.1016/j.cub.2021.06.023)
Supplement: Document S1. Figures S1–S6 [file mmc1.pdf]

Current Biology, Volume *31*

Supplemental Information

Genome-scale sequencing and analysis

# of human, wolf, and bison DNA

from 25,000-year-old sediment

Pere Gelabert, Susanna Sawyer, Anders Bergström, Ashot Margaryan, Thomas C. Collin, Tengiz Meshveliani, Anna Belfer-Cohen, David Lordkipanidze, Nino Jakeli, Zinovi Matskevich, Guy Bar-Oz, Daniel M. Fernandes, Olivia Cheronet, Kadir T. Özdoğan, Victoria Oberreiter, Robin N.M. Feeney, Mareike C. Stahlschmidt, Pontus Skoglund, and Ron Pinhasi

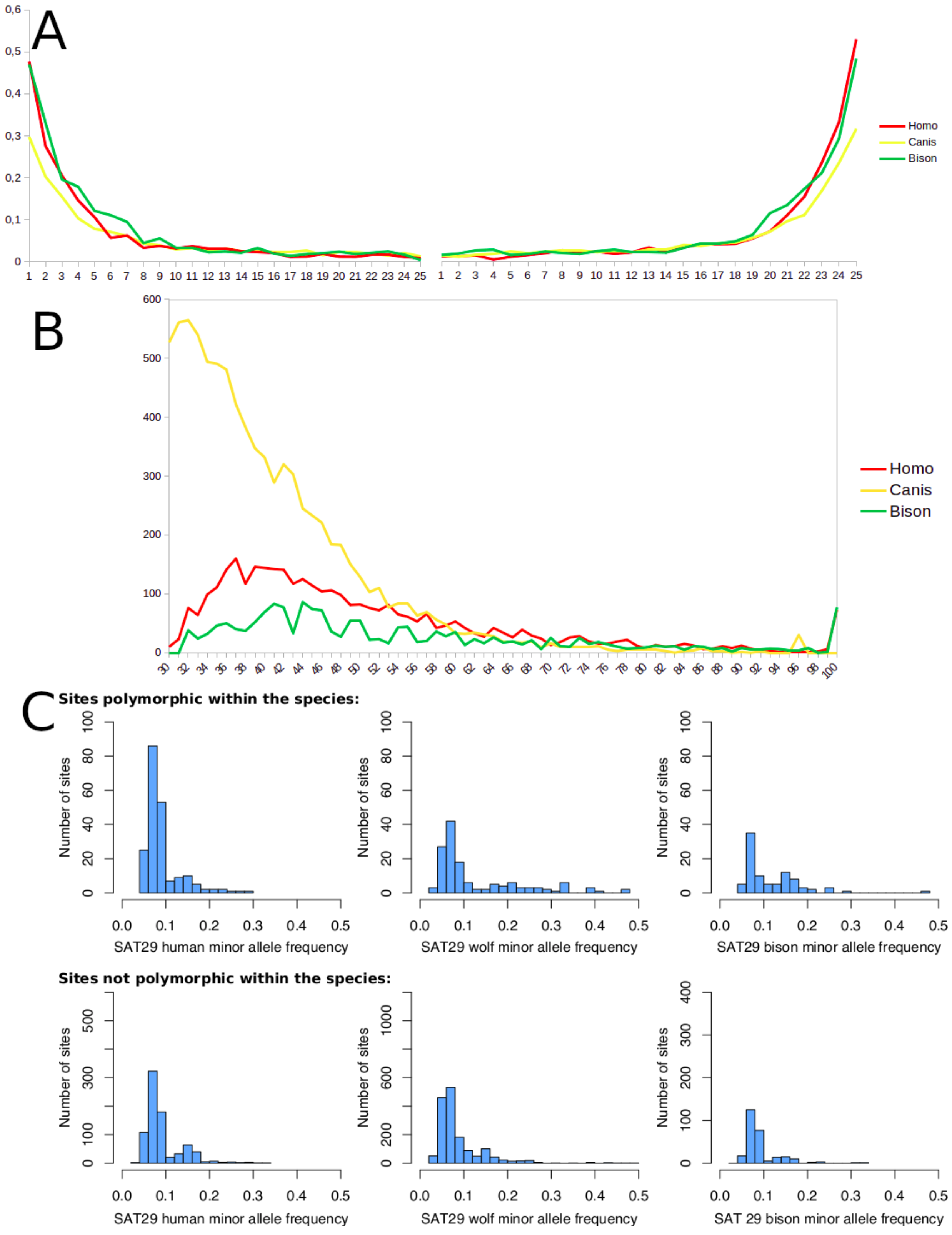
A
Homo
Canis
Bison
B
Homo
Canis
Bison
C
Sites polymorphic within the species:
Number of sites
SAT29 human minor allele frequency
SAT29 wolf minor allele frequency
SAT29 bison minor allele frequency
Sites not polymorphic within the species:
SAT29 human minor allele frequency
SAT29 wolf minor allele frequency
SAT 29 bison minor allele frequency

Figure S1: Mitochondrial captured reads, Related to Figure 2: A) Deamination patterns. B) read length distributions for the captured mitochondrial reads. C) Minor allele frequency distributions for the three captured mitochondrial genomes. Only positions covered by at least 12 reads are included, and counts of sites with a minor allele frequency of 0 are omitted. The top row shows sites that have been ascertained to be polymorphic within the given species, while the bottom row shows the rest of the sites. This is informative about the relative coverage of the two alleles at each position. Most of the values fall between 0 and 0.2, which likely reflect the confounding effects of ancient DNA damage (e.g. 1 or 2 damaged reads out of 10-15 total reads). However, particularly in the wolf data, there are also quite a few reads that show minor allele frequencies larger than 0.2, especially at the sites harbouring known polymorphisms, which likely reflects the presence of more than one haplotype in the sample.

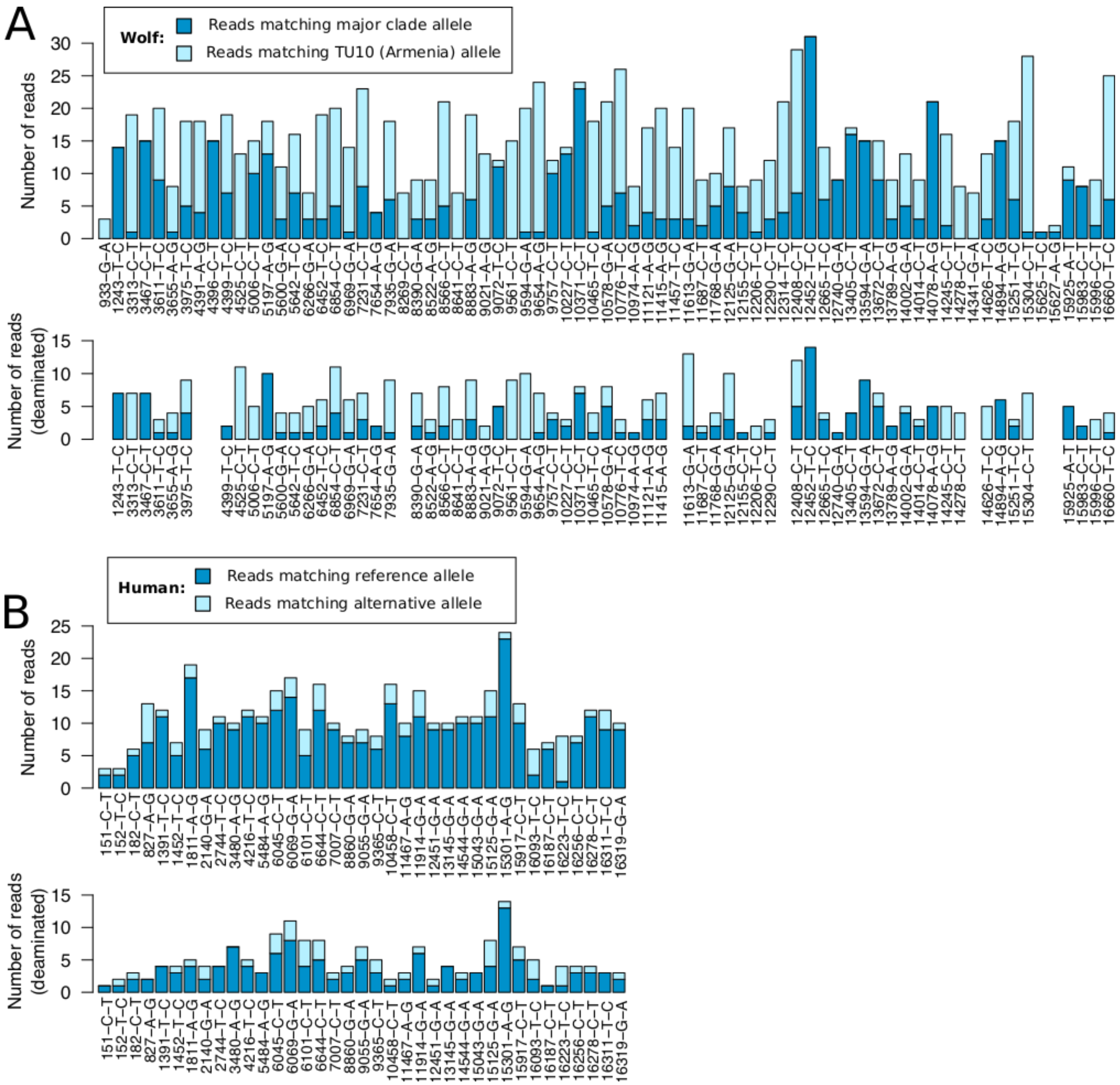

Figure S2: Evidence for polymorphism in the SAT29 wolf and human mitochondrial sequences, Related to Table 1: A) Sites that differ between the pre-LGM Armenian wolf TU10 and the major clade of wolf mitochondria were identified, and the read counts of the SAT29 sample at these sites displayed. The top row shows results using all reads, and the bottom row using only reads that display evidence of deamination. These results suggest that the retrieved DNA derives from more than one

wolf individual. B) Sites that display some evidence of possible polymorphism in the human SAT29 mitochondrial data were identified (Table S2), and the reads counts of the SAT29 sample at these sites displayed The top row shows results using all reads, and the bottom row using only reads that display evidence of deamination. The results suggest that much of the diversity signal in the human data is explained by damage, but some allele observations could also reflect genuine, low-level diversity in the sample.

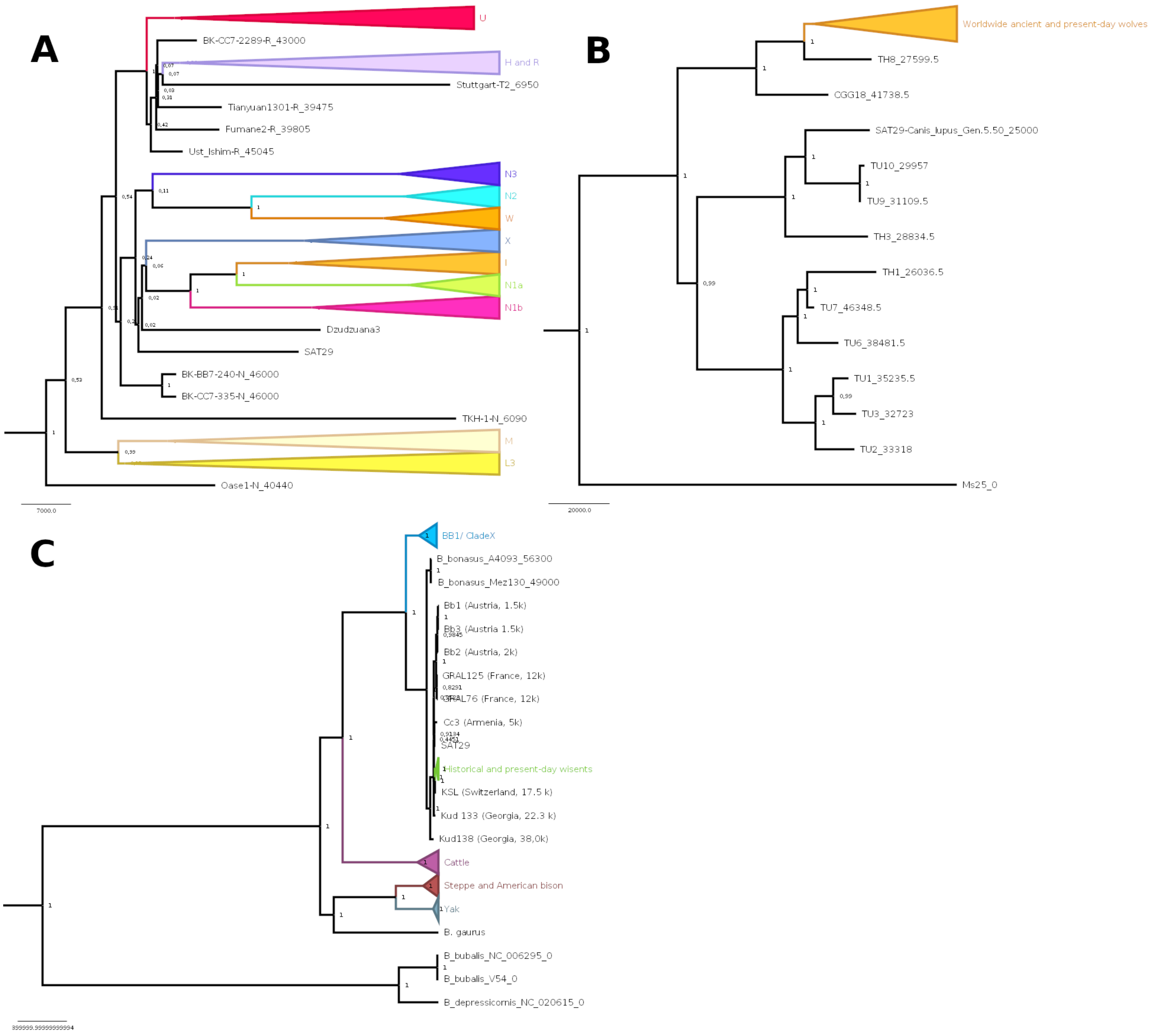

Figure S3, Related to Figures 3, 4 and 5: Maximum likelihood trees performed with the mitochondrial datasets. The node values represent the bootstrapping value. **A)** Homo, **B)** Canis and **C)** Bison.

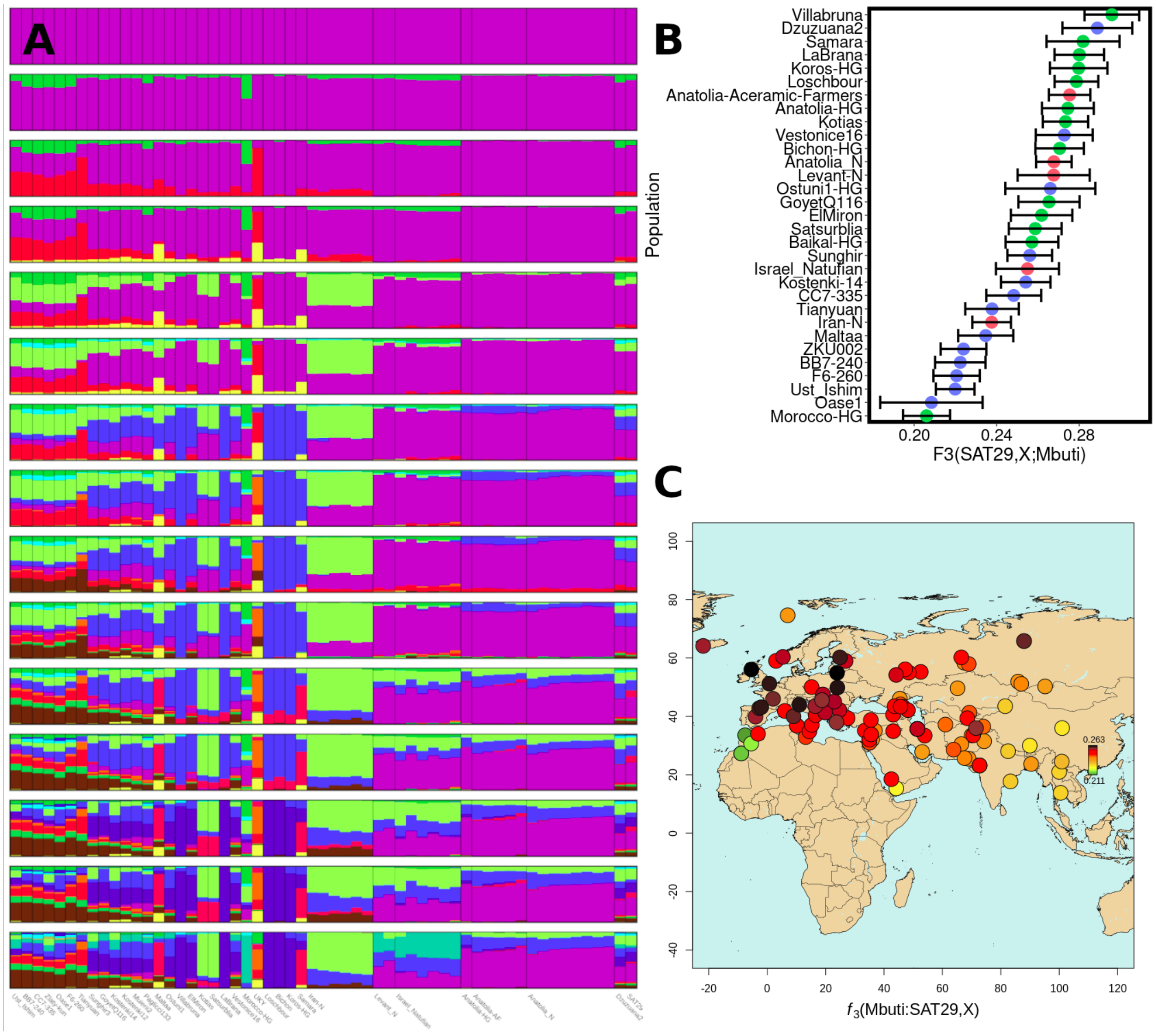

Figure S4: Human Population Genetics, Related to Figure 3: A) ADMIXTURE plots using K from 1 to 15 on the human dataset, **B)** Values of outgroup-f_3 statistics of the form f_3(SAT29,X;Mbuti). Only statistics with more than 2,000 SNPs were included. Green dots represent post-LGM samples, violet dots represent pre-LGM samples and red dots represent farmer samples. Error bars denote one standard error. **C**) Values of the statistic f_3 (SAT29,Modern-population;Mbuti), the intensity of color is proportional to the amount of shared drift.

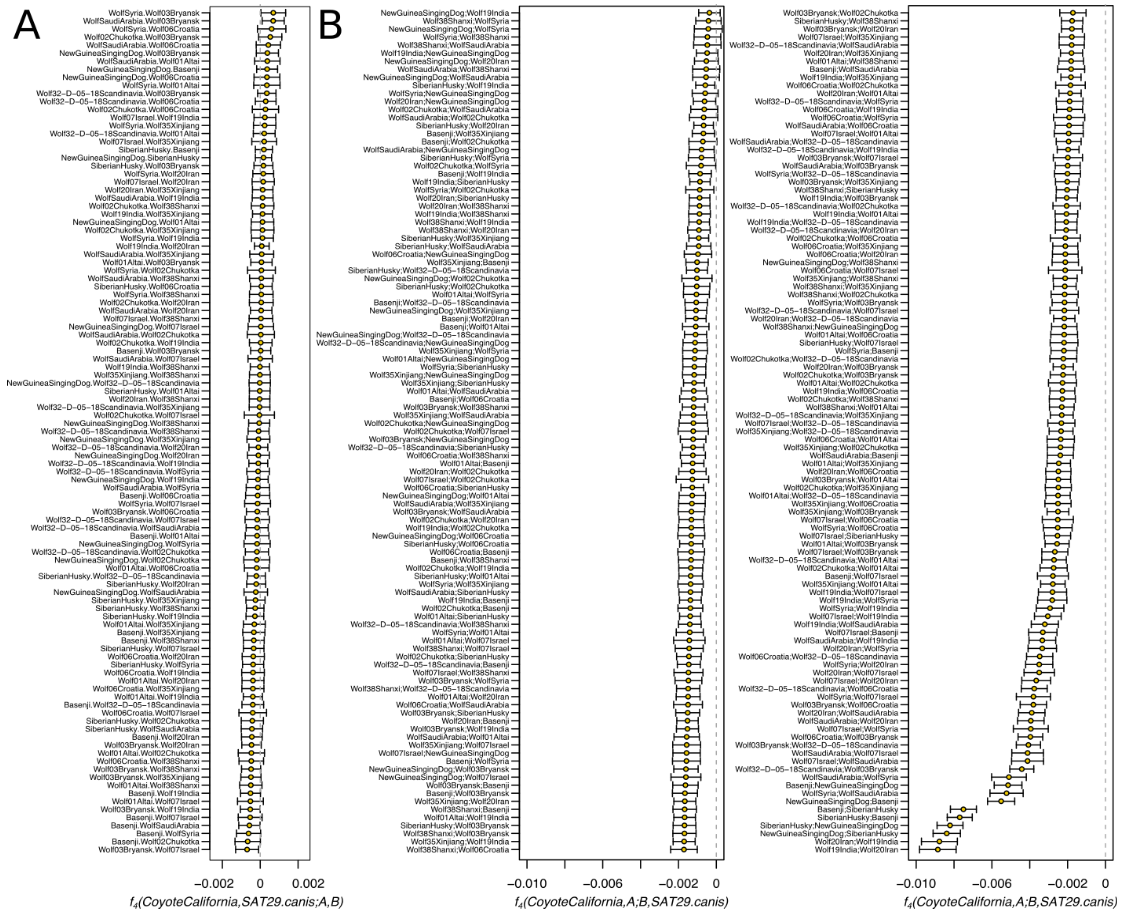

Figure S5: The SAT29 *Canis* genome is symmetrically related to modern wolves and dogs, and the latter share drift to the exclusion of SAT29, Related to Figure 4: f_4-statistics evaluating the basal ancestry status of the SAT29 canid genome in relation to a set of 15 diverse Eurasian present-day wolves and dogs. Error bars denote ±3 standard errors. A) Statistics of the form f_4(CoyoteCalifornia,SAT29.*Canis*;A,B) are consistent with being distributed around 0, indicating that SAT29 is symmetrically related to present-day individuals. A few statistics fall close to or slightly outside $|Z| > 3$ (largest $|Z| = 3.43$), and involve wolves from Southwestern and South Asia (WolfSyria, WolfSaudiArabia, Wolf07Israel, Wolf19India). However, the values of these statistics are in the direction of SAT29 being closer to other wolves than to these wolves, and thus likely reflects some basal ancestry in these present-day wolves, e.g. due to admixture

from non-wolf canids. B) Statistics of the form f_4(CoyoteCalifornia,A;B,SAT29.*Canis*) are all negative, indicating that all of the present-day wolves and dogs share genetic drift that is not shared with the SAT29 sample.

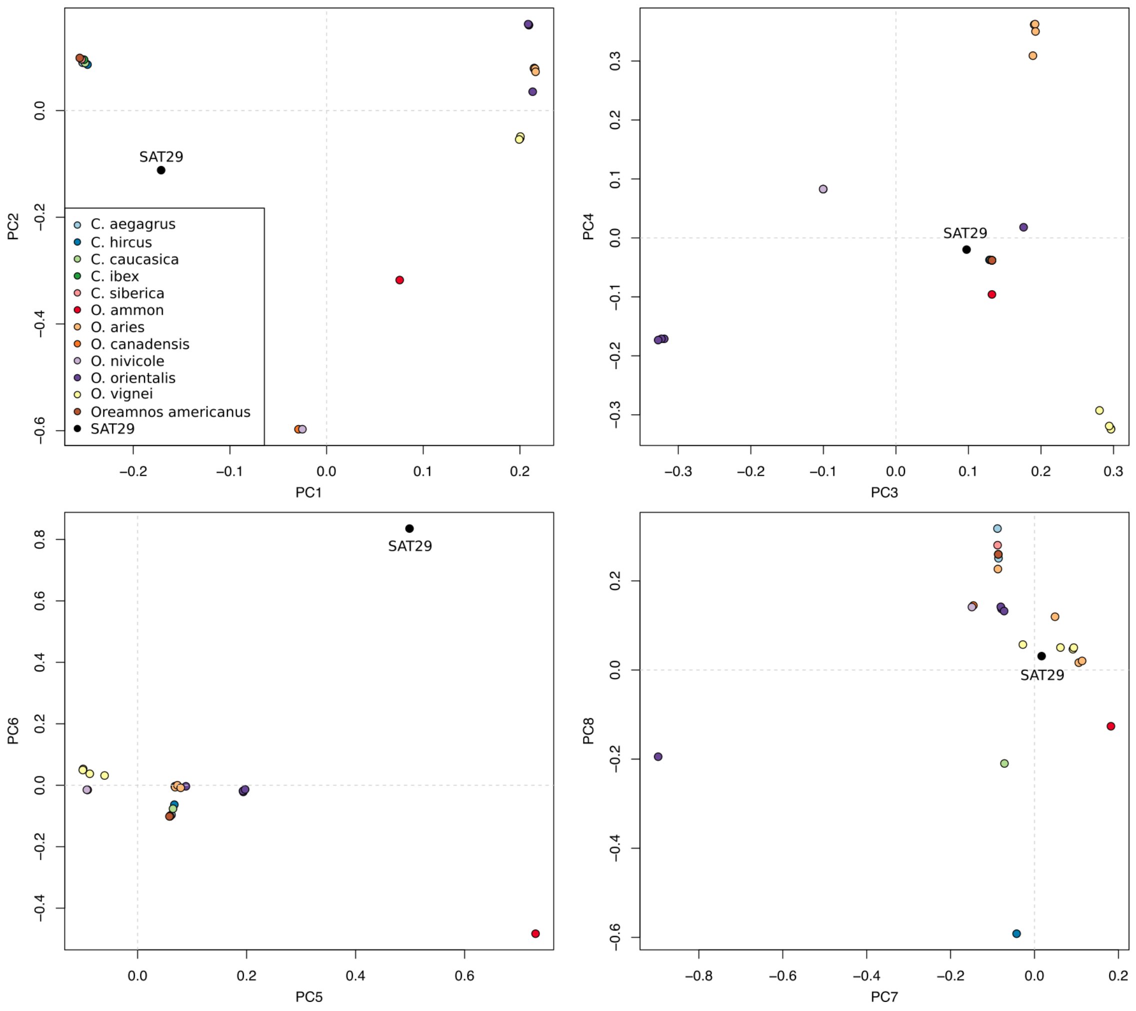

Figure S6: Principal component analysis on the *Ovis* and *Capra* dataset, Related to Figure 2: The analysis was performed on a distance matrix comparing per-sample profiles of all possible f_4-statistics of the form f_4(X,A;B,C) where X is the focal sample.

Supplementary References

S1. Pinhasi, R., Meshveliani, T., Matskevich, Z., Bar-Oz, G., Weissbrod, L., Miller, C.E., Wilkinson, K., Lordkipanidze, D., Jakeli, N., Kvavadze, E., et al. (2014). Satsurblia: new insights of human response and survival across the Last Glacial Maximum in the southern Caucasus. PLoS One *9*, e111271.

S2. Lazaridis, I., Patterson, N., Mittnik, A., Renaud, G., Mallick, S., Kirsanow, K., Sudmant, P.H., Schraiber, J.G., Castellano, S., Lipson, M., et al. (2014). Ancient human genomes suggest three ancestral populations for present-day Europeans. Nature *513*, 409–413.

S3. Mathieson, I., Lazaridis, I., Rohland, N., Mallick, S., Patterson, N., Roodenberg, S.A., Harney, E., Stewardson, K., Fernandes, D., Novak, M., et al. (2015). Genome-wide patterns of selection in 230 ancient Eurasians. Nature *528*, 499–503.

S4. Feldman, M., Fernández-Domínguez, E., Reynolds, L., Baird, D., Pearson, J., Hershkovitz, I., May, H., Goring-Morris, N., Benz, M., Gresky, J., et al. (2019). Late Pleistocene human genome suggests a local origin for the first farmers of central Anatolia. Nat. Commun. *10*, 1218.

S5. Yu, H., Spyrou, M.A., Karapetian, M., Shnaider, S., Radzevičiūtė, R., Nägele, K., Neumann, G.U., Penske, S., Zech, J., Lucas, M., et al. (2020). Paleolithic to Bronze Age Siberians Reveal Connections with First Americans and across Eurasia. Cell.

S6. Jones, E.R., Gonzalez-Fortes, G., Connell, S., Siska, V., Eriksson, A., Martiniano, R., McLaughlin, R.L., Gallego Llorente, M., Cassidy, L.M., Gamba, C., et al. (2015). Upper Palaeolithic genomes reveal deep roots of modern Eurasians. Nat. Commun. *6*, 8912.

S7. Lazaridis, I., Belfer-Cohen, A., Mallick, S., Patterson, N., Cheronet, O., Rohland, N., Bar-Oz, G., Bar-Yosef, O., Jakeli, N., Kvavadze, E., et al. (2018). Paleolithic DNA from the Caucasus reveals core of West Eurasian ancestry. bioRxiv, 423079.

S8. Yang, M.A., Gao, X., Theunert, C., Tong, H., Aximu-Petri, A., Nickel, B., Slatkin, M., Meyer, M., Pääbo, S., Kelso, J., et al. (2017). 40,000-Year-Old Individual from Asia Provides Insight into Early Population Structure in Eurasia. Curr. Biol. *27*, 3202–3208.e9.

S9. Fu, Q., Posth, C., Hajdinjak, M., Petr, M., Mallick, S., Fernandes, D., Furtwängler, A., Haak, W., Meyer, M., Mittnik, A., et al. (2016). The genetic history of Ice Age Europe. Nature *534*, 200–205.

S10. Olalde, I., Allentoft, M.E., Sánchez-Quinto, F., Santpere, G., Chiang, C.W.K., DeGiorgio, M., Prado-Martinez, J., Rodríguez, J.A., Rasmussen, S., Quilez, J., et al. (2014). Derived immune and ancestral pigmentation alleles in a 7,000-year-old Mesolithic European. Nature *507*, 225–228.

S11. Narasimhan, V.M., Patterson, N., Moorjani, P., Rohland, N., Bernardos, R., Mallick, S., Lazaridis, I., Nakatsuka, N., Olalde, I., Lipson, M., et al. (2019). The formation of human populations in South and Central Asia. Science *365*.

S12. Lazaridis, I., Nadel, D., Rollefson, G., Merrett, D.C., Rohland, N., Mallick, S., Fernandes, D., Novak, M., Gamarra, B., Sirak, K., et al. (2016). Genomic insights into the origin of farming in the ancient Near East. Nature *536*, 419–424.

S13. van de Loosdrecht, M., Bouzouggar, A., Humphrey, L., Posth, C., Barton, N., Aximu-Petri, A., Nickel, B., Nagel, S., Talbi, E.H., El Hajraoui, M.A., et al. (2018). Pleistocene North African genomes link Near Eastern and sub-Saharan African human populations. Science *360*, 548.

S14. Fu, Q., Hajdinjak, M., Moldovan, O.T., Constantin, S., Mallick, S., Skoglund, P., Patterson, N., Rohland, N., Lazaridis, I., Nickel, B., et al. (2015). An early modern human from Romania with a recent Neanderthal ancestor. Nature *524*, 216–219.

S15. Fu, Q., Li, H., Moorjani, P., Jay, F., Slepchenko, S.M., Bondarev, A.A., Johnson, P.L.F., Aximu-Petri, A., Prüfer, K., de Filippo, C., et al. (2014). Genome sequence of a 45,000-year-old modern human from western Siberia. Nature *514*, 445–449.

S16. Raghavan, M., Skoglund, P., Graf, K.E., Metspalu, M., Albrechtsen, A., Moltke, I., Rasmussen, S., Stafford, T.W., Jr, Orlando, L., Metspalu, E., et al. (2014). Upper Palaeolithic Siberian genome reveals dual ancestry of Native Americans. Nature *505*, 87–91.

S17. Seguin-Orlando, A., Korneliussen, T.S., Sikora, M., Malaspinas, A.-S., Manica, A., Moltke, I., Albrechtsen, A., Ko, A., Margaryan, A., Moiseyev, V., et al. (2014). Paleogenomics. Genomic structure in Europeans dating back at least 36,200 years. Science *346*, 1113–1118.

S18. Sikora, M., Seguin-Orlando, A., Sousa, V.C., Albrechtsen, A., Korneliussen, T., Ko, A., Rasmussen, S., Dupanloup, I., Nigst, P.R., Bosch, M.D., et al. (2017). Ancient genomes show social and reproductive behavior of early Upper Paleolithic foragers. Science *358*, 659–662.

S19. Gamba, C., Jones, E.R., Teasdale, M.D., McLaughlin, R.L., Gonzalez-Fortes, G., Mattiangeli, V., Domboróczki, L., Kővári, I., Pap, I., Anders, A., et al. (2014). Genome flux and stasis in a five millennium transect of European prehistory. Nat. Commun. *5*, 5257.

S20. Hajdinjak, M., Mafessoni, F., Skov, L., Vernot, B., Hübner, A., Fu, Q., Essel, E., Nagel, S., Nickel, B., Richter, J., et al. (2021). Initial Upper Palaeolithic humans in Europe had recent Neanderthal ancestry. Nature *592*, 253–257.

S21. Prüfer, K., Posth, C., Yu, H., Stoessel, A., Spyrou, M.A., Deviese, T., Mattonai, M., Ribechini, E., Higham, T., Velemínský, P., et al. (2021). A genome sequence from a modern human skull over 45,000 years old from Zlatý kůň in Czechia. Nat Ecol Evol.

S22. Posth, C., Renaud, G., Mittnik, A., Drucker, D.G., Rougier, H., Cupillard, C., Valentin, F., Thevenet, C., Furtwängler, A., Wißing, C., et al. (2016). Pleistocene Mitochondrial Genomes Suggest a Single Major Dispersal of Non-Africans and a Late Glacial Population Turnover in Europe. Curr. Biol. *26*, 827–833.

S23. Bollongino, R., Nehlich, O., Richards, M.P., Orschiedt, J., Thomas, M.G., Sell, C., Fajkosová, Z., Powell, A., and Burger, J. (2013). 2000 years of parallel societies in Stone Age Central Europe. Science *342*, 479–481.

S24. Fu, Q., Mittnik, A., Johnson, P.L.F., Bos, K., Lari, M., Bollongino, R., Sun, C., Giemsch, L., Schmitz, R., Burger, J., et al. (2013). A revised timescale for human evolution based on ancient mitochondrial genomes. Curr. Biol. *23*, 553–559.

S25. Fu, Q., Meyer, M., Gao, X., Stenzel, U., Burbano, H.A., Kelso, J., and Pääbo, S. (2013). DNA analysis of an early modern human from Tianyuan Cave, China. Proc. Natl. Acad. Sci. U. S. A. *110*, 2223–2227.

S26. Benazzi, S., Slon, V., Talamo, S., Negrino, F., Peresani, M., Bailey, S.E., Sawyer, S., Panetta, D., Vicino, G., Starnini, E., et al. (2015). Archaeology. The makers of the Protoaurignacian and implications for Neandertal extinction. Science *348*, 793–796.

S27. Ermini, L., Olivieri, C., Rizzi, E., Corti, G., Bonnal, R., Soares, P., Luciani, S., Marota, I., De Bellis, G., Richards, M.B., et al. (2008). Complete mitochondrial genome sequence of the Tyrolean Iceman. Curr. Biol. *18*, 1687–1693.

S28. Gilbert, M.T.P., Jenkins, D.L., Götherstrom, A., Naveran, N., Sanchez, J.J., Hofreiter, M., Thomsen, P.F., Binladen, J., Higham, T.F.G., Yohe, R.M., 2nd, et al. (2008). DNA from pre-Clovis human coprolites in Oregon, North America. Science *320*, 786–789.

S29. Günther, T., Malmström, H., Svensson, E.M., Omrak, A., Sánchez-Quinto, F., Kılınç, G.M., Krzewińska, M., Eriksson, G., Fraser, M., Edlund, H., et al. (2018). Population genomics of Mesolithic Scandinavia: Investigating early postglacial migration routes and high-latitude adaptation. PLoS Biol. *16*, e2003703.

S30. Vai, S., Sarno, S., Lari, M., Luiselli, D., Manzi, G., Gallinaro, M., Mataich, S., Hübner, A., Modi, A., Pilli, E., et al. (2019). Ancestral mitochondrial N lineage from the Neolithic "green" Sahara. Sci. Rep. *9*, 3530.

S31. Hublin, J.-J., Sirakov, N., Aldeias, V., Bailey, S., Bard, E., Delvigne, V., Endarova, E., Fagault, Y., Fewlass, H., Hajdinjak, M., et al. (2020). Initial Upper Palaeolithic Homo sapiens from Bacho Kiro Cave, Bulgaria. Nature, 1–4.

S32. Soares, P., Alshamali, F., Pereira, J.B., Fernandes, V., Silva, N.M., Afonso, C., Costa, M.D., Musilová, E., Macaulay, V., Richards, M.B., et al. (2012). The Expansion of mtDNA Haplogroup L3 within and out of Africa. Mol. Biol. Evol. *29*, 915–927.

S33. Gonder, M.K., Mortensen, H.M., Reed, F.A., de Sousa, A., and Tishkoff, S.A. (2007). Whole-mtDNA genome sequence analysis of ancient African lineages. Mol. Biol. Evol. *24*, 757–768.

S34. Torroni, A., Achilli, A., Macaulay, V., Richards, M., and Bandelt, H.-J. (2006). Harvesting the fruit of the human mtDNA tree. Trends Genet. *22*, 339–345.

S35. Behar, D.M., Villems, R., Soodyall, H., Blue-Smith, J., Pereira, L., Metspalu, E., Scozzari,

R., Makkan, H., Tzur, S., Comas, D., et al. (2008). The dawn of human matrilineal diversity. Am. J. Hum. Genet. *82*, 1130–1140.

S36. Barbieri, E., and Sestili, P. (2012). Reactive oxygen species in skeletal muscle signaling. J. Signal Transduct. *2012*, 982794.

S37. Behar, D.M., van Oven, M., Rosset, S., Metspalu, M., Loogväli, E.-L., Silva, N.M., Kivisild, T., Torroni, A., and Villems, R. (2012). A "Copernican" reassessment of the human mitochondrial DNA tree from its root. Am. J. Hum. Genet. *90*, 675–684.

S38. Derenko, M., Malyarchuk, B., Bahmanimehr, A., Denisova, G., Perkova, M., Farjadian, S., and Yepiskoposyan, L. (2013). Complete mitochondrial DNA diversity in Iranians. PLoS One *8*, e80673.

S39. Harich, N., Costa, M.D., Fernandes, V., Kandil, M., Pereira, J.B., Silva, N.M., and Pereira, L. (2010). The trans-Saharan slave trade - clues from interpolation analyses and high-resolution characterization of mitochondrial DNA lineages. BMC Evol. Biol. *10*, 138.

S40. Cerný, V., Fernandes, V., Costa, M.D., Hájek, M., Mulligan, C.J., and Pereira, L. (2009). Migration of Chadic speaking pastoralists within Africa based on population structure of Chad Basin and phylogeography of mitochondrial L3f haplogroup. BMC Evol. Biol. *9*, 63.

S41. Batini, C., Lopes, J., Behar, D.M., Calafell, F., Jorde, L.B., van der Veen, L., Quintana-Murci, L., Spedini, G., Destro-Bisol, G., and Comas, D. (2011). Insights into the demographic history of African Pygmies from complete mitochondrial genomes. Mol. Biol. Evol. *28*, 1099–1110.

S42. Ingman, M., Kaessmann, H., Pääbo, S., and Gyllensten, U. (2000). Mitochondrial genome variation and the origin of modern humans. Nature *408*, 708–713.

S43. Podgorná, E., Soares, P., Pereira, L., and Cerný, V. (2013). The genetic impact of the lake chad basin population in North Africa as documented by mitochondrial diversity and internal variation of the L3e5 haplogroup. Ann. Hum. Genet. *77*, 513–523.

S44. Vyas, S., Zaganjor, E., and Haigis, M.C. (2016). Mitochondria and Cancer. Cell *166*, 555–566.

S45. Pennarun, E., Kivisild, T., Metspalu, E., Metspalu, M., Reisberg, T., Moisan, J.-P., Behar, D.M., Jones, S.C., and Villems, R. (2012). Divorcing the Late Upper Palaeolithic demographic histories of mtDNA haplogroups M1 and U6 in Africa. BMC Evol. Biol. *12*, 234.

S46. Olivieri, A., Achilli, A., Pala, M., Battaglia, V., Fornarino, S., Al-Zahery, N., Scozzari, R., Cruciani, F., Behar, D.M., Dugoujon, J.-M., et al. (2006). The mtDNA legacy of the Levantine early Upper Palaeolithic in Africa. Science *314*, 1767–1770.

S47. González, A.M., Larruga, J.M., Abu-Amero, K.K., Shi, Y., Pestano, J., and Cabrera, V.M. (2007). Mitochondrial lineage M1 traces an early human backflow to Africa. BMC Genomics *8*, 223.

S48. Fernandes, V., Alshamali, F., Alves, M., Costa, M.D., Pereira, J.B., Silva, N.M., Cherni, L., Harich, N., Cerny, V., Soares, P., et al. (2012). The Arabian cradle: mitochondrial relicts of the first steps along the southern route out of Africa. Am. J. Hum. Genet. *90*, 347–355.

S49. Olivieri, A., Pala, M., Gandini, F., Hooshiar Kashani, B., Perego, U.A., Woodward, S.R., Grugni, V., Battaglia, V., Semino, O., Achilli, A., et al. (2013). Mitogenomes from two uncommon haplogroups mark late glacial/postglacial expansions from the near east and neolithic dispersals within Europe. PLoS One *8*, e70492.

S50. Greenspan, B. (2008). Family Tree DNA.

S51. Janssen, A.J.M., Trijbels, F.J.M., Sengers, R.C.A., Wintjes, L.T.M., Ruitenbeek, W., Smeitink, J.A.M., Morava, E., van Engelen, B.G.M., van den Heuvel, L.P., and Rodenburg, R.J.T. (2006). Measurement of the energy-generating capacity of human muscle mitochondria: diagnostic procedure and application to human pathology. Clin. Chem. *52*, 860–871.

S52. Schönberg, A., Theunert, C., Li, M., Stoneking, M., and Nasidze, I. (2011). High-throughput sequencing of complete human mtDNA genomes from the Caucasus and West Asia: high diversity and demographic inferences. Eur. J. Hum. Genet. *19*, 988–994.

S53. Kushniarevich, A., Sivitskaya, L., Danilenko, N., Novogrodskii, T., Tsybovsky, I., Kiseleva, A., Kotova, S., Chaubey, G., Metspalu, E., Sahakyan, H., et al. (2013). Uniparental genetic heritage of belarusians: encounter of rare middle eastern matrilineages with a central European mitochondrial DNA pool. PLoS One *8*, e66499.

S54. Costa, M.D., Pereira, J.B., Pala, M., Fernandes, V., Olivieri, A., Achilli, A., Perego, U.A., Rychkov, S., Naumova, O., Hatina, J., et al. (2013). A substantial prehistoric European ancestry amongst Ashkenazi maternal lineages. Nat. Commun. *4*, 1–10.

S55. Derbeneva, O.A., Starikovskaya, E.B., Wallace, D.C., and Sukernik, R.I. (2002). Traces of early Eurasians in the Mansi of northwest Siberia revealed by mitochondrial DNA analysis. Am. J. Hum. Genet. *70*, 1009–1014.

S56. Shlush, L.I., Atzmon, G., Weisshof, R., Behar, D., Yudkovsky, G., Barzilai, N., and Skorecki, K. (2008). Ashkenazi Jewish centenarians do not demonstrate enrichment in mitochondrial haplogroup J. PLoS One *3*, e3425.

S57. Kujanová, M., Pereira, L., Fernandes, V., Pereira, J.B., and Cerný, V. (2009). Near eastern neolithic genetic input in a small oasis of the Egyptian Western Desert. Am. J. Phys. Anthropol. *140*, 336–346.

S58. Costa, M.D., Cherni, L., Fernandes, V., Freitas, F., Ammar El Gaaied, A.B., and Pereira, L. (2009). Data from complete mtDNA sequencing of Tunisian centenarians: testing haplogroup association and the “golden mean” to longevity. Mech. Ageing Dev. *130*, 222–226.

S59. Fraumene, C., Belle, E.M.S., Castrì, L., Sanna, S., Mancosu, G., Cosso, M., Marras, F., Barbujani, G., Pirastu, M., and Angius, A. (2006). High resolution analysis and phylogenetic

network construction using complete mtDNA sequences in sardinian genetic isolates. Mol. Biol. Evol. *23*, 2101–2111.

S60. Raule, N., Sevini, F., Li, S., Barbieri, A., Tallaro, F., Lomartire, L., Vianello, D., Montesanto, A., Moilanen, J.S., Bezrukov, V., et al. (2014). The co-occurrence of mtDNA mutations on different oxidative phosphorylation subunits, not detected by haplogroup analysis, affects human longevity and is population specific. Aging Cell *13*, 401–407.

S61. Achilli, A., Olivieri, A., Pala, M., Hooshiar Kashani, B., Carossa, V., Perego, U.A., Gandini, F., Santoro, A., Battaglia, V., Grugni, V., et al. (2011). Mitochondrial DNA backgrounds might modulate diabetes complications rather than T2DM as a whole. PLoS One *6*, e21029.

S62. Pereira, L., Gonçalves, J., Franco-Duarte, R., Silva, J., Rocha, T., Arnold, C., Richards, M., and Macaulay, V. (2007). No evidence for an mtDNA role in sperm motility: data from complete sequencing of asthenozoospermic males. Mol. Biol. Evol. *24*, 868–874.

S63. Thalmann, O., Shapiro, B., Cui, P., Schuenemann, V.J., Sawyer, S.K., Greenfield, D.L., Germonpré, M.B., Sablin, M.V., López-Giráldez, F., Domingo-Roura, X., et al. (2013). Complete mitochondrial genomes of ancient canids suggest a European origin of domestic dogs. Science *342*, 871–874.

S64. Loog, L., Thalmann, O., Sinding, M.-H.S., Schuenemann, V.J., Perri, A., Germonpré, M., Bocherens, H., Witt, K.E., Samaniego Castruita, J.A., Velasco, M.S., et al. (2020). Ancient DNA suggests modern wolves trace their origin to a Late Pleistocene expansion from Beringia. Mol. Ecol. *29*, 1596–1610.

S65. Skoglund, P., Ersmark, E., Palkopoulou, E., and Dalén, L. (2015). Ancient wolf genome reveals an early divergence of domestic dog ancestors and admixture into high-latitude breeds. Curr. Biol. *25*, 1515–1519.

S66. Matsumura, S., Inoshima, Y., and Ishiguro, N. (2014). Reconstructing the colonization history of lost wolf lineages by the analysis of the mitochondrial genome. Mol. Phylogenet. Evol. *80*, 105–112.

S67. Björnerfeldt, S., Webster, M.T., and Vilà, C. (2006). Relaxation of selective constraint on dog mitochondrial DNA following domestication. Genome Res. *16*, 990–994.

S68. Chen, L., and Zhang, H.H. (2009). Canis lupus chanco mitochondrion, complete genome.

S69. Zhang, H., Zhang, J., Chen, L., and Liu, G. (2014). The complete mitochondrial genome of Chinese Shinjang wolf. Mitochondrial DNA *25*, 106–108.

S70. Pang, J.-F., Kluetsch, C., Zou, X.-J., Zhang, A.-B., Luo, L.-Y., Angleby, H., Ardalan, A., Ekström, C., Sköllermo, A., Lundeberg, J., et al. (2009). mtDNA data indicate a single origin for dogs south of Yangtze River, less than 16,300 years ago, from numerous wolves. Mol. Biol. Evol. *26*, 2849–2864.

S71. Arnason, U., Gullberg, A., Janke, A., and Kullberg, M. (2007). Mitogenomic analyses of caniform relationships. Mol. Phylogenet. Evol. *45*, 863–874.

S72. Arnason, U., Gullberg, A., Janke, A., and Kullberg, M. (2007). Mitogenomic analyses of caniform relationships. Mol. Phylogenet. Evol. *45*, 863–874.

S73. Gopalakrishnan, S., Sinding, M.-H.S., Ramos-Madrigal, J., Niemann, J., Samaniego Castruita, J.A., Vieira, F.G., Carøe, C., Montero, M. de M., Kuderna, L., Serres, A., et al. (2018). Interspecific Gene Flow Shaped the Evolution of the Genus Canis. Curr. Biol. *28*, 3441–3449.e5.

S74. Hassanin, A., Delsuc, F., Ropiquet, A., Hammer, C., Jansen van Vuuren, B., Matthee, C., Ruiz-Garcia, M., Catzeflis, F., Areskoug, V., Nguyen, T.T., et al. (2012). Pattern and timing of diversification of Cetartiodactyla (Mammalia, Laurasiatheria), as revealed by a comprehensive analysis of mitochondrial genomes. C. R. Biol. *335*, 32–50.

S75. Douglas, K.C., Halbert, N.D., Kolenda, C., Childers, C., Hunter, D.L., and Derr, J.N. (2011). Complete mitochondrial DNA sequence analysis of Bison bison and bison-cattle hybrids: function and phylogeny. Mitochondrion *11*, 166–175.

S76. Zeyland, J., Wolko, L., Bocianowski, J., Szalata, M., Słomski, R., Dzieduszycki, A.M., Ryba, M., Przystałowska, H., and Lipiński, D. (2013). Complete mitochondrial genome of wild aurochs (Bos primigenius) reconstructed from ancient DNA. Pol. J. Vet. Sci. *16*, 265–273.

S77. Zhang, H., Paijmans, J.L.A., Chang, F., Wu, X., Chen, G., Lei, C., Yang, X., Wei, Z., Bradley, D.G., Orlando, L., et al. (2013). Morphological and genetic evidence for early Holocene cattle management in northeastern China. Nat. Commun. *4*, 2755.

S78. Huang, C., Zhang, Q., Wu, X., Dawa, Y., Fu, D., Jiang, H., Chu, M., Dingkao, R., Guo, X., Basang, W., et al. (2019). The complete mitochondrial genome sequence and phylogenetic analysis of Niangya yak (Bos grunniens). Mitochondrial DNA B Resour *5*, 202–203.

S79. Chunnian, L., Wu, X., Ding, X., Wang, H., Guo, X., Chu, M., Bao, P., and Yan, P. (2016). Characterization of the complete mitochondrial genome sequence of wild yak (Bos mutus). Mitochondrial DNA A DNA Mapp Seq Anal *27*, 4266–4267.

S80. Kirillova, I.V., Zanina, O.G., Chernova, O.F., Lapteva, E.G., Trofimova, S.S., Lebedev, V.S., Tiunov, A.V., Soares, A.E.R., Shidlovskiy, F.K., and Shapiro, B. (2015). An ancient bison from the mouth of the Rauchua River (Chukotka, Russia). Quat. Res. *84*, 232–245.

S81. Węcek, K., Hartmann, S., Paijmans, J.L.A., Taron, U., Xenikoudakis, G., Cahill, J.A., Heintzman, P.D., Shapiro, B., Baryshnikov, G., Bunevich, A.N., et al. (2016). Complex Admixture Preceded and Followed the Extinction of Wisent in the Wild. Mol. Biol. Evol. *34*, 598–612.

S82. Soubrier, J., Gower, G., Chen, K., Richards, S.M., Llamas, B., Mitchell, K.J., Ho, S.Y.W., Kosintsev, P., Lee, M.S.Y., Baryshnikov, G., et al. (2016). Early cave art and ancient DNA record the origin of European bison. Nat. Commun. *7*, 13158.

S83. Massilani, D., Guimaraes, S., Brugal, J.-P., Bennett, E.A., Tokarska, M., Arbogast, R.-M., Baryshnikov, G., Boeskorov, G., Castel, J.-C., Davydov, S., et al. (2016). Past climate changes, population dynamics and the origin of Bison in Europe. BMC Biol. *14*, 93.

S84. Miretti, M.M., Pereira, H.A., Jr, Poli, M.A., Contel, E.P.B., and Ferro, J.A. (2002). African-derived mitochondria in South American native cattle breeds (Bos taurus): evidence of a new taurine mitochondrial lineage. J. Hered. *93*, 323–330.

S85. Qian, J.X., Dong, K.J., Huang, Y.J., Yang, B.Z., He, M., Liu, Z.J., and Li, J. (2004). Bubalus carabanensis mitochondrion, complete genome.

S86. San, A., Wang, W.A., and Yu, L. (2007). Bos grunniens mitochondrion, complete genome.

S87. Chung, H.Y., and Ha, J.M. (2005). Bos taurus mitochondrion, complete genome.

S88. Achilli, A., Olivieri, A., Pellecchia, M., Uboldi, C., Colli, L., Al-Zahery, N., Accetturo, M., Pala, M., Hooshiar Kashani, B., Perego, U.A., et al. (2008). Mitochondrial genomes of extinct aurochs survive in domestic cattle. Curr. Biol. *18*, R157–8.

S89. Lipinski, D., Zeyland, J., Kowalska, M., Szalata, M., Dzieduszycki, A.M., Ryba, M.S., and Slomski, R. (2009). Bos javanicus mitochondrion, complete genome.

S90. Edwards, C.J., Magee, D.A., Park, S.D.E., McGettigan, P.A., Lohan, A.J., Murphy, A., Finlay, E.K., Shapiro, B., Chamberlain, A.T., Richards, M.B., et al. (2010). A complete mitochondrial genome sequence from a mesolithic wild aurochs (Bos primigenius). PLoS One *5*, e9255.

S91. Zeyland, J., Wolko, L., Lipiński, D., Woźniak, A., Nowak, A., Szalata, M., Bocianowski, J., and Słomski, R. (2012). Tracking of wisent-bison-yak mitochondrial evolution. J. Appl. Genet. *53*, 317–322.

S92. Na, R.-S., Zhao, Y.-J., Gao, H.-J., An, T.-W., Huang, Y.-F., and E, G.-X. (2016). Complete mitochondrial genome of the Yakow (Bos primigenius taurus × Bos grunniens) in China. Mitochondrial DNA A DNA Mapp Seq Anal *27*, 3826–3827.

S93. Marsolier-Kergoat, M.-C., Palacio, P., Berthonaud, V., Maksud, F., Stafford, T., Bégouën, R., and Elalouf, J.-M. (2015). Hunting the Extinct Steppe Bison (Bison priscus) Mitochondrial Genome in the Trois-Frères Paleolithic Painted Cave. PLoS One *10*, e0128267.

S94. Derr, J.N., Halbert, N.D., Gogan, P., and Douglas, K.C. (2016). Bison bonasus mitochondrion, complete genome.
