# Supplementary material for: Genome-scale sequencing and analysis of human, wolf, and bison DNA from 25,000-year-old sediment
Source: Curr Biol. 2021 Aug 23;31(16):3564–3574.e9. doi: 10.1016/j.cub.2021.06.023 (PMC8409484; doi:10.1016/j.cub.2021.06.023)
Supplement: Document S2. Article plus supplemental information [file mmc8.pdf]

Current Biology

Article

Genome-scale sequencing and analysis of human, wolf, and bison DNA from 25,000-year-old sediment

Highlights

- A single shotgun-sequenced Pleistocene sediment yielded genomic data of three mammals
- Sediment genome sequencing can produce data comparable to that from skeletal remains
- A pre-LGM human lineage from the Caucasus was an ancestral component of West Eurasia
- ~0.01X wolf and bison environmental genomes, suggesting reshaping of populations

Authors

Pere Gelabert, Susanna Sawyer, Anders Bergström, ..., Mareike C. Stahlschmidt, Pontus Skoglund, Ron Pinhasi

Correspondence

pere.gelabert@univie.ac.at (P.G.),
anders.bergstrom@crick.ac.uk (A.B.),
pontus.skoglund@crick.ac.uk (P.S.),
ron.pinhasi@univie.ac.at (R.P.)

In brief

Cave sediments preserve ancient DNA for thousands of years. Gelabert et al. retrieve human and mammalian nuclear and mitochondrial environmental "shotgun" genomes from a single 25,000-year-old sediment sample from Satsurblia cave. Their results provide insights into the population genetic histories of three mammalian species.

Gelabert et al., 2021, Current Biology *31*, 3564–3574
August 23, 2021 © 2021 The Authors. Published by Elsevier Inc.
https://doi.org/10.1016/j.cub.2021.06.023

CellPress

CellPress
OPEN ACCESS

Current Biology

Article

Genome-scale sequencing and analysis of human, wolf, and bison DNA from 25,000-year-old sediment

Pere Gelabert,[1,11,13,*] Susanna Sawyer,[1,11] Anders Bergström,[2,11,*] Ashot Margaryan,[3] Thomas C. Collin,[4] Tengiz Meshveliani,[5] Anna Belfer-Cohen,[6] David Lordkipanidze,[5] Nino Jakeli,[5] Zinovi Matskevich,[7] Guy Bar-Oz,[8] Daniel M. Fernandes,[1,9] Olivia Cheronet,[1] Kadir T. Özdoğan,[1] Victoria Oberreiter,[1] Robin N.M. Feeney,[4] Mareike C. Stahlschmidt,[10] Pontus Skoglund,[2,12,*] and Ron Pinhasi[1,12,*]

[1]Department of Evolutionary Anthropology, University of Vienna, Vienna, Austria
[2]Ancient Genomics Laboratory, Francis Crick Institute, London, UK
[3]Center for Evolutionary Hologenomics, University of Copenhagen, Copenhagen, Denmark
[4]School of Medicine, University College Dublin, Dublin, Ireland
[5]Georgian National Museum, Institute of Paleoanthropology and Paleobiology, Tbilisi, Georgia
[6]Institute of Archaeology, The Hebrew University of Jerusalem, Jerusalem, Israel
[7]Israel Antiquities Authority, Jerusalem, Israel
[8]Zinman Institute of Archaeology, University of Haifa, Haifa, Israel
[9]CIAS, Department of Life Sciences, University of Coimbra, Coimbra, Portugal
[10]Department of Human Evolution, Max-Planck-Institute for Evolutionary Anthropology, Leipzig, Germany
[11]These authors contributed equally
[12]These authors contributed equally
[13]Lead contact
*Correspondence: pere.gelabert@univie.ac.at (P.G.), anders.bergstrom@crick.ac.uk (A.B.), pontus.skoglund@crick.ac.uk (P.S.), ron.pinhasi@univie.ac.at (R.P.)
https://doi.org/10.1016/j.cub.2021.06.023

SUMMARY

Cave sediments have been shown to preserve ancient DNA but so far have not yielded the genome-scale information of skeletal remains. We retrieved and analyzed human and mammalian nuclear and mitochondrial environmental "shotgun" genomes from a single 25,000-year-old Upper Paleolithic sediment sample from Satsurblia cave, western Georgia:first, a human environmental genome with substantial basal Eurasian ancestry, which was an ancestral component of the majority of post-Ice Age people in the Near East, North Africa, and parts of Europe; second, a wolf environmental genome that is basal to extant Eurasian wolves and dogs and represents a previously unknown, likely extinct, Caucasian lineage; and third, a European bison environmental genome that is basal to present-day populations, suggesting that population structure has been substantially reshaped since the Last Glacial Maximum. Our results provide new insights into the Late Pleistocene genetic histories of these three species and demonstrate that direct shotgun sequencing of sediment DNA, without target enrichment methods, can yield genome-wide data informative of ancestry and phylogenetic relationships.

INTRODUCTION

Ancient DNA fragments sequenced from bone,[1] teeth,[2] and hair[3] have revolutionized our understanding of natural history and the human past.[4,5] When skeletal material is not available, ancient environmental DNA has been used to determine the presence or absence of different species. Several studies based on PCR methods demonstrated the presence of ancient DNA in sediments,[6] including in caves,[7] and more recently, high throughput sequencing techniques have been applied.[8–11] Cave sediment ancient DNA has been used to track the presence or absence of species across a range of environments and time periods, primarily through targeted amplification or capture of single genetic regions.[12] A ground-breaking study showed DNA preservation in clay-rich sediments since ~240 ky[13] and used targeted enrichment to recover sufficient numbers of fragments to reconstruct mtDNA phylogenies of Neanderthals and Denisovans. A similar study recovered Denisovan mitochondrial DNA from sediments deposited ~100 kya and ~60 kya from Baishya Karst Cave on the Tibetan Plateau.[14] A recent study used targeted enrichment of 1.6 million loci to recover Neanderthal and Denisovan nuclear DNA from three Paleolithic sites. This yielded enough DNA to allow for some analyses of genome-wide ancestry, including the finding of a Neanderthal population replacement at one of the sites, thereby demonstrating the possibility of large-scale nuclear DNA recovery from sediments.[15]

Here, we report results from shotgun sequencing and genomic analysis of a sediment sample from the Upper Paleolithic site of

 © 2021 The Authors. Published by Elsevier Inc.
This is an open access article under the CC BY license (http://creativecommons.org/licenses/by/4.0/).

CellPress
OPEN ACCESS

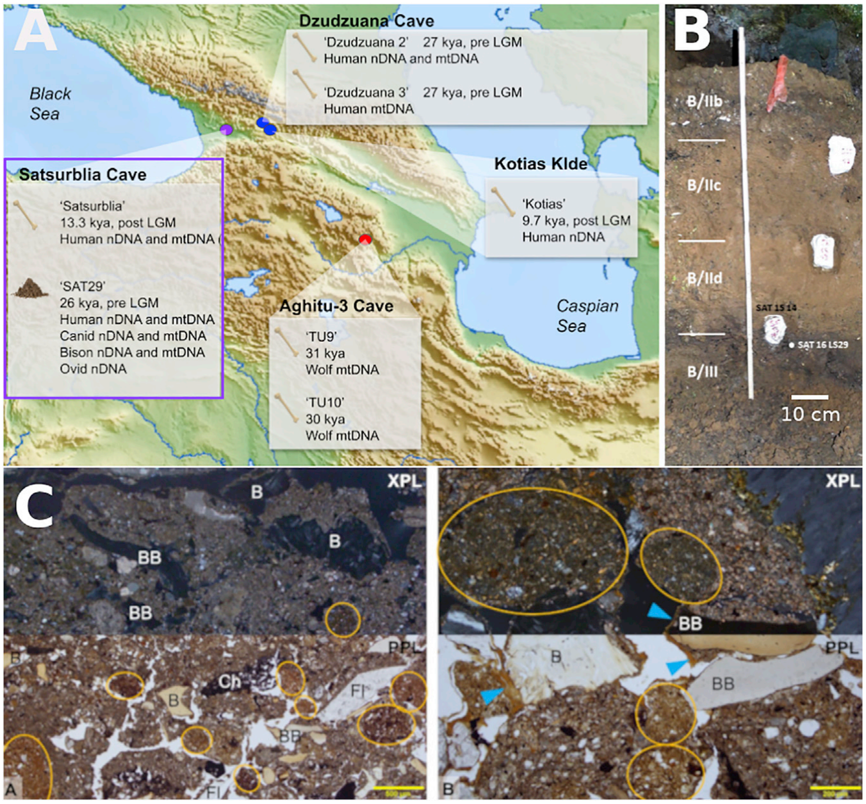

Figure 1. Site description: an overview of the location of Satsurblia cave with contextual information

(A) Map of the Caucasus region showing relevant sites that have yielded ancient DNA from humans (blue dots), animals (red dot), or both (purple dot). Only sites with remains from the Mesolithic or older are shown.

(B) Layer B of Satsurblia cave with the SAT29 sampling location shown.

(C) Two microphotographs (A and B) from block sample SAT 15 14. The microphotographs were taken adjacent to SAT 16 LS29, in cross- (XPL) and plane-polarized light (PPL), showing dominant components and processes. Typical anthropogenic components are bone (B), burnt bone (BB), charcoal (Ch), and flint (Fl). Note also the occurrence of rounded soil aggregates (yellow circles) that were transported into the cave from soils forming outside and that exhibit cross-striations of the clay component resulting from repeated wetting and drying cycles during their formation. Similarly, clay coatings in voids (blue arrow) result from water percolating through the sediment.

See also Data S1.

Satsurblia Cave, southern Caucasus, dating to the Last Glacial Maximum (LGM, 25,000 years ago [kya]). In most of the Caucasus, and particularly in western Georgia, karst systems hold low and stable year-round temperatures and low acidity (no guano deposits in most systems). The sediment sample yielded up to several million sequence reads from human, wolf (*Canis lupus*), and bison (*Bison bonasus*), corresponding to genome-wide data comparable to low-coverage sequencing obtained from skeletal remains.

RESULTS

We analyzed six sediment samples from different layers of areas A (pre-LGM) and B (pre and post-LGM) of Satsurblia cave (Figure 1A)[16] and performed shotgun sequencing to screen them for mammalian DNA (Data S1A). One of the samples, SAT16 LS29 (SAT29) from layer BIII (Figure 1B), which is radiocarbon dated to 25.4–24.5 ka cal BP,[16] contained substantial amounts of DNA from humans as well as from other mammals and was therefore sequenced to greater depth.

We sequenced 561,263,536 reads from the SAT29 sample, and after filtering, we retained 226,880,778 reads. Metagenomic screening with centrifuge[17] indicated that 1.3% of the reads were of eukaryotic origin, and four main mammalian genera were identified: *Ovis* (28%), *Homo* (9%), *Canis* (5.5%), and *Bos* (2.1%) (Figure 2A). After competitive mapping to the sheep, human, dog, and domestic cattle reference genomes (Method Details), we assigned a total of 4,956,676 reads as follows: *Canis* (2,378,237 reads, 48.0% of assigned reads), *Bos* (1,811,555 reads, 36.5% of assigned reads), *Homo* (661,765 reads, 13.5% of assigned reads), and *Ovis* (105,119 reads, 2.1% of assigned reads). We then used BLAST+ and MEGAN to verify the accuracy of the mapping process and show that it is unlikely that any other animal species are represented in substantial amounts in the sample (Figures 2B, 2C, S1, S2, S3, S4, S5, and S6; Data S1B; Method details).

Mitochondrial capture and evidence for multiple individuals

Through targeted capture,[20] we recovered 3,447 human mitochondrial DNA (mtDNA) reads, which when aligned to rCRS resulted in a 10-fold coverage of the human mtDNA. We similarly recovered 5,809 *Canis* mtDNA reads, yielding a 13-fold coverage of the *C. lupus* mitochondrial genome. Capture of cattle mtDNA also proved successful, but the recovered sequences showed high similarly to bison mitochondria, suggesting that the DNA identified as deriving from *Bos* in the metagenomic screening in fact derived from *Bison sp.* (that was not included in the metagenomic reference set). We recovered 2,448 reads providing an 8-fold coverage of the *B. bonasus* mitochondrial genome. All the recovered environmental genomes show elevated deamination values and short fragment length distributions, consistent with an ancient origin (Figures S1A and S1B; Data S1C).

To investigate whether the recovered human, wolf, and bison DNA derive from single or several individuals from each of these species, we focused on the mitochondrial sequences. Due to their haploid state in single individuals, sequence polymorphisms among mitochondrial reads provide evidence for the presence of multiple individuals (Figure S2; Table 1).[13,21–23] SAT29 displays more than one allele at many known human, wolf, and bison mitochondrial variants, raising the possibility of

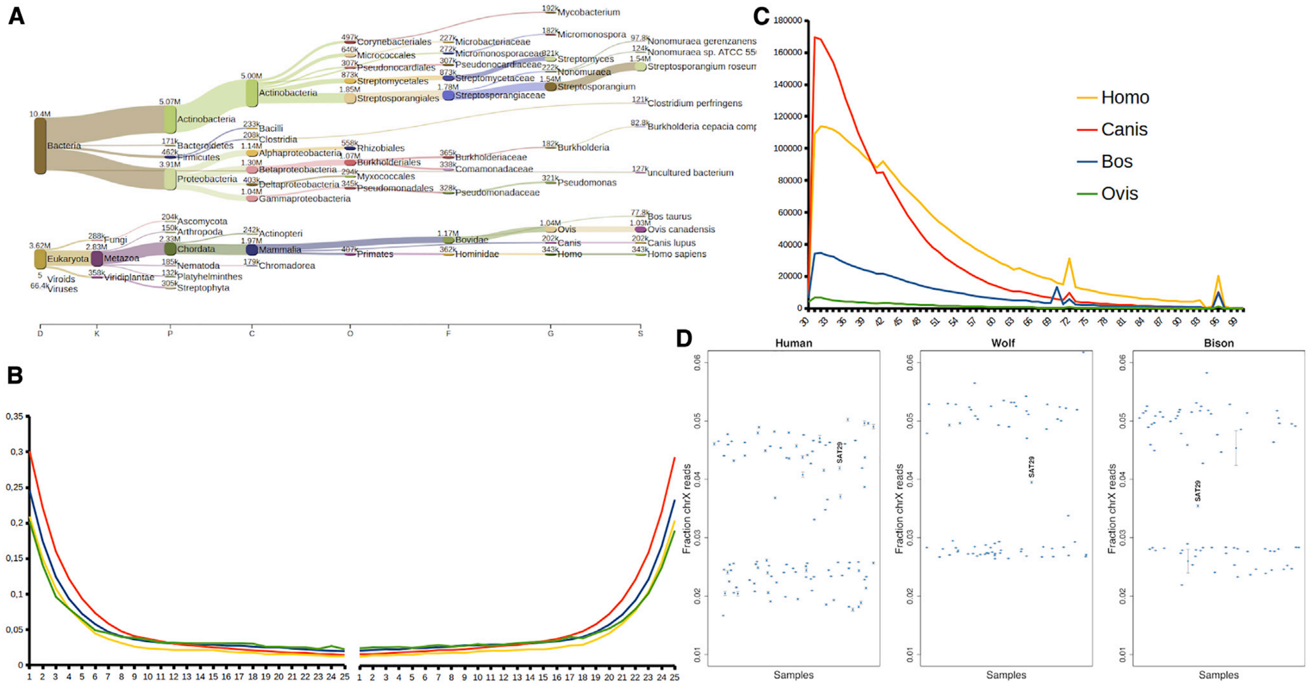

Figure 2. Sequencing data properties

(A) Metagenomic prediction with centrifuge.

(B) Deamination patterns for the filtered reads mapped against the four reference genomes: *Ovis aries*, *Homo sapiens*, *Bos taurus*, and *Canis lupus*.

(C) Fragment length distributions for the four aligned mammalian species.

(D) X chromosome read proportions. For each of the three species, the fraction of nuclear reads mapping to the X chromosome is displayed for the SAT29 sample as well as a number of previously published genomes for comparison. For wolf and bison, the comparative genomes are the modern and ancient genomes used for the ancestry analyses, while for human they are 101 ancient genomes from a study of the Eurasian steppe.[18] Bars denote 95% binomial confidence intervals.[19]

See also Figures S1 and S6 and Data S1.

diversity in the sample (Figure S1C). Applying CALICO,[23] we estimated that 12% of the human mitochondrial reads and 25% of the wolf reads come from minority sources. Schmutzi estimated a lower fraction (1%, 0%–5%) of human minor sources. Finally, contamMix estimated a 4% minority fraction for the human reads and a 31% fraction for the wolf reads. ContamMix estimated no diversity in the bison data, but this could be limited by the low coverage of the mtDNA genome and the low number of potential contaminant mtDNA sequences. Based on the above, we can confirm the presence of DNA from more than a single individual for the wolf data, and likely also the human data, whereas the bison data are inconclusive. However, because of the complexity of the data and the limited coverage, the presented estimates of minority fractions should not be taken as more than rough indications.

Next, we assessed whether modern contamination could explain the detection of mitochondrial polymorphism in the sample (Table 1). Modern sequences are expected to be less fragmented, but we find no difference in the length distribution between deaminated and other reads (method details). Manual inspection of phylogenetically diagnostic positions suggests the presence of minority sources in the human data, including in the deaminated reads (Table S1). However, these variants are present at very low frequencies, in line with the estimated low proportion of minority sources (Tables 1 and S1). All human reads support haplogroup N, suggesting any minority sources would not be from haplogroup M and derivatives. Similar detailed inspection of the wolf data shows that the evidence of substantial polymorphism persists when restricting to deaminated reads (Figure S2B). The bison data are too limited to allow for a similar robust assessment. The diversity signal in the SAT29 sample is thus consistent with DNA deriving from multiple human and wolf individuals, rather than reflecting modern contamination. The contDeam.pl tool from Schmutzi estimates 0% contamination for all three species; in the three files, we have obtained values of 0, which indicates that there is no evidence of contamination based on deamination patterns.[21]

Finally, we examined the sex of the human, canid, and bison genomic data. When comparing the amount of reads mapping to the X chromosome relative to the autosomes, we find that the human data are consistent with deriving from a female individual, or multiple female individuals. In contrast, the wolf and bison X chromosome read fractions are intermediate between those expected for male and female karyotypes, suggesting it may derive from individuals of both sexes, again indicating multiple source individuals (Figure 2D).

Phylogenetic dating of mitochondrial DNA

With the consensus sequence of the human mtDNA reads, we performed a multiple sequence alignment and generated a Bayesian phylogenetic tree with BEAST2 (Figures 3C and S3). The SAT29 sequence is positioned within the diversity of

Table 1. Point estimates and confidence intervals (in parentheses) of minority mitochondrial sequence proportions obtained for the captured mitochondrial data for each of the three analyzed taxa using Schmutzi, ContamMix, and Calico

	Calico	ContamMix	Schmutzi
Homo sapiens	0.12 (0.03–0.21)	0.04 (0.01–0.1)	0.01 (0.0–0.05)
Canis lupus	0.246 (0.21–0.27)	0.31 (0.25–0.39)	–
Bison bonasus	–	0.01	–

See also Figure S2 and Table S1.

haplogroup N, close to the Dzudzuana-3 (25.5 kya) genome from Dzudzuana cave and basal to the modern samples enclosed in haplogroups N, X, and W. Haplogroup N originated outside of Africa from haplogroup L3 between 50 and 60 kya, and is common among present day Near Eastern populations but rare among present-day European populations.[24–26] We then estimated tip dates of the SAT29 human, wolf, and bison consensus mtDNA sequences. The human SAT29 mtDNA consensus has a mean age of 28,543 BP (95% HPD interval, 15,928–41,867 BP), whereas the wolf mtDNA consensus mean age is 28,257 BP (95% HPD interval, 18,083–38,265 BP). The bison mtDNA consensus has an age estimate of 21,928 BP (HPD interval of 14,954–27,987 BP). The phylogenetic positions of the SAT29 consensus sequences were also confirmed in maximum likelihood trees performed with the three datasets (Figure S3). The radiocarbon date of layer BIII of Satsurblia cave is 25.5.–24.2 ka cal. BP, and thus falls within the confidence intervals, and within a few thousand years of the mean estimates, of the mtDNA tip dates for all the three species. Although the mean estimate for the bison is somewhat younger than those for the human and wolves, the 95% confidence intervals overlap. This thus provides strong support for the Pleistocene origin of the recovered DNA.

Ancestry of the SAT29 human nuclear DNA

Using the 661,765 human nuclear reads, we genotyped 11,116 pseudo-haploid positions from the 1240K dataset.[27] To explore the human ancestry of SAT29 within the context of pre- and post-LGM diversity, we performed a principal components analysis (PCA) on 2,335 modern Eurasian genomes and projected 82 ancient individuals onto the resulting components (Figures 3A and S4; Table S2). Previous studies have revealed two different ancient human lineages from the Caucasus that were distinct from the rest of Pleistocene and early Holocene diversity. A late Upper Paleolithic (13.3 kya) genome from Satsurblia cave and a Mesolithic (9.7 kya) genome from the nearby cave of Kotias Klde revealed "Caucasus Hunter Gatherer" (CHG) ancestry, a distinct ancient lineage that split from western hunter-gatherers ~45 ka BP, shortly after the expansion of modern humans into Western Eurasia.[28] A second, older, pre-LGM lineage is represented by genome-wide data from two individuals dated to ~26 ka BP from Dzudzuana cave, southern Caucasus, and likely contributed at least half of the ancestry of later populations in Europe, the Near East, and North Africa.[29] We find that the SAT29 sample clusters with Dzudzuana2 in the PCA and not with the late Upper Paleolithic and Mesolithic genomes from the Caucasus or with any other published pre-LGM Eurasian genomes. Unsupervised ADMIXTURE clustering[30] further supports the similarity between SAT29 and Dzudzuana2 (Figures 3B and S4; Tables S2 and S3).

We used outgroup f_3-statistics to quantify the amount of shared genetic drift between SAT29 and other ancient genomes.[31] SAT29 shares more drift with Villabruna (Italy, 12,140 ± 70 bp)[32] and Dzudzuana2 than with other ancient individuals (Figure S4B), including the post-LGM individuals from the Caucasus (Satsurblia and Kotias). Among present-day Eurasian populations, SAT29 shows higher genetic affinity to Northern and Western Europeans rather than Central and South Asians (Figure S4C). Our results for the SAT29 human autosomal data are thus consistent with the results reported by Lazaridis et al.,[29] revealing a previously undocumented pre-LGM human ancestry from the Caucasus that contributed to various later Eurasian populations. The low coverage of the SAT29 environmental genome, however, did not allow us to further analyze possible differences in the ancestry between Dzuzuana2 and SAT29.

Next, we investigated if the amount of Neanderthal ancestry in the SAT29 human environmental genome could be estimated. Using an f_4-ratio,[31] we estimated 1% Neanderthal ancestry, with confidence intervals of 0%–6.6%. The point estimate is similar to that of Dzudzuana2 and likely lower than that of Paleolithic European and present-day West Eurasian populations due to dilution from large amounts of Basal Eurasian ancestry.[29] However, the large uncertainty of the estimate precludes any strong conclusions. Our results thus suggest that, unless substantially larger amounts of autosomal DNA can be recovered than what is analyzed here, sediment DNA is unlikely to allow for confident estimates of archaic ancestry proportions.

Ancestry of the SAT29 wolf nuclear DNA

We analyzed the 2,378,237 *Canis* reads using a set of variants among 722 modern wolves, dogs, and other canid species.[33] We randomly sampled one SAT29 read at each position, resulting in a genotype call at 439,426 transversion variants. In ADMIXTURE analyses (Figures 4A and S5) the SAT29 sample clusters with Eurasian wolves, and using f_4-statistics, we found that it clearly shares genetic drift with wolves and dogs, to the exclusion of coyotes, golden jackals, and other canids (Z >30 for all species, Method details). It does not, however, display stronger affinity to wolves over dogs or vice versa (Figure S5).

We next used admixture graphs to further investigate the relationship of the SAT29 environmental genome to present-day wolves and dogs, as well as two Pleistocene wolf genomes from Siberia (35–33 ka), which have ancestries that are basal to modern wolves and dogs.[34,35] We tested all possible topologies without admixture relating a coyote, SAT29, a modern wolf, a modern dog, and the two Pleistocene Siberian wolves, while explicitly accounting for reference bias in the ancient genomes (Method details). Only three of the 100 graphs provide good fits and feature the Siberian Pleistocene wolves on a branch basal to modern populations. The graphs fit equally well and differ only in that SAT29 is placed either basal to the Siberian Pleistocene branch, on this branch, or downstream of this branch (Figure 4B). Previous studies have found that present-day wolf population structure has mostly formed after the LGM.[23,34,36–39] Our results are consistent with this scenario because the SAT29 environmental genome harbored an

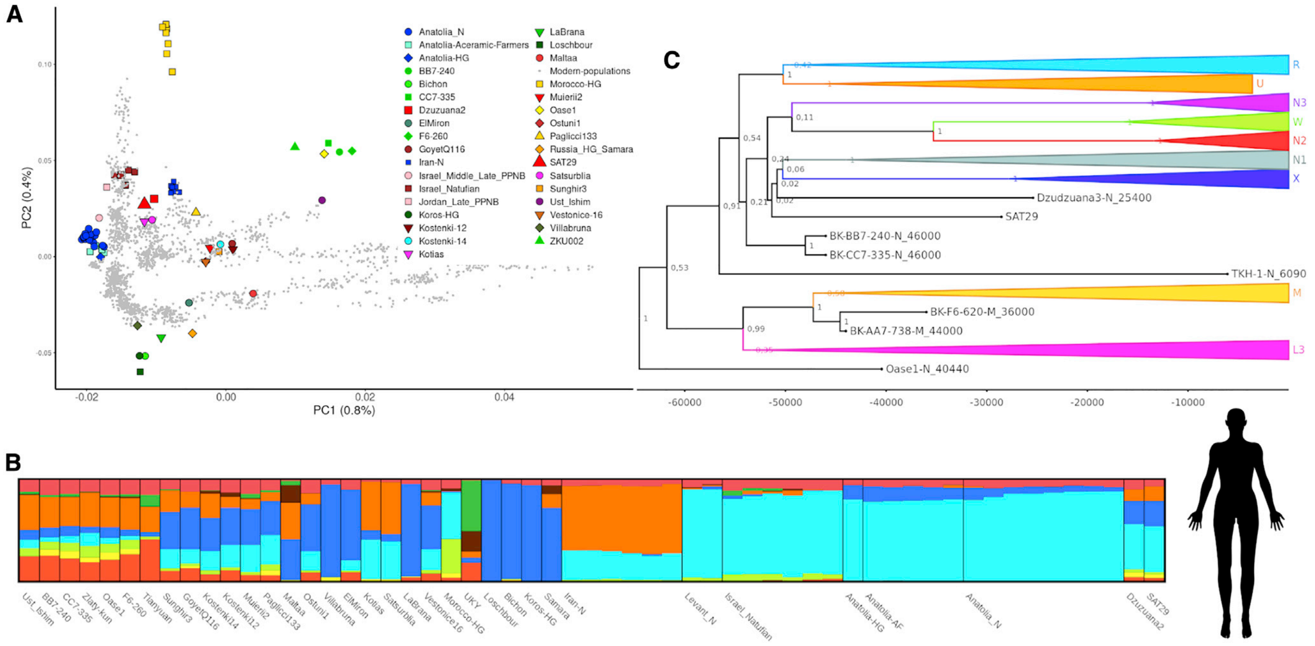

Figure 3. SAT29 human genomics

(A) A principal component analysis built with 2,335 modern Eurasian samples into which 82 ancient samples were projected. SAT29 appears closest to Dzudzuana2.

(B) In ADMIXTURE clustering with 82 ancient genomes, SAT29 displays a profile similar to that of Dzudzuana2.

(C) Bayesian tree built with 69 ancient genomes and 167 modern mitochondrial genomes. SAT29 falls close to the Dzudzuana2 genome and within the N haplogroup diversity. The values in the tree nodes represent the posterior probabilities.

See also Figures S3 and S4 and Tables S2 and S3.

ancestry that diverged from the ancestors of modern wolves and dogs before these diversified. Although Late Pleistocene wolves in the Caucasus were not closely related to those in Siberia, they thus had a similarly basal ancestry that has either gone extinct or been transformed by later population processes.

These autosomal ancestry results are consistent with the mitochondrial capture results, in which the SAT29 wolf consensus sequence falls on a branch together with two ancient wolves from the Aghitu-3 cave in Armenia (Figures 4C and S3; Table S4), dated to 31–30 ka, on a seemingly extinct West Eurasian branch of the wolf mtDNA phylogeny.[37] To further characterize the phylogenetic identity of the SAT29 consensus sequence, we recovered mtDNA from two wolf bones from Satsurblia cave through capture: a 4.9-fold genome from sample Y7d from layer B IVa (25.5–24.4 ka cal BP) and a 5.2-fold genome from sample T20a from layer A IIb (17.9–16.2 ka cal BP). These sequences fall close to SAT29 in the phylogeny, supporting the endogenous origin of the SAT29 environmental genome and demonstrating mitochondrial genetic continuity of wolf populations in the Caucasus for at least 10,000 years through the end of the LGM. When adding these sequences to the phylogenetic tip dating, the SAT29 age estimate comes out slightly younger (mean of 19,937). However, it is possible that the low quality of these two sequences hurts the accuracy of the inference in some way.

Ancestry of the SAT29 bison nuclear DNA

We compiled a number of Bovine genomes,[40–42] identified 1.4 million heterozygous transversion sites in a gaur genome, and assigned genotypes to all individuals at these sites by randomly sampling one read per position. This resulted in a genotype call at 27,724 transversion variants for the SAT29 bovid sample. Using ADMIXTURE clustering (Figure 5A) and f_4-statistics, we find that the SAT29 environmental genome shares genetic drift with bison (*Bison sp.*), to the exclusion of aurochs, domestic cattle, and Asian bovid species (Z >20 for all species, Method details). This is thus consistent with the mitochondrial sequence being of *Bison sp.* origin. It also provides important authentication of the soil metagenomic approach, because the identified environmental genome is a different species from the cattle (*Bos taurus*) used as a reference genome.

We next used f_4-statistics and admixture graphs to further investigate the relationship of the SAT29 environmental bison genome to present-day populations as well as early 20th century European bison (*Bison bonasus;* wisents) from Poland and the Caucasus. SAT29 is closer to these historical European individuals, as well as to modern European bison, than to a North American bison ($|Z| > 6$), implying that the divergence between European and American populations predates the age of SAT29. The Caucasian and the Polish populations have been classified as separate subspecies of European bison, but the SAT29 sample is not detectably closer to one over the other. Furthermore, these two recent populations share genetic drift to the exclusion of the SAT29 sample ($|Z| = 3.4$ and 4.5 for the two relevant f_4-statistics).

We tested all possible topologies without admixture to summarize the relationships between these bison genomes. The

CellPress
OPEN ACCESS

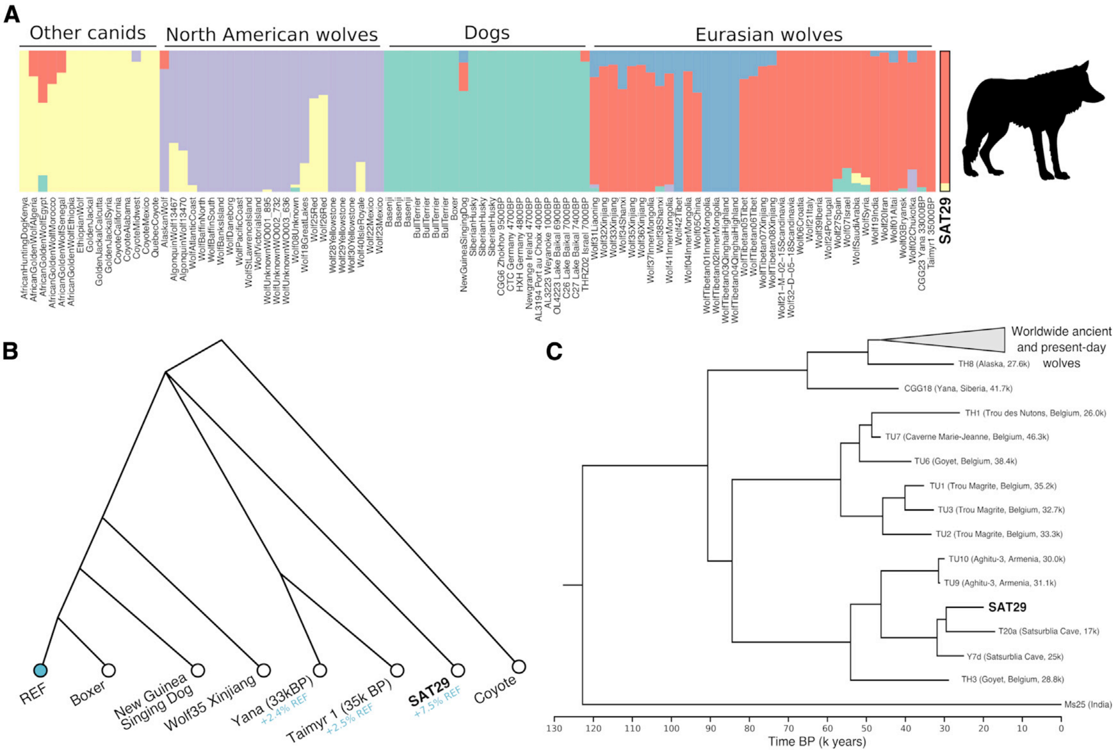

Figure 4. SAT29 wolf genomics

(A) In ADMIXTURE clustering with wolves, dogs, and other canid species, the SAT29 sample clusters with Eurasian wolves.

(B) Population history model relating the SAT29 sample to modern wolves, dogs, and Pleistocene Siberian wolves. Inferred admixture proportions from the dog reference genome (REF) to account for reference bias, are shown in blue. The trifurcation point indicates that it cannot confidently be determined on which side of this point the SAT29 sample falls.

(C) Bayesian tree of 68 modern and 39 ancient wolf mitochondrial genomes.

See also Figures S3 and S5 and Table S4.

best-fitting topology has SAT29 as basal to the historical genomes from Poland and the Caucasus, and the American bison as basal to all of these (Figure 5B). This model explains all f_4-statistics except a signal of some excess affinity between the American and Polish genomes. We do not attempt to discriminate between more complex models involving possible admixture events. Overall, the results tentatively suggest that the history of bison in western Eurasia might share some features with wolf history, in that Late Pleistocene ancestries appear basal to present-day populations, suggesting that population structure has been substantially reshaped since the LGM. This autosomal ancestry is also consistent with the mitochondrial phylogeny, in which the SAT29 bison consensus sequence falls on a branch with other ancient west Eurasian bison within a clade that has been called "Bb2,"[43] closest to the sequence of a Holocene Armenian individual (Figures 5C and S3; Table S5).[42]

We additionally recovered 72,100 reads aligning to *Ovis aries* and explored these reads with a dataset of 103 *Ovis* and *Capra* genomes from 10 species. Tests of the form f_4(Oreamnos,-SAT29;C,D) indicated that the SAT29 sample is more similar to *Ovis* than to *Capra*, but it does not cluster with any of present-day *Ovis* species (Figure S6). We were thus unable to elaborate on the ancestry of this *Ovis* DNA.

DISCUSSION

Our results demonstrate that unbiased shotgun sequencing of sediment ancient DNA can yield genome-wide data that is informative about the ancestry of several taxa. The DNA retrieved here is lower in quantity, and hence resolution, compared to what is often obtained from various well-preserved bones and teeth. Nonetheless, it provided information largely comparable to low-coverage ancient genome sequences and allowed us to apply complementary analyses of multiple mammalian species to reconstruct some aspects of their population histories. Our results thus point to new possibilities in the study of sediment ancient DNA, demonstrating that it can serve as an additional or alternative source of genome-wide information to skeletal remains.

The damage characteristics, the mitochondrial tip dates, and the fact that all three environmental genomes represent ancestries that no longer exist among the given species strongly support a Pleistocene origin of the recovered DNA. Moreover, the deamination patterns and fragment length distributions are similar for the human, wolf, and bison DNA. Additionally, the results of the population genetics analyses are in accordance with those published by other studies on skeletal genomes from the same region and time period.[29] These observations thus suggest

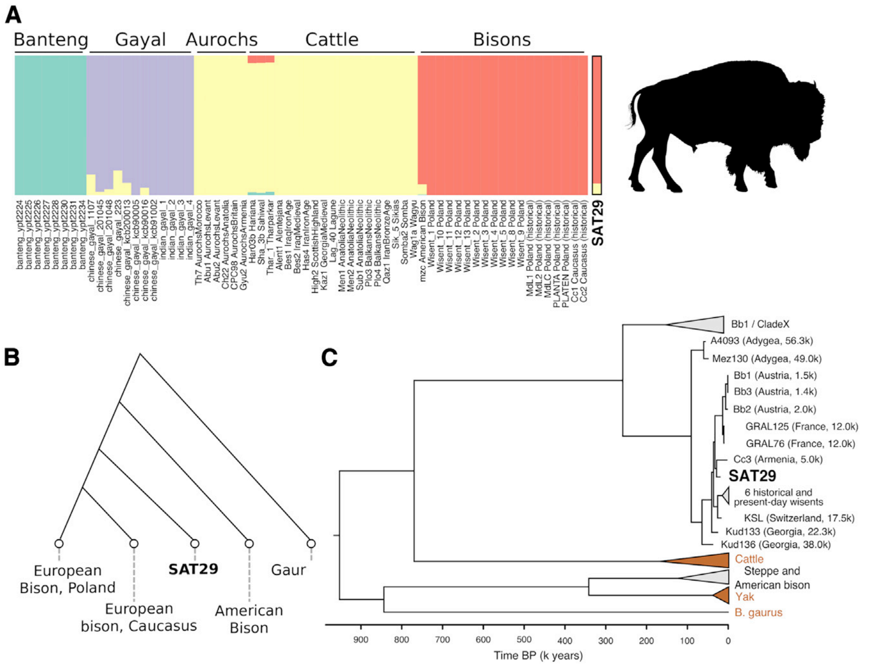

Figure 5. SAT29 bison genomics

(A) In ADMIXTURE clustering with cattle, aurochs, bison, gayals, and bantengs, the SAT29 sample clusters with bison.

(B) A population history model relating the SAT29 sample to present-day American and historical (early 20th century) European bison from Poland and the Caucasus.

(C) Bayesian tree built with SAT29 and other 70 bovid mitochondrial genomes.

See also Figure S3 and Table S5.

that the DNA has not been significantly affected by modern contamination or leaching through archaeological layers.

Although promising, the study of whole genomes from archeological sediments also has several limitations. We report evidence for the presence of sequences deriving from multiple individuals in our environmental genomes. The specific nature of environmental genomes, which in most cases likely compose a mosaic of several individuals from a given taxon, complicates certain analyses, in particular the reconstruction of mitochondrial consensus sequences. On the other hand, allele frequency-based analyses of genome-wide relationships are well-suited to sequences deriving from multiple individuals, as long as these are of largely the same ancestry. Despite these limitations, genome-wide ancient sediment DNA might open new directions for the study of whole ecosystems, including interactions between different species and aspects of human practices linked to the use of animals or plants.

STAR★METHODS

Detailed methods are provided in the online version of this paper and include the following:

- KEY RESOURCES TABLE
- RESOURCE AVAILABILITY
  - Lead contact
  - Materials availability
  - Data and code availability
- EXPERIMENTAL MODEL AND SUBJECT DETAILS
  - Archeological context
- METHOD DETAILS
  - DNA extraction
  - PCR, quality control and next generation sequencing
  - Mitochondrial capture
  - Human DNA screening
  - Bioinformatic processing of sample SAT29
  - Damage pattern analysis
  - BLASTN analysis
  - Human population genetics
  - Sex determination of SAT29 human reads
  - Neanderthal ancestry in SAT29
  - Human mitochondrial analysis
  - mtDNA mixture estimate
  - Presence of multiple individuals identification
  - mtDNA consensus calling
  - Human mitochondrial tip dating
  - Wolf genome: comparative dataset
  - Wolf genome: ancestry analyses
  - Canid bone testing
  - Canid mitochondrial tip dating
  - Presence of multiple individuals
  - Bison genome: comparative dataset
  - Bison genome: ancestry analyses
  - Bison mitochondria capture and analysis
  - Ovis genomic analysis

SUPPLEMENTAL INFORMATION

Supplemental information can be found online at https://doi.org/10.1016/j.cub.2021.06.023.

ACKNOWLEDGMENTS

This study makes use of data generated by the NextGen Consortium. We acknowledge Gabriel Renaud for advice on Schmutzi and on the analysis of the mitochondrial genome. We acknowledge Spencer Sawyer, Manuela Alscher, and Odin for modern DNA. We acknowledge David Reich and Iosif Lazaridis for sharing the data of the Dzudzuana2 genome and helping us with its analysis. This work has been supported by the "Mineralogical Preservation of the Human Biome from the Depth of Time" (MINERVA) research platform, code AGB326800, from the University of Vienna. P.S. was supported by the European Research Council (852558), a Wellcome Trust Investigator

award (217223/Z/19/Z), and the Vallee Foundation. P.S. and A.B. were supported by Francis Crick Institute core funding (FC001595) from Cancer Research UK, the UK Medical Research Council, and the Wellcome Trust. This research was funded in whole, or in part, by the Wellcome Trust (FC001595). For the purpose of open access, the author has applied a CC BY public copyright license to any author accepted manuscript version arising from this submission T.C.C. was supported by the Medical Trainee PhD Scholarship, UCD.

AUTHOR CONTRIBUTIONS

R.P., S.S., and P.G. conceived the study. A.B.-C., D.L., T.M., N.J., Z.M., and G.B.-O. provided samples. M.C.S. collected the samples. S.S., O.C., D.F., T.C.C., V.O., K.T.Ö., and R.N.M.F. performed the experimental work. P.G., S.S., A.B., A.M., and T.C.C. analyzed the data. P.G., S.S., A.B., R.P., and P.S. wrote the manuscript with inputs from all coauthors.

DECLARATION OF INTERESTS

The authors declare no competing interests.

Received: January 6, 2021
Revised: April 23, 2021
Accepted: June 9, 2021
Published: July 12, 2021

REFERENCES

1. Hagelberg, E., Sykes, B., and Hedges, R. (1989). Ancient bone DNA amplified. Nature *342*, 485.
2. Höss, M., Dilling, A., Currant, A., and Pääbo, S. (1996). Molecular phylogeny of the extinct ground sloth Mylodon darwinii. Proc. Natl. Acad. Sci. USA *93*, 181–185.
3. Gilbert, M.T.P., Tomsho, L.P., Rendulic, S., Packard, M., Drautz, D.I., Sher, A., Tikhonov, A., Dalén, L., Kuznetsova, T., Kosintsev, P., et al. (2007). Whole-genome shotgun sequencing of mitochondria from ancient hair shafts. Science *317*, 1927–1930.
4. Racimo, F., Sikora, M., Vander Linden, M., Schroeder, H., and Lalueza-Fox, C. (2020). Beyond broad strokes: sociocultural insights from the study of ancient genomes. Nat. Rev. Genet. *21*, 355–366.
5. Hofreiter, M., Serre, D., Poinar, H.N., Kuch, M., and Pääbo, S. (2001). Ancient DNA. Nat. Rev. Genet. *2*, 353–359.
6. Willerslev, E., Hansen, A.J., Binladen, J., Brand, T.B., Gilbert, M.T.P., Shapiro, B., Bunce, M., Wiuf, C., Gilichinsky, D.A., and Cooper, A. (2003). Diverse plant and animal genetic records from Holocene and Pleistocene sediments. Science *300*, 791–795.
7. Hofreiter, M., Mead, J.I., Martin, P., and Poinar, H.N. (2003). Molecular caving. Curr. Biol. *13*, R693–R695.
8. Pedersen, M.W., Ruter, A., Schweger, C., Friebe, H., Staff, R.A., Kjeldsen, K.K., Mendoza, M.L.Z., Beaudoin, A.B., Zutter, C., Larsen, N.K., et al. (2016). Postglacial viability and colonization in North America's ice-free corridor. Nature *537*, 45–49.
9. Søe, M.J., Nejsum, P., Seersholm, F.V., Fredensborg, B.L., Habraken, R., Haase, K., Hald, M.M., Simonsen, R., Højlund, F., Blanke, L., et al. (2018). Ancient DNA from latrines in Northern Europe and the Middle East (500 BC-1700 AD) reveals past parasites and diet. PLoS ONE *13*, e0195481.
10. Willerslev, E., Davison, J., Moora, M., Zobel, M., Coissac, E., Edwards, M.E., Lorenzen, E.D., Vestergård, M., Gussarova, G., Haile, J., et al. (2014). Fifty thousand years of Arctic vegetation and megafaunal diet. Nature *506*, 47–51.
11. Graham, R.W., Belmecheri, S., Choy, K., Culleton, B.J., Davies, L.J., Froese, D., Heintzman, P.D., Hritz, C., Kapp, J.D., Newsom, L.A., et al. (2016). Timing and causes of mid-Holocene mammoth extinction on St. Paul Island, Alaska. Proc. Natl. Acad. Sci. USA *113*, 9310–9314.
12. Pedersen, M.W., Overballe-Petersen, S., Ermini, L., Sarkissian, C.D., Haile, J., Hellstrom, M., Spens, J., Thomsen, P.F., Bohmann, K., Cappellini, E., et al. (2015). Ancient and modern environmental DNA. Philos. Trans. R. Soc. Lond. B Biol. Sci. *370*, 20130383.
13. Slon, V., Hopfe, C., Weiß, C.L., Mafessoni, F., de la Rasilla, M., Lalueza-Fox, C., Rosas, A., Soressi, M., Knul, M.V., Miller, R., et al. (2017). Neandertal and Denisovan DNA from Pleistocene sediments. Science *356*, 605–608.
14. Zhang, D., Xia, H., Chen, F., Li, B., Slon, V., Cheng, T., Yang, R., Jacobs, Z., Dai, Q., Massilani, D., et al. (2020). Denisovan DNA in Late Pleistocene sediments from Baishiya Karst Cave on the Tibetan Plateau. Science *370*, 584–587.
15. Vernot, B., Zavala, E.I., Gómez-Olivencia, A., Jacobs, Z., Slon, V., Mafessoni, F., Romagné, F., Pearson, A., Petr, M., Sala, N., et al. (2021). Unearthing Neanderthal population history using nuclear and mitochondrial DNA from cave sediments. Science *372*, eabf1667.
16. Pinhasi, R., Meshveliani, T., Matskevich, Z., Bar-Oz, G., Weissbrod, L., Miller, C.E., Wilkinson, K., Lordkipanidze, D., Jakeli, N., Kvavadze, E., et al. (2014). Satsurblia: new insights of human response and survival across the Last Glacial Maximum in the southern Caucasus. PLoS ONE *9*, e111271.
17. Kim, D., Song, L., Breitwieser, F.P., and Salzberg, S.L. (2016). Centrifuge: rapid and sensitive classification of metagenomic sequences. Genome Res. *26*, 1721–1729.
18. Allentoft, M.E., Sikora, M., Sjögren, K.-G., Rasmussen, S., Rasmussen, M., Stenderup, J., Damgaard, P.B., Schroeder, H., Ahlström, T., Vinner, L., et al. (2015). Population genomics of Bronze Age Eurasia. Nature *522*, 167–172.
19. Skoglund, P., Storå, J., Götherström, A., and Jakobsson, M. (2013). Accurate sex identification of ancient human remains using DNA shotgun sequencing. J. Archaeol. Sci. *40*, 4477–4482.
20. Maricic, T., Whitten, M., and Pääbo, S. (2010). Multiplexed DNA sequence capture of mitochondrial genomes using PCR products. PLoS ONE *5*, e14004.
21. Renaud, G., Slon, V., Duggan, A.T., and Kelso, J. (2015). Schmutzi: estimation of contamination and endogenous mitochondrial consensus calling for ancient DNA. Genome Biol. *16*, 224.
22. Meyer, M., Fu, Q., Aximu-Petri, A., Glocke, I., Nickel, B., Arsuaga, J.-L., Martínez, I., Gracia, A., de Castro, J.M.B., Carbonell, E., and Pääbo, S. (2014). A mitochondrial genome sequence of a hominin from Sima de los Huesos. Nature *505*, 403–406.
23. Bergström, A., Frantz, L., Schmidt, R., Ersmark, E., Lebrasseur, O., Girdland-Flink, L., Lin, A.T., Storå, J., Sjögren, K.-G., Anthony, D., et al. (2020). Origins and genetic legacy of prehistoric dogs. Science *370*, 557–564.
24. Soares, P., Alshamali, F., Pereira, J.B., Fernandes, V., Silva, N.M., Afonso, C., Costa, M.D., Musilová, E., Macaulay, V., Richards, M.B., et al. (2012). The Expansion of mtDNA Haplogroup L3 within and out of Africa. Mol. Biol. Evol. *29*, 915–927.
25. Fernandes, V., Alshamali, F., Alves, M., Costa, M.D., Pereira, J.B., Silva, N.M., Cherni, L., Harich, N., Cerny, V., Soares, P., et al. (2012). The Arabian cradle: mitochondrial relicts of the first steps along the southern route out of Africa. Am. J. Hum. Genet. *90*, 347–355.
26. Drake, N.A., Blench, R.M., Armitage, S.J., Bristow, C.S., and White, K.H. (2011). Ancient watercourses and biogeography of the Sahara explain the peopling of the desert. Proc. Natl. Acad. Sci. USA *108*, 458–462.
27. Haak, W., Lazaridis, I., Patterson, N., Rohland, N., Mallick, S., Llamas, B., Brandt, G., Nordenfelt, S., Harney, E., Stewardson, K., et al. (2015). Massive migration from the steppe was a source for Indo-European languages in Europe. Nature *522*, 207–211.
28. Jones, E.R., Gonzalez-Fortes, G., Connell, S., Siska, V., Eriksson, A., Martiniano, R., McLaughlin, R.L., Gallego Llorente, M., Cassidy, L.M., Gamba, C., et al. (2015). Upper Palaeolithic genomes reveal deep roots of modern Eurasians. Nat. Commun. *6*, 8912.
29. Lazaridis, I., Belfer-Cohen, A., Mallick, S., Patterson, N., Cheronet, O., Rohland, N., Bar-Oz, G., Bar-Yosef, O., Jakeli, N., Kvavadze, E., et al.

(2018). Paleolithic DNA from the Caucasus reveals core of West Eurasian ancestry. bioRxiv, 423079.

30. Alexander, D.H., and Lange, K. (2011). Enhancements to the ADMIXTURE algorithm for individual ancestry estimation. BMC Bioinformatics *12*, 246.

31. Patterson, N., Moorjani, P., Luo, Y., Mallick, S., Rohland, N., Zhan, Y., Genschoreck, T., Webster, T., and Reich, D. (2012). Ancient admixture in human history. Genetics *192*, 1065–1093.

32. Fu, Q., Posth, C., Hajdinjak, M., Petr, M., Mallick, S., Fernandes, D., Furtwängler, A., Haak, W., Meyer, M., Mittnik, A., et al. (2016). The genetic history of Ice Age Europe. Nature *534*, 200–205.

33. Plassais, J., Kim, J., Davis, B.W., Karyadi, D.M., Hogan, A.N., Harris, A.C., Decker, B., Parker, H.G., and Ostrander, E.A. (2019). Whole genome sequencing of canids reveals genomic regions under selection and variants influencing morphology. Nat. Commun. *10*, 1489.

34. Skoglund, P., Ersmark, E., Palkopoulou, E., and Dalén, L. (2015). Ancient wolf genome reveals an early divergence of domestic dog ancestors and admixture into high-latitude breeds. Curr. Biol. *25*, 1515–1519.

35. Sinding, M.S., Gopalakrishnan, S., Ramos-Madrigal, J., de Manuel, M., Pitulko, V.V., Kuderna, L., Feuerborn, T.R., Frantz, L.A.F., Vieira, F.G., Niemann, J., et al. (2020). Arctic-adapted dogs emerged at the Pleistocene-Holocene transition. Science *368*, 1495–1499.

36. Freedman, A.H., Gronau, I., Schweizer, R.M., Ortega-Del Vecchyo, D., Han, E., Silva, P.M., Galaverni, M., Fan, Z., Marx, P., Lorente-Galdos, B., et al. (2014). Genome sequencing highlights the dynamic early history of dogs. PLoS Genet. *10*, e1004016.

37. Loog, L., Thalmann, O., Sinding, M.S., Schuenemann, V.J., Perri, A., Germonpré, M., Bocherens, H., Witt, K.E., Samaniego Castruita, J.A., Velasco, M.S., et al. (2020). Ancient DNA suggests modern wolves trace their origin to a Late Pleistocene expansion from Beringia. Mol. Ecol. *29*, 1596–1610.

38. Ramos-Madrigal, J., Sinding, M.-H.S., Carøe, C., Mak, S.S.T., Niemann, J., Samaniego Castruita, J.A., Fedorov, S., Kandyba, A., Germonpré, M., Bocherens, H., et al. (2021). Genomes of Pleistocene Siberian Wolves Uncover Multiple Extinct Wolf Lineages. Curr. Biol. *31*, 198–206.e8.

39. Fan, Z., Silva, P., Gronau, I., Wang, S., Armero, A.S., Schweizer, R.M., Ramirez, O., Pollinger, J., Galaverni, M., Ortega Del-Vecchyo, D., et al. (2016). Worldwide patterns of genomic variation and admixture in gray wolves. Genome Res. *26*, 163–173.

40. Wu, D.-D., Ding, X.-D., Wang, S., Wójcik, J.M., Zhang, Y., Tokarska, M., Li, Y., Wang, M.-S., Faruque, O., Nielsen, R., et al. (2018). Pervasive introgression facilitated domestication and adaptation in the Bos species complex. Nat. Ecol. Evol. *2*, 1139–1145.

41. Verdugo, M.P., Mullin, V.E., Scheu, A., Mattiangeli, V., Daly, K.G., Maisano Delser, P., Hare, A.J., Burger, J., Collins, M.J., Kehati, R., et al. (2019). Ancient cattle genomics, origins, and rapid turnover in the Fertile Crescent. Science *365*, 173–176.

42. Węcek, K., Hartmann, S., Paijmans, J.L.A., Taron, U., Xenikoudakis, G., Cahill, J.A., Heintzman, P.D., Shapiro, B., Baryshnikov, G., Bunevich, A.N., et al. (2017). Complex Admixture Preceded and Followed the Extinction of Wisent in the Wild. Mol. Biol. Evol. *34*, 598–612.

43. Massilani, D., Guimaraes, S., Brugal, J.-P., Bennett, E.A., Tokarska, M., Arbogast, R.-M., Baryshnikov, G., Boeskorov, G., Castel, J.-C., Davydov, S., et al. (2016). Past climate changes, population dynamics and the origin of Bison in Europe. BMC Biol. *14*, 93.

44. Martin, M. (2011). Cutadapt removes adapter sequences from high-throughput sequencing reads. EMBnet. J. *17*, 10–12.

45. Hannon, G.J. (2010). FASTX-Toolkit.

46. Simpson, J.T., and Durbin, R. (2010). Efficient construction of an assembly string graph using the FM-index. Bioinformatics *26*, i367–i373.

47. Breitwieser, F.P., and Salzberg, S.L. (2020). Pavian: interactive analysis of metagenomics data for microbiome studies and pathogen identification. Bioinformatics *36*, 1303–1304.

48. Li, H., and Durbin, R. (2009). Fast and accurate short read alignment with Burrows-Wheeler transform. Bioinformatics *25*, 1754–1760.

49. Quinlan, A.R., and Hall, I.M. (2010). BEDTools: a flexible suite of utilities for comparing genomic features. Bioinformatics *26*, 841–842.

50. Okonechnikov, K., Conesa, A., and García-Alcalde, F. (2016). Qualimap 2: advanced multi-sample quality control for high-throughput sequencing data. Bioinformatics *32*, 292–294.

51. Jónsson, H., Ginolhac, A., Schubert, M., Johnson, P.L.F., and Orlando, L. (2013). mapDamage2.0: fast approximate Bayesian estimates of ancient DNA damage parameters. Bioinformatics *29*, 1682–1684.

52. Schiffels, S., Haak, W., Paajanen, P., Llamas, B., Popescu, E., Loe, L., Clarke, R., Lyons, A., Mortimer, R., Sayer, D., et al. (2016). Iron Age and Anglo-Saxon genomes from East England reveal British migration history. Nat. Commun. *7*, 10408.

53. Patterson, N., Price, A.L., and Reich, D. (2006). Population structure and eigenanalysis. PLoS Genet. *2*, e190.

54. Alexander, D.H., Novembre, J., and Lange, K. (2009). Fast model-based estimation of ancestry in unrelated individuals. Genome Res. *19*, 1655–1664.

55. Purcell, S., Neale, B., Todd-Brown, K., Thomas, L., Ferreira, M.A.R., Bender, D., Maller, J., Sklar, P., de Bakker, P.I.W., Daly, M.J., and Sham, P.C. (2007). PLINK: a tool set for whole-genome association and population-based linkage analyses. Am. J. Hum. Genet. *81*, 559–575.

56. Behr, A.A., Liu, K.Z., Liu-Fang, G., Nakka, P., and Ramachandran, S. (2016). pong: fast analysis and visualization of latent clusters in population genetic data. Bioinformatics *32*, 2817–2823.

57. Huson, D.H., Auch, A.F., Qi, J., and Schuster, S.C. (2007). MEGAN analysis of metagenomic data. Genome Res. *17*, 377–386.

58. Camacho, C., Coulouris, G., Avagyan, V., Ma, N., Papadopoulos, J., Bealer, K., and Madden, T.L. (2009). BLAST+: architecture and applications. BMC Bioinformatics *10*, 421.

59. Jun, G., Wing, M.K., Abecasis, G.R., and Kang, H.M. (2015). An efficient and scalable analysis framework for variant extraction and refinement from population-scale DNA sequence data. Genome Res. *25*, 918–925.

60. Weissensteiner, H., Pacher, D., Kloss-Brandstätter, A., Forer, L., Specht, G., Bandelt, H.-J., Kronenberg, F., Salas, A., and Schönherr, S. (2016). HaploGrep 2: mitochondrial haplogroup classification in the era of high-throughput sequencing. Nucleic Acids Res. *44* (W1), W58-63.

61. Suchard, M.A., Lemey, P., Baele, G., Ayres, D.L., Drummond, A.J., and Rambaut, A. (2018). Bayesian phylogenetic and phylodynamic data integration using BEAST 1.10. Virus Evol. *4*, vey016.

62. McKenna, A., Hanna, M., Banks, E., Sivachenko, A., Cibulskis, K., Kernytsky, A., Garimella, K., Altshuler, D., Gabriel, S., Daly, M., and DePristo, M.A. (2010). The Genome Analysis Toolkit: a MapReduce framework for analyzing next-generation DNA sequencing data. Genome Res. *20*, 1297–1303.

63. Kalandadze, A.N., and Kalandadze, K.S. (1978). Archaeological Research of Karstic Caves in Tskaltubo region (in Georgian, with Russian summary). Caves of Georgia, 116–136.

64. Collin, T.C. (2019). Development and Application of a Metagenomic Ancient DNA Approach for the Identification and Assessment of Taxa from Anthropogenic Sediments: Reconstructing the Past. PhD thesis (University College Dublin).

65. Dabney, J., Knapp, M., Glocke, I., Gansauge, M.-T., Weihmann, A., Nickel, B., Valdiosera, C., García, N., Pääbo, S., Arsuaga, J.-L., and Meyer, M. (2013). Complete mitochondrial genome sequence of a Middle Pleistocene cave bear reconstructed from ultrashort DNA fragments. Proc. Natl. Acad. Sci. USA *110*, 15758–15763.

66. Meyer, M., and Kircher, M. (2010). Illumina sequencing library preparation for highly multiplexed target capture and sequencing. Cold Spring Harb. Protoc. *2010*, db.prot5448.

67. Gamba, C., Jones, E.R., Teasdale, M.D., McLaughlin, R.L., Gonzalez-Fortes, G., Mattiangeli, V., Domboróczki, L., Kővári, I., Pap, I., Anders,

CellPress
OPEN ACCESS

A., et al. (2014). Genome flux and stasis in a five millennium transect of European prehistory. Nat. Commun. *5*, 5257.

68. Gunnarsdóttir, E.D., Li, M., Bauchet, M., Finstermeier, K., and Stoneking, M. (2011). High-throughput sequencing of complete human mtDNA genomes from the Philippines. Genome Res. *21*, 1–11.

69. Sawyer, S., Krause, J., Guschanski, K., Savolainen, V., and Pääbo, S. (2012). Temporal patterns of nucleotide misincorporations and DNA fragmentation in ancient DNA. PLoS ONE *7*, e34131.

70. Thalmann, O., Shapiro, B., Cui, P., Schuenemann, V.J., Sawyer, S.K., Greenfield, D.L., Germonpré, M.B., Sablin, M.V., López-Giráldez, F., Domingo-Roura, X., et al. (2013). Complete mitochondrial genomes of ancient canids suggest a European origin of domestic dogs. Science *342*, 871–874.

71. Feuerborn, T.R., Palkopoulou, E., van der Valk, T., von Seth, J., Munters, A., Pečnerová, P., Dehasque, M., Ureña, I., Ersmark, E., Lagerholm, V.K., et al. (2020). Competitive mapping allows to identify and exclude human DNA contamination in ancient faunal genomic datasets. bioRxiv. https://doi.org/10.1186/s12864-020-07229-y.

72. Li, H., Handsaker, B., Wysoker, A., Fennell, T., Ruan, J., Homer, N., Marth, G., Abecasis, G., and Durbin, R.; 1000 Genome Project Data Processing Subgroup (2009). The Sequence Alignment/Map format and SAMtools. Bioinformatics *25*, 2078–2079.

73. Andrews, S. (2010). A Quality Control Tool for High Throughput Sequence Data. https://www.bioinformatics.babraham.ac.uk/projects/fastqc/.

74. Altschul, S.F., Gish, W., Miller, W., Myers, E.W., and Lipman, D.J. (1990). Basic local alignment search tool. J. Mol. Biol. *215*, 403–410.

75. Lazaridis, I., Nadel, D., Rollefson, G., Merrett, D.C., Rohland, N., Mallick, S., Fernandes, D., Novak, M., Gamarra, B., Sirak, K., et al. (2016). Genomic insights into the origin of farming in the ancient Near East. Nature *536*, 419–424.

76. Sikora, M., Seguin-Orlando, A., Sousa, V.C., Albrechtsen, A., Korneliussen, T., Ko, A., Rasmussen, S., Dupanloup, I., Nigst, P.R., Bosch, M.D., et al. (2017). Ancient genomes show social and reproductive behavior of early Upper Paleolithic foragers. Science *358*, 659–662.

77. Yang, M.A., Gao, X., Theunert, C., Tong, H., Aximu-Petri, A., Nickel, B., Slatkin, M., Meyer, M., Pääbo, S., Kelso, J., and Fu, Q. (2017). 40,000-Year-Old Individual from Asia Provides Insight into Early Population Structure in Eurasia. Curr. Biol. *27*, 3202–3208.e9.

78. Raghavan, M., Skoglund, P., Graf, K.E., Metspalu, M., Albrechtsen, A., Moltke, I., Rasmussen, S., Stafford, T.W., Jr., Orlando, L., Metspalu, E., et al. (2014). Upper Palaeolithic Siberian genome reveals dual ancestry of Native Americans. Nature *505*, 87–91.

79. van de Loosdrecht, M., Bouzouggar, A., Humphrey, L., Posth, C., Barton, N., Aximu-Petri, A., Nickel, B., Nagel, S., Talbi, E.H., El Hajraoui, M.A., et al. (2018). Pleistocene North African genomes link Near Eastern and sub-Saharan African human populations. Science *360*, 548–552.

80. Narasimhan, V.M., Patterson, N., Moorjani, P., Rohland, N., Bernardos, R., Mallick, S., Lazaridis, I., Nakatsuka, N., Olalde, I., Lipson, M., et al. (2019). The formation of human populations in South and Central Asia. Science *365*, eaat7487.

81. Olalde, I., Allentoft, M.E., Sánchez-Quinto, F., Santpere, G., Chiang, C.W.K., DeGiorgio, M., Prado-Martinez, J., Rodríguez, J.A., Rasmussen, S., Quilez, J., et al. (2014). Derived immune and ancestral pigmentation alleles in a 7,000-year-old Mesolithic European. Nature *507*, 225–228.

82. Yu, H., Spyrou, M.A., Karapetian, M., Shnaider, S., Radzevičiūtė, R., Nägele, K., Neumann, G.U., Penske, S., Zech, J., Lucas, M., et al. (2020). Paleolithic to Bronze Age Siberians Reveal Connections with First Americans and across Eurasia. Cell *181*, 1232–1245.e20.

83. Mathieson, I., Lazaridis, I., Rohland, N., Mallick, S., Patterson, N., Roodenberg, S.A., Harney, E., Stewardson, K., Fernandes, D., Novak, M., et al. (2015). Genome-wide patterns of selection in 230 ancient Eurasians. Nature *528*, 499–503.

84. Fu, Q., Li, H., Moorjani, P., Jay, F., Slepchenko, S.M., Bondarev, A.A., Johnson, P.L.F., Aximu-Petri, A., Prüfer, K., de Filippo, C., et al. (2014). Genome sequence of a 45,000-year-old modern human from western Siberia. Nature *514*, 445–449.

85. Fu, Q., Hajdinjak, M., Moldovan, O.T., Constantin, S., Mallick, S., Skoglund, P., Patterson, N., Rohland, N., Lazaridis, I., Nickel, B., et al. (2015). An early modern human from Romania with a recent Neanderthal ancestor. Nature *524*, 216–219.

86. Hajdinjak, M., Mafessoni, F., Skov, L., Vernot, B., Hübner, A., Fu, Q., Essel, E., Nagel, S., Nickel, B., Richter, J., et al. (2021). Initial Upper Palaeolithic humans in Europe had recent Neanderthal ancestry. Nature *592*, 253–257.

87. Prüfer, K., Posth, C., Yu, H., Stoessel, A., Spyrou, M.A., Deviese, T., Mattonai, M., Ribechini, E., Higham, T., Velemínský, P., et al. (2021). A genome sequence from a modern human skull over 45,000 years old from Zlatý kůň in Czechia. Nat. Ecol. Evol. *5*, 820–825.

88. Lazaridis, I., Patterson, N., Mittnik, A., Renaud, G., Mallick, S., Kirsanow, K., Sudmant, P.H., Schraiber, J.G., Castellano, S., Lipson, M., et al. (2014). Ancient human genomes suggest three ancestral populations for present-day Europeans. Nature *513*, 409–413.

89. Jeong, C., Balanovsky, O., Lukianova, E., Kahbatkyzy, N., Flegontov, P., Zaporozhchenko, V., Immel, A., Wang, C.-C., Ixan, O., Khussainova, E., et al. (2019). The genetic history of admixture across inner Eurasia. Nat. Ecol. Evol. *3*, 966–976.

90. Renaud, G. (2018). libbam (Github).

91. 1000 Genomes Project Consortium, Abecasis, G.R., Auton, A., Brooks, L.D., DePristo, M.A., Durbin, R.M., Handsaker, R.E., Kang, H.M., Marth, G.T., and McVean, G.A. (2012). An integrated map of genetic variation from 1,092 human genomes. Nature *491*, 56–65.

92. Gilbert, M.T.P., Kivisild, T., Grønnow, B., Andersen, P.K., Metspalu, E., Reidla, M., Tamm, E., Axelsson, E., Götherström, A., Campos, P.F., et al. (2008). Paleo-Eskimo mtDNA genome reveals matrilineal discontinuity in Greenland. Science *320*, 1787–1789.

93. Fu, Q., Meyer, M., Gao, X., Stenzel, U., Burbano, H.A., Kelso, J., and Pääbo, S. (2013). DNA analysis of an early modern human from Tianyuan Cave, China. Proc. Natl. Acad. Sci. USA *110*, 2223–2227.

94. Fu, Q., Mittnik, A., Johnson, P.L.F., Bos, K., Lari, M., Bollongino, R., Sun, C., Giemsch, L., Schmitz, R., Burger, J., et al. (2013). A revised timescale for human evolution based on ancient mitochondrial genomes. Curr. Biol. *23*, 553–559.

95. Posth, C., Renaud, G., Mittnik, A., Drucker, D.G., Rougier, H., Cupillard, C., Valentin, F., Thevenet, C., Furtwängler, A., Wißing, C., et al. (2016). Pleistocene Mitochondrial Genomes Suggest a Single Major Dispersal of Non-Africans and a Late Glacial Population Turnover in Europe. Curr. Biol. *26*, 827–833.

96. Bollongino, R., Nehlich, O., Richards, M.P., Orschiedt, J., Thomas, M.G., Sell, C., Fajkosová, Z., Powell, A., and Burger, J. (2013). 2000 years of parallel societies in Stone Age Central Europe. Science *342*, 479–481.

97. Sánchez-Quinto, F., Schroeder, H., Ramirez, O., Avila-Arcos, M.C., Pybus, M., Olalde, I., Velazquez, A.M.V., Marcos, M.E.P., Encinas, J.M.V., Bertranpetit, J., et al. (2012). Genomic affinities of two 7,000-year-old Iberian hunter-gatherers. Curr. Biol. *22*, 1494–1499.

98. Benazzi, S., Slon, V., Talamo, S., Negrino, F., Peresani, M., Bailey, S.E., Sawyer, S., Panetta, D., Vicino, G., Starnini, E., et al. (2015). Archaeology. The makers of the Protoaurignacian and implications for Neandertal extinction. Science *348*, 793–796.

99. Ermini, L., Olivieri, C., Rizzi, E., Corti, G., Bonnal, R., Soares, P., Luciani, S., Marota, I., De Bellis, G., Richards, M.B., and Rollo, F. (2008). Complete mitochondrial genome sequence of the Tyrolean Iceman. Curr. Biol. *18*, 1687–1693.

100. Krause, J., Fu, Q., Good, J.M., Viola, B., Shunkov, M.V., Derevianko, A.P., and Pääbo, S. (2010). The complete mitochondrial DNA genome of an unknown hominin from southern Siberia. Nature *464*, 894–897.

101. Günther, T., Malmström, H., Svensson, E.M., Omrak, A., Sánchez-Quinto, F., Kılınç, G.M., Krzewińska, M., Eriksson, G., Fraser, M., Edlund, H., et al. (2018). Population genomics of Mesolithic Scandinavia: Investigating early postglacial migration routes and high-latitude adaptation. PLoS Biol. *16*, e2003703.

102. Vai, S., Sarno, S., Lari, M., Luiselli, D., Manzi, G., Gallinaro, M., Mataich, S., Hübner, A., Modi, A., Pilli, E., et al. (2019). Ancestral mitochondrial N lineage from the Neolithic 'green' Sahara. Sci. Rep. *9*, 3530.

103. Hublin, J.-J., Sirakov, N., Aldeias, V., Bailey, S., Bard, E., Delvigne, V., Endarova, E., Fagault, Y., Fewlass, H., Hajdinjak, M., et al. (2020). Initial Upper Palaeolithic Homo sapiens from Bacho Kiro Cave, Bulgaria. Nature *581*, 299–302.

104. Edgar, R.C. (2004). MUSCLE: multiple sequence alignment with high accuracy and high throughput. Nucleic Acids Res. *32*, 1792–1797.

105. Katoh, K., and Standley, D.M. (2013). MAFFT multiple sequence alignment software version 7: improvements in performance and usability. Mol. Biol. Evol. *30*, 772–780.

106. van Oven, M., and Kayser, M. (2009). Updated comprehensive phylogenetic tree of global human mitochondrial DNA variation. Hum. Mutat. *30*, E386–E394.

107. Kalyaanamoorthy, S., Minh, B.Q., Wong, T.K.F., von Haeseler, A., and Jermiin, L.S. (2017). ModelFinder: fast model selection for accurate phylogenetic estimates. Nat. Methods *14*, 587–589.

108. Minh, B.Q., Schmidt, H.A., Chernomor, O., Schrempf, D., Woodhams, M.D., von Haeseler, A., and Lanfear, R. (2020). IQ-TREE 2: New Models and Efficient Methods for Phylogenetic Inference in the Genomic Era. Mol. Biol. Evol. *37*, 1530–1534.

109. Bouckaert, R., Vaughan, T.G., Barido-Sottani, J., Duchêne, S., Fourment, M., Gavryushkina, A., Heled, J., Jones, G., Kühnert, D., De Maio, N., et al. (2019). BEAST 2.5: An advanced software platform for Bayesian evolutionary analysis. PLoS Comput. Biol. *15*, e1006650.

110. Rieux, A., Eriksson, A., Li, M., Sobkowiak, B., Weinert, L.A., Warmuth, V., Ruiz-Linares, A., Manica, A., and Balloux, F. (2014). Improved calibration of the human mitochondrial clock using ancient genomes. Mol. Biol. Evol. *31*, 2780–2792.

111. Rambaut, A. (2018). FigTree.

112. Gopalakrishnan, S., Sinding, M.S., Ramos-Madrigal, J., Niemann, J., Samaniego Castruita, J.A., Vieira, F.G., Carøe, C., Montero, M.M., Kuderna, L., Serres, A., et al. (2018). Interspecific Gene Flow Shaped the Evolution of the Genus Canis. Curr. Biol. *28*, 3441–3449.e5.

113. Kardos, M., Åkesson, M., Fountain, T., Flagstad, Ø., Liberg, O., Olason, P., Sand, H., Wabakken, P., Wikenros, C., and Ellegren, H. (2018). Genomic consequences of intensive inbreeding in an isolated wolf population. Nat. Ecol. Evol. *2*, 124–131.

114. Sinding, M.S., Gopalakrishan, S., Vieira, F.G., Samaniego Castruita, J.A., Raundrup, K., Heide Jørgensen, M.P., Meldgaard, M., Petersen, B., Sicheritz-Ponten, T., Mikkelsen, J.B., et al. (2018). Population genomics of grey wolves and wolf-like canids in North America. PLoS Genet. *14*, e1007745.

115. Liu, Y.-H., Wang, L., Xu, T., Guo, X., Li, Y., Yin, T.-T., Yang, H.-C., Hu, Y., Adeola, A.C., Sanke, O.J., et al. (2018). Whole-Genome Sequencing of African Dogs Provides Insights into Adaptations against Tropical Parasites. Mol. Biol. Evol. *35*, 287–298.

116. Li, H. (2013). Aligning sequence reads, clone sequences and assembly contigs with BWA-MEM. arXiv, arXiv:1303.3997.

117. Leppälä, K., Nielsen, S.V., and Mailund, T. (2017). admixturegraph: an R package for admixture graph manipulation and fitting. Bioinformatics *33*, 1738–1740.

118. Skoglund, P., Northoff, B.H., and Shunkov, M.V. (2014). Separating endogenous ancient DNA from modern day contamination in a Siberian Neandertal. Proc. Natl. Acad. Sci. USA *111*, 2229–2234.

119. Chen, S., Zhou, Y., Chen, Y., and Gu, J. (2018). fastp: an ultra-fast all-in-one FASTQ preprocessor. Bioinformatics *34*, i884–i890.

120. Kardos, M., Luikart, G., Bunch, R., Dewey, S., Edwards, W., McWilliam, S., Stephenson, J., Allendorf, F.W., Hogg, J.T., and Kijas, J. (2015). Whole-genome resequencing uncovers molecular signatures of natural and sexual selection in wild bighorn sheep. Mol. Ecol. *24*, 5616–5632.

121. Yang, Y., Wang, Y., Zhao, Y., Zhang, X., Li, R., Chen, L., Zhang, G., Jiang, Y., Qiu, Q., Wang, W., et al. (2017). Draft genome of the Marco Polo Sheep (Ovis ammon polii). Gigascience *6*, 1–7.

122. Upadhyay, M., Hauser, A., Kunz, E., Krebs, S., Blum, H., Dotsev, A., Okhlopkov, I., Bagirov, V., Brem, G., Zinovieva, N., and Medugorac, I. (2020). The First Draft Genome Assembly of Snow Sheep (Ovis nivicola). Genome Biol. Evol. *12*, 1330–1336.

123. Zheng, Z., Wang, X., Li, M., Li, Y., Yang, Z., Wang, X., Pan, X., Gong, M., Zhang, Y., Guo, Y., et al. (2020). The origin of domestication genes in goats. Sci. Adv. *6*, eaaz5216.

124. Grossen, C., Guillaume, F., Keller, L.F., and Croll, D. (2020). Purging of highly deleterious mutations through severe bottlenecks in Alpine ibex. Nat. Commun. *11*, 1001.

125. Alberto, F.J., Boyer, F., Orozco-terWengel, P., Streeter, I., Servin, B., de Villemereuil, P., Benjelloun, B., Librado, P., Biscarini, F., Colli, L., et al. (2018). Convergent genomic signatures of domestication in sheep and goats. Nat. Commun. *9*, 813.

126. Martchenko, D., Chikhi, R., and Shafer, A.B.A. (2020). Genome Assembly and Analysis of the North American Mountain Goat (*Oreamnos americanus*) Reveals Species-Level Responses to Extreme Environments. G3 (Bethesda) *10*, 437–442.

127. The GIMP Development Team (2019). Gimp. https://www.gimp.org/.

CellPress
OPEN ACCESS

STAR★METHODS

KEY RESOURCES TABLE

REAGENT or RESOURCE	SOURCE	IDENTIFIER
**Biological samples**		
Soil from Satsurblia cave	This Study	SAT22
Soil from Satsurblia cave	This Study	SAT23
Soil from Satsurblia cave	This Study	SAT26
Soil from Satsurblia cave	This Study	SAT27
Soil from Satsurblia cave	This Study	SAT28
Soil from Satsurblia cave	This Study	SAT29
Canis lupus bone from Satsurblia cave	This Study	Y7a
Canis lupus bone from Satsurblia cave	This Study	Y7d
Canis lupus bone from Satsurblia cave	This Study	AA8c
Canis lupus bone from Satsurblia cave	This Study	T24c
Canis lupus bone from Satsurblia cave	This Study	T20a
**Critical commercial assays**		
NextSeq 500/550 (75 cycle)	Illumina	TG-160-2005
DNeasy Blood and Tissue kit	QIAGEN	69506
Quick Blunting system	NEB	E1201S
Quick Ligation kit	NEB	M2200S
Expand Long Template PCR System	Roche	11681834001
AccuPrimePfx DNA Polymerase	Invitrogen	12344024
MinElute PCR Purification Kit	QIAGEN	28006
Qubit dsDNA HS Assay Kit	Invitrogen	Q32851
Agilent DNA 1000 Kit	Agilent	5067-1504
**Deposited data**		
Raw analyzed data and filtered genomes	N/A	ENA: PRJEB41420
**Software and algorithms**		
Cutadapt 2.7	[18]	https://cutadapt.readthedocs.io/en/stable/
FASTX-toolkit 0.0.1	[19]	http://hannonlab.cshl.edu/fastx_toolkit/
SGA	[44]	https://bioinformaticshome.com/tools/wga/descriptions/SGA.html
Centrifuge 1.0.3	[17]	https://ccb.jhu.edu/software/centrifuge/
Pavian	[45]	https://ccb.jhu.edu/software/pavian/
BWA 0.7.16	[46]	http://bio-bwa.sourceforge.net/bwa.shtml

(*Continued on next page*)

Continued

REAGENT or RESOURCE	SOURCE	IDENTIFIER
Samtools 1.10	[46]	http://samtools.github.io/bcftools/bcftools.html
Bedtools 2.29.2	[47]	https://bedtools.readthedocs.io/en/latest/
Qualimap 2.2.1	[48]	http://qualimap.conesalab.org/
Mapdamage 2.0.9	[49]	https://ginolhac.github.io/mapDamage/
SequenceTools	[50]	https://github.com/stschiff/sequenceTools
Admixtools 5.1	[31]	https://github.com/DReichLab/AdmixTools
Eigensoft 7.2.1	[51]	https://github.com/DReichLab/EIG
ADMIXTURE 1.3.0	[52]	https://bioinformaticshome.com/tools/descriptions/ADMIXTURE.html
PLINK 1.9	[53]	https://zzz.bwh.harvard.edu/plink/
PONG 1.4.9	[54]	https://github.com/ramachandran-lab/pong
ry_compute	[55]	https://github.com/pontussk/ry_compute/blob/master/ry_compute.py
MEGAN 6.19.9	[56]	https://software-ab.informatik.uni-tuebingen.de/download/megan6/welcome.html
BLAST+ 2.10	[57]	https://ncbiinsights.ncbi.nlm.nih.gov/2019/12/18/blast-2-10-0/
Schmutzi	[21]	https://github.com/grenaud/schmutzi/blob/master/.gitmodules
Bamutil 1.0.14	[58]	https://genome.sph.umich.edu/wiki/BamUtil
ContamMix v1.0.10	[32]	N/A
Calico 0.2	[23]	https://github.com/pontussk/calico
Geneious 8.1		http://assets.geneious.com/
Haplogrep 2.0	[59]	https://haplogrep.i-med.ac.at/
BEAST 1.8.4	[60]	https://beast.community/2016-06-17_BEAST_v1.8.4_released.html
Figtree v1.4.4	N/A	http://tree.bio.ed.ac.uk/software/figtree/
Picard Tools 2.21.4	N/A	https://broadinstitute.github.io/picard/
GATK 4.1.4.0	[61]	https://gatk.broadinstitute.org/hc/en-us
samtools 1.9	[46]	http://samtools.github.io/bcftools/bcftools.html

(*Continued on next page*)

CellPress
OPEN ACCESS

Continued

REAGENT or RESOURCE	SOURCE	IDENTIFIER
htsbox pileup r345	N/A	https://github.com/lh3/htsbox
admixturegraph R package	N/A	https://cran.r-project.org/web/packages/admixturegraph/index.html
PMDtools	N/A	https://github.com/pontussk/PMDtools
fastp	[62]	https://github.com/OpenGene/fastp

RESOURCE AVAILABILITY

Lead contact

Further information on materials, datasets, and protocols should be directed to and will be fulfilled by the Lead Contact, Pere Gelabert (pere.gelabert@univie.ac.at).

Materials availability

The raw genomic data used in all the analyses can be accessed at the European Nucleotide Archive (ENA) under ENA: PRJEB41420.

Data and code availability

Sequencing data and the filtered sequences are available at the European Nucleotide Archive (ENA) under ENA: PRJEB41420. All code used in this study and other previously published genomic data is available at the sources referenced in the key resource table.

EXPERIMENTAL MODEL AND SUBJECT DETAILS

We have studied several genomic sequences from soil samples and *C. lupus* bone samples from Satsurblia cave in Georgia.

Archeological context

Satsurblia Cave is located in Georgia in the Southern Caucasus. The cave was discovered in 1975 by N. Kalandadze[63] and was excavated in: 1976, 1985–1988, 2008–2010, 2012–2013, and 2016–2017. Here we present results from those excavated during the 2016 campaign.

Fieldwork in Satsurblia cave focused on excavations in two areas: Area A in the north-western part of the cave, near the entrance, and Area B in the south-west, at the rear part of the cave. The stratigraphic sequence in Area A yielded a wealth of cultural and environmental remains from three main layers: A/I (Eneolithic, 5th millennium BCE), A/II (17.5-16.5 ka cal. BP), and A/III (25- 24 ka Cal. BP). Both areas yielded a series of *in situ* well preserved Upper Palaeolithic occupational layers with f *in situ* living surfaces (“floors”), some of them with preserved fireplaces and rich material culture assemblages. The sequence of Area B is divided into five main archaeological layers and encompasses deposits dated to several UP phases: 13 ka Cal. BP (upper part of Layer B/II); 19 ka Cal. BP (lower part of Layer B/II), 25-24 ka Cal. BP (lower B/II, B/III, B/IV); and 32-31 ka Cal. BP (Layer B/V).[16] A previous genome was published from remains located in square Y5, area A. The radiocarbon dating of this bone is 11,415 ± 50 uncal. bp (OxA-34632).[28] In addition to this bone, fieldwork only recovered one tooth and a fragmented ulna from layers dated between 13-15 ka Cal. BP. H findings were isolated finds and are not part of any burial contexts s. No human remains were found so far from any of the LGM and pre-LGM layers.

Sediment samples were removed with a knife from the exposed profile sections in association with micromorphological block samples and were then stored in zip-up bags and protected from light. Six samples from different layers were sequenced and examined (Data S1A). Here we report genomic data retrieved from sediment sample SAT29, which was taken from Area B, Layer BIII, square X4 (Figure 1B). Micromorphological analyses were performed on block sample SAT 15 14, taken next to SAT29, to investigate the formation processes and post-depositional alterations of layer B/III here, and to assess the integrity of the recovered aDNA and their potential source(s). The following presents preliminary results of these analyses (see Figure 1C). Natural processes include the weathering of the limestone bedrock with the deposition of limestone clasts and calcareous clay and silt; the deposition of rounded, cross-striated soil aggregates that originate from outside the cave; and the redistribution of clay and their deposition as coatings in large voids by percolating water. Both soil aggregates and clay coatings are connected to repeated water activity leaving the associated clay as an unlikely candidate for lasting DNA absorption in this context. Additionally, soil aggregates show variable color expressions, which result from exposure to heat at different temperatures, again making these aggregates an unlikely source for the recovered aDNA. Heating of soil aggregates and other sediment components results from UP people building combustion features at the site and distributing the combusting residues by trampling and dumping behaviors. Fire use and residues resulting from this

behavior - charcoal, ash, burnt sediments and bones - present the dominant anthropogenic component. However, it needs to be noted here that not all bones show heating traces and the heating traces are often limited to charring and low temperature heating. Microscopic bones are sand to gravel size and the most common anthropogenic component in this layer and present a potential source for the recovered aDNA. However, further research into the adsorption and preservation of DNA in archaeological sediments is needed.

METHOD DETAILS

DNA extraction

DNA extraction, library preparation, and indexing steps were undertaken in a dedicated aDNA facility within University College Dublin (UCD). All steps were undertaken within a grade B (EU) clean room under grade A unilateral air-flow hoods. Tyvek suits, hair nets, face masks, and nitrile gloves were used to limit contamination. Extraction of soil DNA was performed using 50 mg of soil in an extraction buffer[64] (to final concentration of 0.45M EDTA, 0.02M Tris-HCl (pH 8.0), 0.025% SDS, 0.5mg/mL Proteinase K and dH2O up to final volume). Samples were incubated at 37°C overnight within Matrix E lysing tubes (MPBIO116914) and using an Eppendorf Thermomixer® C with a rotational speed of 1600rpm. Samples were then cleaned according to the method outlined by Dabney et al.[65] and eluted using TET buffer. DNA libraries of the entire extract were prepared using the method outlined by Meyer and Kircher et al.[66] to produce 25uL of the library. Extraction and library negative controls were utilized using 50μl of deionised water.

PCR, quality control and next generation sequencing

Polymerase Chain Reaction (PCR) amplification and all subsequent steps were undertaken in a grade C laboratory due to increased sample stability. Amplification of 5uL of each library was performed using at a rate of 15 cycles and a single index was added onto the P7 end during amplification.[67] The amplified DNA was cleaned using PB and PE buffers (QIAGEN 28006). Concentration and molarity (nmol/L) of the working solution were ascertained through Agilent 2100 bioanalyzer and a Qubit4 for fluorometric quantification following manufacturer guidelines. Sequencing was undertaken at UCD Conway Institute of biomolecular and biomedical Research on an Illumina NextSeq 500/550 using the high output v2 (75 cycle) reagent kit (Illumina TG-160-2005). Further sequencing was performed on NovaSeq platforms.

Mitochondrial capture

Mitochondrial capture of both the human and canid mtDNA sequences were performed using the method outlined in Maricic et al.[20] Briefly, 50uL of modern human or dog blood were used to extract DNA using the QIAGEN Blood and Tissue kit. The modern DNA of each species was used for a long-range PCR (Sigma Aldrich Expand Long Template PCR System). Two primer pairs were used for the human mtDNA amplification[68] and for the bovine mtDNA amplification[69] and three primer pairs for the dog mtDNA amplification.[70] The long mtDNA fragments were sheared using a sonicator for eight 15-minute sessions and DNA was checked on a 2% agarose gel to make sure that the DNA was fragmented to below 1Kb in length. Next, fragmented DNA was blunt ended using NEB Quick Blunting system and the BioT/B adapters were ligated to the blunted fragments using the NEB Quick Ligation system to produce the bait for capture.

The single-indexed amplified SAT29 library was re-amplified using Accuprime pfx polymerase and the IS5/IS6 primer pairs[66] for 20 cycles and the concentration was measured on the Qubit 4.0. Before capture, the blocking oligonucleotides BO4, BO6, BO8 and BO10 were used to block the sequencing primer sites. Subsequently the bait and pool were combined and incubated at 65°C for two nights. The enriched DNA was melted off the baits using a 2% NaOH solution and the purified DNA was measured using qPCR to determine the ideal cycle number for amplification. The amplified capture libraries were measured on the Qubit 4.0 and Agilent 2100 bioanalyzer to determine concentration and subsequently sequenced on the Illumina Novaseq system.

Human DNA screening

We first explored the six libraries for the presence of human DNA. We obtained an average of 14,893,925 reads in each sequencing library. These reads were processed with the methodology described in Collin 2019.[64] This initial screening showed that five samples exhibited only residual presence of human DNA. The sixth sample, SAT29, had 0.03% of the total reads sequenced map to the human genome. Therefore, this sample was selected for further sequencing. Sequencing and classification results of these samples are presented in Data S1A.

Bioinformatic processing of sample SAT29

After sequencing the SAT29 library to saturation and merging the sequenced reads with the ones from the screening phase, we obtained a total of 561,263,536 reads. These were clipped using Cutadapt 2.7,[44] removing the sequencing adapters and the reads with poly-A tails (reads with more than four As).[44] We also removed reads with qualities below 30 of bases in at least 75% of the read bases with the FASTX-toolkit 0.0.1.[45] Clipped reads were processed with SGA[46] and redundant reads were removed disabling the kmer check. Finally, two bases per end were trimmed and reads shorter than 30bp were discarded using the FASTX-toolkit.[45] Once collapsed and filtered, we obtained a total of 226,880,778 reads that were used for further analyses. We used Centrifuge 1.0.3[17] with default parameters to classify the sequenced reads into taxa using the whole non-redundant nucleotide database from NCBI indexed following the Centrifuge manual and plotted using Pavian.[47] The classification showed the presence of four mammalian

taxa with more than 2% of the Eukaryotic classified reads, which we investigated in further analyses: *Ovis aries*, *Bos* taurus, *Homo sapiens and Canis lupus*. To separate the sequencing reads of the four major mammalian taxa we built a multi-fasta reference file with the genomes of: *H. sapiens* (GRCh37 Assembly GCA_000001405.1), O. aries (Oar_v3.1, assembly, GCA_000298735.1), B. taurus (ARS-UCD1.2 assembly, GCA_002263795.2) and C. lupus (CanFam3.1 assembly, GCF_000002285.3) following a similar strategy described in Feuerborn et al.[71] The filtered reads were aligned with bwa aln[48] disabling seeding, and with a gap open penalty of two. Only reads with mapping qualities above 30 were kept using Samtools 1.10.[72] Duplicated sequences were removed with picard 2.21.4. (Picard-tools., 2018) In total 4,956,676 reads were assigned to these species (661,765 *H. sapiens*, 2,378,237 *C. lupus*, 1,811,555 B. taurus and 72,100 *O. aries*) The characteristics and quality of the mapped reads was assessed with qualimap 2.2.1.[50] We determined the length distribution with fastqc[73] and assessed the level of damage with mapdamage 2.0.9.[51] Although two bases per end were clipped the deamination values are notably high: 3′ deamination of 23% in *Bos*, 28% in *Canis*, 20% in *Homo* and 19% in *Ovis* and 5′ deamination of 25% in *Bos*, 30% in *Canis*, 21% in *Homo* and 20% in Ovis. The distribution of these values along the sequence is presented in Figure 2B.

Damage pattern analysis

Reads mapping to all four species have fragment lengths and deamination patterns typical of ancient DNA (Figures 2B and 2C), but show some slight variability between species (Figure 2C). We examined the relationship between the read length and the deamination values. For doing that we have separated the reads by read length (from 30 to 70 bp) and from (70 to 100 bp) and we examined the deamination in these groups. We have seen that: Human short reads show a deamination of 21% and 19% in the short and long reads 3′ end respectively, that Canis reads show a deamination of 28% and 26% in the short and long reads 3′ end respectively and Bos shows a deamination of 23% and 20% in the short and long reads 3′ end respectively.

BLASTN analysis

In order to check the accuracy of the mapping results we used BLASTN+ from ncbiblastplus 2.11.0,[74] using the whole nt database from NCBI. We excluded the taxa from *Canis, Homo, Ovis and Bos* genera using the flag -negative_seqidlist and setting a minimum expected value (-evalue) of 1e-6 to check the accuracy of our assignment. The results were imported into MEGAN 6.21.1,[57] taxa identification was performed using a lowest common ancestor (LCA) value of 5% of assigned reads.

The 4,956,676 aligned reads were examined with BLAST+ and MEGAN. To prove the accuracy of the mapping, we excluded the four previous cited genera in the analysis. Out of the 4,956,676 aligned reads, 453,049 were assigned by MEGAN using a lowest common ancestor (LCA) score of 1 and a minimum support percent of 5%. 42,958 reads have been assigned to *Pan troglodytes,* representing the 9,5% of the assigned reads. The summed reads assigned to the *Simian* infraorder is 80,988 (18%) of the assigned reads. 79,470 reads (17,5%) have been assigned to the infraorder *Pecora,* which includes *Bison, Bos, Ovis and Capra genera.* 49,426 (11%) reads have been assigned to the suborder *Caniformia*. Other taxa, except these, are *Sus scrofa* with 6,745 reads and *Felis catus* with 10,931 reads. The other identified taxa are represented with less than 1,000 reads. These results show that the mapping process has been selective and the reads have been closely assigned as no species have been identified that could be important sources of the diversity.

Human population genetics

The final 661,765 filtered human reads were used for the following downstream analyses. We used sequenceTools[52] to call pseudo-haplotype genotypes of the 1240K dataset.[75] A total of 11,116 pseudo-haploid positions were recovered. These genotypes were combined with data from 82 ancient genomes[28,29,32,67,75–87] (Table S2) and 2,335 present-day individuals from 149 different populations[88,89] (Table S2) that were projected on a PCA using eigensoft 7.2.1,[53] using the 597,573 SNPs of the Human Origins (HO) dataset.[88] We used the option lsqproject in order to minimize the effect of the missing data on the distortion in the PCA location

Admixture analysis was run using ADMIXTURE 1.3.0[54] with all individuals from the Human Origins (HO) array and all the available sequences from the David Reich lab database (https://reich.hms.harvard.edu/). The HO dataset SNPs were pruned with option–indep-pairwise of PLINK 1.9[55] with parameters 250 50 0.4. The total number of remaining SNPs was 436,097. Figure 2B shows the 82 ancient individuals and SAT29 samples with PONG 1.4.9.[56]

To explore the genetic affinities and the amount of shared derived SNPs we used f_3-outgroup statistics using admixtools 5.1[31] in the form f_3(SAT29,X;Mbuti). X represents both the 82 ancient genomes (Table S2) and the 149 modern populations (Table S2). For the ancient individual comparisons we restricted the analysis to 2,000 shared SNPs and reduced the modern comparisons to 4,000 shared SNPs. We further explored the possible clusterization of SAT29 and Dzuzuana2 individuals with f_4 statistics in the form f_4(Dzuzuana2,X;SAT29,Mbuti), with X representing the ancient tested populations (Table S3). All these comparisons yielded no concluding results due to the lacking statistical significance due to the low coverage.

In addition, we used qpWave from admixtools 5.1 to test the possible single genetic pool for SAT29 and Dzuduana2. We assigned these two populations as left populations and used Chimp, Altai Neanderthal, Ju_hoan_North, Khomani_San and Vindija as the right populations, from the HO dataset. This yielded to non-significant results (tail probability of. of 0.38).

Sex determination of SAT29 human reads

For sex determination we used ry_compute.[19] The results show that the SAT29 soil sample is compatible with a female: R_y value of 0.0089 and a CI of: 0.0078-0.0099. To validate such estimation we have calculated the coverage of the in the mappable region of the

Y chromosome, extracted from 1000 genomes database (http://ftp.1000genomes.ebi.ac.uk/vol1/ftp/release/20130502/supporting/chrY/chrY_callable_regions.20130802.bed) using bedtools 2.29.2 and samtools 1.10. We identified 12,932 filtered reads aligning the mappable region of the human Y chromosome, these reads represent the 0.002% of the chromosome, while the mean value for the rest of chromosomes is 1%, with an SD of 2%.

Neanderthal ancestry in SAT29

We used an F4-ratio[31] to explore the Neanderthal ancestry of the SAT29 sample. We used the genotype data from the 1240k dataset available in https://reichdata.hms.harvard.edu/pub/datasets/amh_repo/curated_releases/V44/V44.3/SHARE/public.dir/v44.3_1240K_public.tar with the combination: (Chimp AltaiNeanderthal: X Mbuti:: Chimp Altai_Neanderthal: VindijaNeanderthal Mbuti). We estimate 1% Neanderthal ancestry in the SAT29 sample, although with large uncertainty due to the low amount of data (95% confidence intervals: 0%–6.6%). This point estimate is similar to that of Dzuzuana2 and likely lower than that of Palaeolithic Europeans due to dilution from Basal Eurasian ancestry.[29] The resolution of the data does not allow a high-precision estimate and this should thus be viewed more as a methodological question exploring the possibilities and limits of sediment DNA, rather than as providing novel and specific insights into the Neanderthal ancestry proportion of the SAT29 environmental genome.

Human mitochondrial analysis

Following the human mtDNA target enrichment step we sequenced 25,483,930 captured reads. After clipping and discarding reads with a base quality score below 30, we had a total of 24,448,710 reads. 2,183,282 reads mapped to the mtDNA human genome. To assure no non-human reads were left after mapping, we used MEGAN 6.19.9[57] and BLAST+ n 2.10[74] to remove non-human reads aligning all the reads against the whole nt database and selecting only the reads that MEGAN locates in the genus *Homo*. After removing duplicates, our final dataset contained 3,477 reads unique to *H. sapiens*, which represents 10.08-fold mtDNA genome coverage. The deamination rate of the mtDNA was 0.48 G > A at the 3′ end and 0.47 C > T at the 5′ end

mtDNA mixture estimate

We run contamMix (0.0.1),[32] Schmutzi,[21] and Calico 0.2[23] for the SAT29 human reads, while for the *Canis* sequences only two methods were used: ContamMix and Calico. The contamination levels in the *Bison* reads were based on ContamMix estimates alone. We also used the deamination values to check the presence of modern contamination by estimating these values in reads longer and shorter than 70 bp observing no difference in the obtained values between both ins (0.47 and 0.49).

Presence of multiple individuals identification

We used a similar strategy to the one described in Slon et al.[13] to explore the diversity in the SAT29 mitochondrial human reads. We filtered the reads that showed the presence of deaminated bases in the last ten positions on both ends with libbam,[90] and then we explored the allele distribution in a set of diagnostic and segregating positions through the base calling using samtools mpileup and the visual observation of reads through IGV. We also estimated the amount of deamination in the last 10 bases of the examined reads (Table S1). This data is also displayed graphically in Figure S2B.

We explored the allele frequency of the human environmental genome at all the 1000 genomes[91] variant positions of the mitochondrial sequence. First, we used GATK 3.7[62] Unified Genotyper to call all the mitochondrial variable positions from 1000 genomes on the filtered reads, a total of 3892 variants, using -L and with the out_mode EMIT_ALL_SITES, using the metagenomic filtered file as input. This has resulted in the identification of 130 sites with diverse alleles, 122 out of its being segregating. To estimate the effect of ancient damage in these sites we have plotted differentially the polymorphic and non polymorphic sites. The distribution of the allele frequencies of these sites is displayed in Figure S1C.

mtDNA consensus calling

To minimize the effect of low coverage, diversity and damage, we have used Geneouis to call the mtDNA genotypes based on the majority allele (calling the base supported by > 50% of the reads) for positions covered by at least five reads.

Human mitochondrial tip dating

The full mitochondrial dataset[28,84,88,92–103] (Table S2) was aligned using *MAFFT v7.309*,[104,105] Poly-C regions and mutation hotspots in positions 303-315, 515-522 and 16519 were masked in the consensus fasta.[106] The resulting alignment was used for assessing the nucleotide substitution model using IQ-tree.[107,108] The model TN+F+I+G4 had the lowest Bayesian information criterion. We used the alignment as input for BEAUti version 2.6.3, setting as priors the radiocarbon dates shown in Table S2. Tip dates were set to the years before the present. BEAST 2.6.3[109] was run with a 50,000,000 MCMC chain length with a strict clock model, Bayesian skyline tree prior and a mean clock rate of 2.2×10^{-8}.[110] The resulting log files were viewed with Tracer v1.7.1 and were checked for ESS above 200. The tree files were annotated with TreeAnnotator v2.6.3 and the resulting annotated trees were viewed with Figtree v1.4.4.[111]

To complement the Bayesian tree we inferred one Maximum Likelihood tree using the same alignment previously used for the Bayesian analyses. The tree was inferred with IQ-tree[108] with 100 bootstrap repetitions. Resulting annotated trees were viewed with Figtree v1.4.4.

Wolf genome: comparative dataset

To construct a dataset for ancestry analyses of the SAT29 reads of canid origin, we started from a previously compiled variant call set encompassing 722 dogs, wolves and other canid species.[33] We also incorporated a number of additional wolf and other canid genomes from other publications.[112–115] These additional genomes were mapped to the dog reference genome using bwa mem version 0.7.15,[116] marked for duplicates using Picard Tools version 2.21.4 (Picard-tools., 2018), genotyped at the sites present in the 722 canid variant call set using GATK HaplotypeCaller v3.6[62] with the "-gt_mode GENOTYPE_GIVEN_ALLELES" argument, and then merged into the variant call set using bcftools merge (http://www.htslib.org/).

The variants and genotypes were then filtered by excluding sites displaying excess heterozygosity ("ExcHet" annotation p value < 1x10-6, as computed using the bcftools fill-tags plugin), setting to missing any genotypes that included an indel allele or any allele with a frequency lower than the two most common alleles at the site and thereby removing such alleles (thus retaining only two SNP alleles overlapping any given position), setting genotypes to missing if the depth at the site (computed as the sum of the "AF" fields) was lower than one third of the genome-wide average coverage of the same, or lower than 5, or higher than twice the average, normalizing allele representation using bcftools norm, and finally excluding sites with missing genotypes for 130 or more individuals. This resulted in 65.5 million SNPs, of which 19.2 million are transversions with a minor allele count in the dataset of at least two.

We assigned genotypes to the SAT29 reads that mapped to the dog genome by sampling one random allele at each of these variants using htsbox pileup r345 (https://github.com/lh3/htsbox) and requiring a minimum read length of 35 ("-l 35"), mapping quality of 30 ("-q 30") and base quality of 30 ("-Q 30"). We also included data from two Pleistocene Siberian wolves, the 35,000 year old Taimyr-1 from the Taimyr peninsula[34] and the 33,000 year old CGG23 from the Yana RHS site in eastern Siberia,[35] and genotyped these in the same way as the SAT29 data. The SAT29 sample obtained genotype calls at 1,532,986 of the total set of SNPs (2.34%), and 439,426 of the transversions (2.28%).

To complement the bayesian tree we performed one Maximum Likelihood tree using the same alignment previously used for the bayesian analyses. The tree was performed with IQ-tree[108] with 100 bootstrap repetitions. Resulting annotated trees were viewed with Figtree v1.4.4.

Wolf genome: ancestry analyses

We calculated all possible f_4-statistics involving SAT29 and the publicly available canid genomes using AdmixTools v5.0,[31] using the qpDstat command with the "$f_4$mode: YES" and "numchrom: 38" arguments.

Using f_4-statistics of the form f_4(AndeanFox,SAT29;X,Wolf35Xinjiang) we find that the SAT29 canid data is closer to a member of *Canis lupus*, Wolf35 from Xinjiang, China, than to representatives of Coyote (Z = 31.37), Golden Jackal (Z = 37.58), African Golden Wolf (Z = 31.01) and Dhole (Z = 90.20). The strongly positive values in all tests shows that the data is clearly from a member of the wolf/dog species as opposed to any of these other canid species.

We used the admixturegraph R package[117] to systematically test admixture graphs by fitting them to the *f4*-statistics. We enumerated all possible graphs involving a coyote (Coyote01, California), a modern Eurasian wolf (Wolf35, Xinjiang), a modern dog (New Guinea singing Dog, pooling individuals NewGuineaSingingDog01, NGSD1, NGSD2 and NGSD3), the two Pleistocene Siberian wolves and the SAT29 sample without admixture events. To each of these admixture graphs, we then grafted on the dog reference genome as a clade with the New Guinea singing dog, and then a boxer individual (Boxer01) as a clade with the reference genome. Because the dog reference genome also derives from a boxer, but a different individual, the contrast between these two can serve to quantify reference bias in other genomes. We therefore introduced, in each graph, an admixture event from the reference genome into each of the ancient genomes – this "reference admixture" can then correct for any systematic shifts in allele frequency caused by reference bias in these genomes. Each graph was then fit five times, retaining the fit that achieved the lowest "best_error" score. Out of the 100 possible graphs, three provided good fits to the data, with the difference between them being only the placement of the SAT29 branch as being on, downstream of, or upstream of the Siberian Pleistocene wolf branch. They correctly predict 209 out of the 210 possible f_4-statistics ($|Z| < 3$) with the minor outlier statistic: f_4(CoyoteCalifornia,NewGuineaSingingDog ; Wolf35Xinjiang,CGG23). After these three, the next best graph has 26 outlier statistics.

Canid bone testing

We screened five samples from different layers and areas of Satsurblia cave (Data S1D) for two main reasons to A) compare the bone DNA and the canid DNA from soil, and B) determine the differential capacity to retrieve DNA from different sources of the same geological layer. In the last excavations no other human bones from the Satsurblia cave have been recovered, however several Canis bones have been identified. We extracted DNA and prepared libraries following the same procedure described for soil. We also captured the dog mtDNA with the same strategy previously described. Two libraries have shown enough DNA to be analyzed: T20a from layer IIa and sample Y7d from layer BIVa. We have used these samples in the *Canis* mt phylogenies canis molecular datings.

Canid mitochondrial tip dating

We generated a consensus sequence for the SAT29 canid mitochondrial capture data in the same way as we did for the human reads: using Geneious with the major (> 50%) allele for basecalling and mtDNA positions covered by at least five reads.

A full set of samples (107 as shown in Table S4) was aligned using MAFFT v7.309[105] and used for assessing the nucleotide substitution model using IQ-tree.[107,108] The model HKY+F+I+G4 had the lowest Bayesian information criterion. The resulting fasta

alignment was used as an input file for BEAUti version 2.6.3, setting as priors the radiocarbon dates shown in Table S5. Tip dates were set to the years before the present. A strict clock model and Bayesian skyline tree prior were used. The tree height prior was based on a normal distribution with a mean value of 125 kya according to estimates obtained by Loog et al.[37] SAT29 was given a prior age of 25,000 years BP based on a broad normal distribution with standard deviation of 15000 years. BEAST was run with a 50,000,000 MCMC chain length. The resulting log files were viewed with Tracer v1.7.1 and were checked for ESS above 200. The tree files were annotated with TreeAnnotator v2.6.3 and the resulting annotated trees were viewed with Figtree v1.4.4.

To complement the bayesian tree we performed one Maximum Likelihood tree using the same alignment previously used for the bayesian analyses. The tree was performed with IQ-tree[108] with 100 bootstrap repetitions. Resulting annotated trees were viewed with Figtree v1.4.4.

Presence of multiple individuals

We found evidence for polymorphism in the SAT29 wolf mitochondrial sequences, which could suggest that the retrieved DNA originates from more than one individual. The SAT29 consensus sequence falls on a branch together with two pre-LGM Armenian wolves (TU9 and TU10), but on many sites that define this branch SAT29 also displays observations of the reference allele. We summarized this evidence as follows:

1. We aligned the previously published ancient and modern wolf mitochondrial genomes to the dog mitochondrial reference genome using bwa mem 0.7.17[116] with the "-x intractg" argument, and obtained genotypes for them using htsbox pileup.
2. We merged the SAT29 mitochondrial reads obtained from the targeted capture experiment with those obtained from the shotgun sequencing experiment, to achieve a total coverage of 16.6x (mapping quality $\geq$ 30, base quality $\geq$ 30, read length $\geq$ 35).
3. To reduce the impact of ancient DNA damage and sequencing error on the assessment of polymorphism, we restricted the analysis to a set of polymorphic sites ascertained among the previously published wolf mitochondrial genomes. We first identified sites where the two samples CGG18 (Siberia, 41.7k BP) and TH10 (Alaska, 21k BP) carry the same nucleotide, as a rough approximation of the ancestral sequence of the "major clade" of wolf mitochondria to which these two samples, as well as the majority of ancient and present-day sequences, belong. Any sites containing indels were excluded. We then identified 79 sites on which the Armenian TU10 sample carries a different nucleotide from this major clade. This should constitute a set of variants where SAT29 often should carry the TU10 allele due to its shared phylogenetic history, but might carry the major clade allele if there are other sequences in the sample that carry alleles from that clade.
4. We then counted the number of alleles in the SAT29 sample matching the "Armenian" allele and the "major clade" allele at each of these ascertained sites, using htsbox pileup (-q30 -Q30 -l 35). Both alleles are observed at most sites (57 out of 75). A few sites (11 out of 75) display only the major clade allele, but this likely reflects more recent, private mutations in the history of TU10 after its divergence from the SAT29 sequence.
5. We restricted the SAT29 sample to reads displaying evidence of ancient DNA deamination damage, using PMDtools[118] with the "–threshold 3" argument. While the total read counts are reduced, most sites still display both alleles. This suggests that the additional mitochondrial sequences(s) in the sample are also of ancient origin, rather than representing modern contamination. These results are displayed in Figure S2A.

Bison genome: comparative dataset

To construct a dataset for ancestry analyses of the SAT29 reads of bovid origin, we downloaded raw sequence reads from the European Nucleotide Archive (ENA) from a number of previously sequenced bovid genomes: present-day gaur,[40,41] present-day gayal and banteng,[40] present-day and ancient domestic taurine and zebu domestic cattle,[41] ancient aurochs,[41] American bison,[40] present-day European bison from Poland,[40,42] and the historical (early 20th century) European bison from Poland and the Caucasus.[42]

We preprocessed the reads from all the samples using fastp,[119] filtering through the automatic adaptor detection and trimming that applied by default, as well as the "–low_complexity_filter" and "–length_required 30" arguments. For ancient genomes that had been sequenced paired-end, the "–merge" option was applied and only successfully merged read pairs were retained.

We mapped the filtered reads to the domestic cattle reference genome, using bwa mem v0.7.17[116] in paired-end mode for modern genomes and bwa aln v0.7.17[48] in single-end mode, with permissive parameters ("-l 16500 -n 0.01"), for the ancient genomes. We assigned read groups according to the library and run information specified in the ENA metadata for each of the studies, merged reads for each sample and sorted using samtools,[72] and marked duplicate reads using Picard MarkDuplicates v2.21.4 (Picard-tools., 2018).

To define a set of variants to use for ancestry analyses, we identified heterozygous sites in the genome of a single, high-coverage gaur, sample Ga5.[41] Ascertainment in the gaur outgroup species, which is estimated to have diverged from bison more than half a million years ago,[40] should result in variants that behave in an unbiased fashion in ancestry analyses. We called genotypes in Ga5 using GATK HaplotypeCaller v3.6.[62] We then filtered these genotype calls using bcftools (http://www.htslib.org/) to retain only those variants that were SNPs, were located on the 29 autosomal chromosomes, had a heterozygous genotype, had a genotype quality (GQ field) of > 30, a depth (sum of AD fields) of more than 15.04 and less than 49.63 (corresponding to 0.5 and 1.65 times the average autosomal coverage of 30.08, respectively). This resulted in 4,930,425 SNPs, of which 1,447,767 are transversions.

We assigned pseudo-haploid genotypes for all the bovid genomes, including Ga5 itself and the SAT29 reads that mapped to the cattle genome, by sampling one random allele at each of these Ga5 ascertained SNPs, using htsbox pileup r345 (https://github.com/lh3/htsbox) and requiring a minimum read length of 35 ("-l 35"), mapping quality of 30 ("-q 30") and base quality of 30 ("-Q 30"). The SAT29 sample obtained genotype calls at 94,262 of the total set of SNPs (1.91%), and 27,724 of the transversions (1.91%).

Bison genome: ancestry analyses

We calculated all possible f_4-statistics involving the SAT29 sample and the publicly available bovid genomes using AdmixTools v5.0,[31] using the qpDstat command with the "f_4 mode: YES" and "numchrom: 29" arguments.

Using f_4-statistics of the form f_4(Ga5.Gaurus,SAT29;X,Wisent11) we find that the SAT29 bovid data is closer a bison individual, Wisent11 from Poland, than to representatives of aurochs (Gyu2, Armenia, Z = 20.59), taurine cattle (ScottishHighland, Z = 22.73), Zebu cattle (Tharparkar, Z = 24.75), banteng (ypt2230, Z = 35.96) and gayal (1107, Z = 43.76). The strongly positive values in all tests shows that the data is clearly from a member of the bison species as opposed to any of these other bovid species.

We used the admixturegraph R package[117] to systematically test admixture graphs by fitting them to the f_4-statistics. We enumerated and fit all possible graphs involving a gaur (Ga5), an American bison (mzc), an historical Polish bison (PLANTA), a historical Caucasian bison (Cc1) and the SAT29 sample with up to one admixture event. Each graph was fit five times, retaining the fit that achieved the lowest "best_error" score.

Among the 15 possible topologies that relate these five genomes without any admixture events, the best-fitting graph has the American bison as basal to the European bison and SAT29, and then SAT29 as basal to the historical Polish and Caucasian bison. This graph has just one outlier f_4-statistic ($|Z| < 3$), which fails to account for excess affinity between the American and the Polish bison (f_4(Gaur,-American bison;Polish bison,SAT29), Z = -4.03). After this, the next two best-fitting graphs differ from the best-fitting topology in that the position of SAT29 is swapped with that of the historical Polish wisent or the historical Caucasian wisent, respectively. These graphs both feature the same three outlier f_4-statistics, the first of which is shared by the best-fitting graph above, and the second and third of which fail to account for shared drift between the historical European bison to the exclusion of SAT29 (f_4(Gaur,Causasian bison;Polish bison,SAT29), Z = -4.51, f_4(Gaur,Polish bison;Caucasian bison, SAT29), Z = -3.43.f_4

Following these, all other graphs without admixture events have seven or more outlier f_4-statistics. When allowing for one admixture event, 10 out of the 315 possible graphs fit the data without any outlier statistics. Multiple solutions with quite variable topologies are thus possible, and with the limited data available we do not attempt to discriminate between these.

Bison mitochondria capture and analysis

We have captured a *B. bonasus* environmental mitochondrial genome through *Bos taurus* baits designed with the same methodology previously described. After aligning the reads against the *B. bonasus* mitochondria reference (NC_014044.1), we have filtered the reads with BLASTN and MEGAN as described previously. We have aligned the retrieved filtered reads with other 70 Bovid samples (Table S5) using MAFFT v7.309[105] and used it for assessing the nucleotide substitution model using IQ-tree.[107,108] We generated a bayesian tree and a calibrated tip-dating phylogeny with BEAST 2, setting as priors the radiocarbon dates shown in Figure S6.

Ovis genomic analysis

We explored the possible phylogenetic position of the reads that aligned to the Oar_v3.1 genome within the *Ovis* and *Capra* genus. In order to determine the SNPs to compare to SAT29, we built a dataset with individuals from all the available species of the genus *Ovis* and *Capra: Ovis vignei* (Nextgen project: https://projects.ensembl.org/nextgen/), *Ovis aries* (Nextgen project: https://projects.ensembl.org/nextgen/), *Ovis canadensis*,[120] *Ovis ammon*,[121] *Ovis orientalis* (Nextgen project: https://projects.ensembl.org/nextgen/), *Ovis nivicola*[122] Capra hircus (Nextgen Project: https://projects.ensembl.org/nextgen/), *C. caucasica*,[123] *C. ibex*,[124] *C. aegagrus*[125] and Oreamnos americanus as an outgroup.[126] We downloaded the available VCF files of the following genomes: 75 *Ovis aries* genomes, 4 *Ovis vignei* genomes, 14 *Ovis orientalis* genomes and one Capra hircus. This dataset consists of 48,870,177 SNPs in the autosomal chromosomes of the *Ovis aries* genome. After filtering SNPs for MAF < 0.05 and removing non-SNPs and no-biallelic SNPs and SNPs not located in autosomes, 22,553,044 SNPs were kept.

As the available VCF files does not cover all species we wanted to include, we downloaded the FASTQ files of one Ovis canadensis, one Ovis nivicola, one Ovis ammon, one C caucasica, one Capra ibex, one C sibirica, two C aegagrus and one Oreamnos americanus to produce additional VCF files for downstream analyses. The sequencing reads of the FASTQ files were aligned with BWA,[48] duplicate reads were removed with picard and low quality reads (< 30) were removed with Samtools.[72] These filtered reads were used for variant calling with GATK HaplotypeCaller v3.6 (67) by genotyping the positions of the filtered dataset with the "-gt_mode GENOTYPE_GIVEN_ALLELES" argument. The reads were then merged into the variant call set using bcftools merge (http://www.htslib.org/). Genotypes were filtered with bcftools for: MBQ > 30, depth of coverage below the half of the average coverage and more than double of the average coverage to eliminate possible misalignments in low and high complexity regions. These new VCF files were then merged with the downloaded VCF files. The final dataset was filtered for positions with more than 10% of missing sites and excess of Heterozygosity (pval < 1.10-6).

We called pseudo-haplotype genotypes using the 22 million positions of SAT29 Ovis reads using Sequence Tools[52] and recovered 19,469 SNPs of the SAT29 genome. We used f_4 statistics from admixtools[31] to determine the closest taxa to the SAT29 sample. The analysis did not yield any concluding result as the number of SNPS is likely too low.

All the displayed images in the publication have been edited with GIMP 2.10.24.[127]

Current Biology, Volume *31*

Supplemental Information

Genome-scale sequencing and analysis

# of human, wolf, and bison DNA

from 25,000-year-old sediment

Pere Gelabert, Susanna Sawyer, Anders Bergström, Ashot Margaryan, Thomas C. Collin, Tengiz Meshveliani, Anna Belfer-Cohen, David Lordkipanidze, Nino Jakeli, Zinovi Matskevich, Guy Bar-Oz, Daniel M. Fernandes, Olivia Cheronet, Kadir T. Özdoğan, Victoria Oberreiter, Robin N.M. Feeney, Mareike C. Stahlschmidt, Pontus Skoglund, and Ron Pinhasi

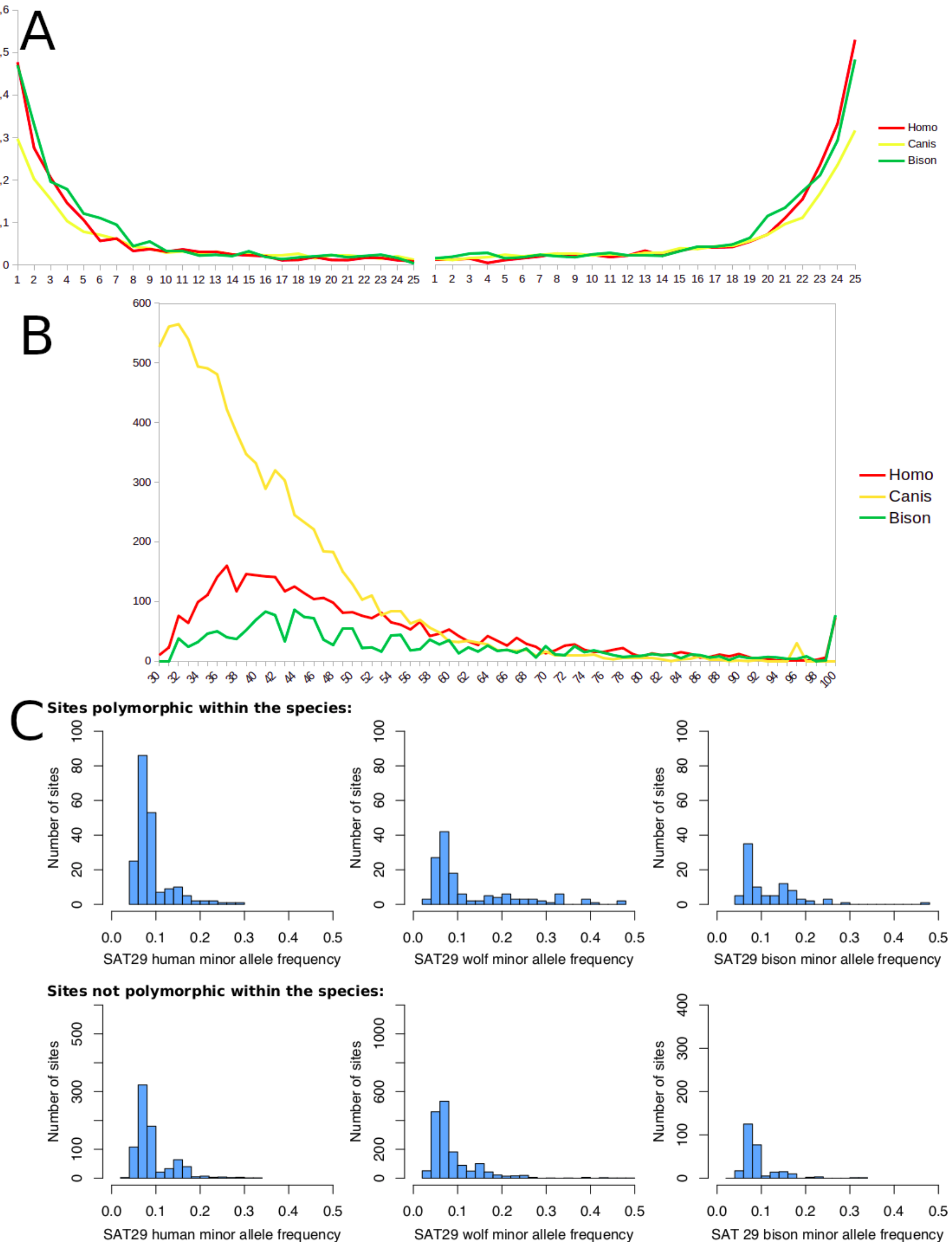
A
Homo
Canis
Bison
B
Homo
Canis
Bison
C
Sites polymorphic within the species:
Number of sites
SAT29 human minor allele frequency
SAT29 wolf minor allele frequency
SAT29 bison minor allele frequency
Sites not polymorphic within the species:
SAT29 human minor allele frequency
SAT29 wolf minor allele frequency
SAT 29 bison minor allele frequency

Figure S1: Mitochondrial captured reads, Related to Figure 2: A) Deamination patterns. B) read length distributions for the captured mitochondrial reads. C) Minor allele frequency distributions for the three captured mitochondrial genomes. Only positions covered by at least 12 reads are included, and counts of sites with a minor allele frequency of 0 are omitted. The top row shows sites that have been ascertained to be polymorphic within the given species, while the bottom row shows the rest of the sites. This is informative about the relative coverage of the two alleles at each position. Most of the values fall between 0 and 0.2, which likely reflect the confounding effects of ancient DNA damage (e.g. 1 or 2 damaged reads out of 10-15 total reads). However, particularly in the wolf data, there are also quite a few reads that show minor allele frequencies larger than 0.2, especially at the sites harbouring known polymorphisms, which likely reflects the presence of more than one haplotype in the sample.

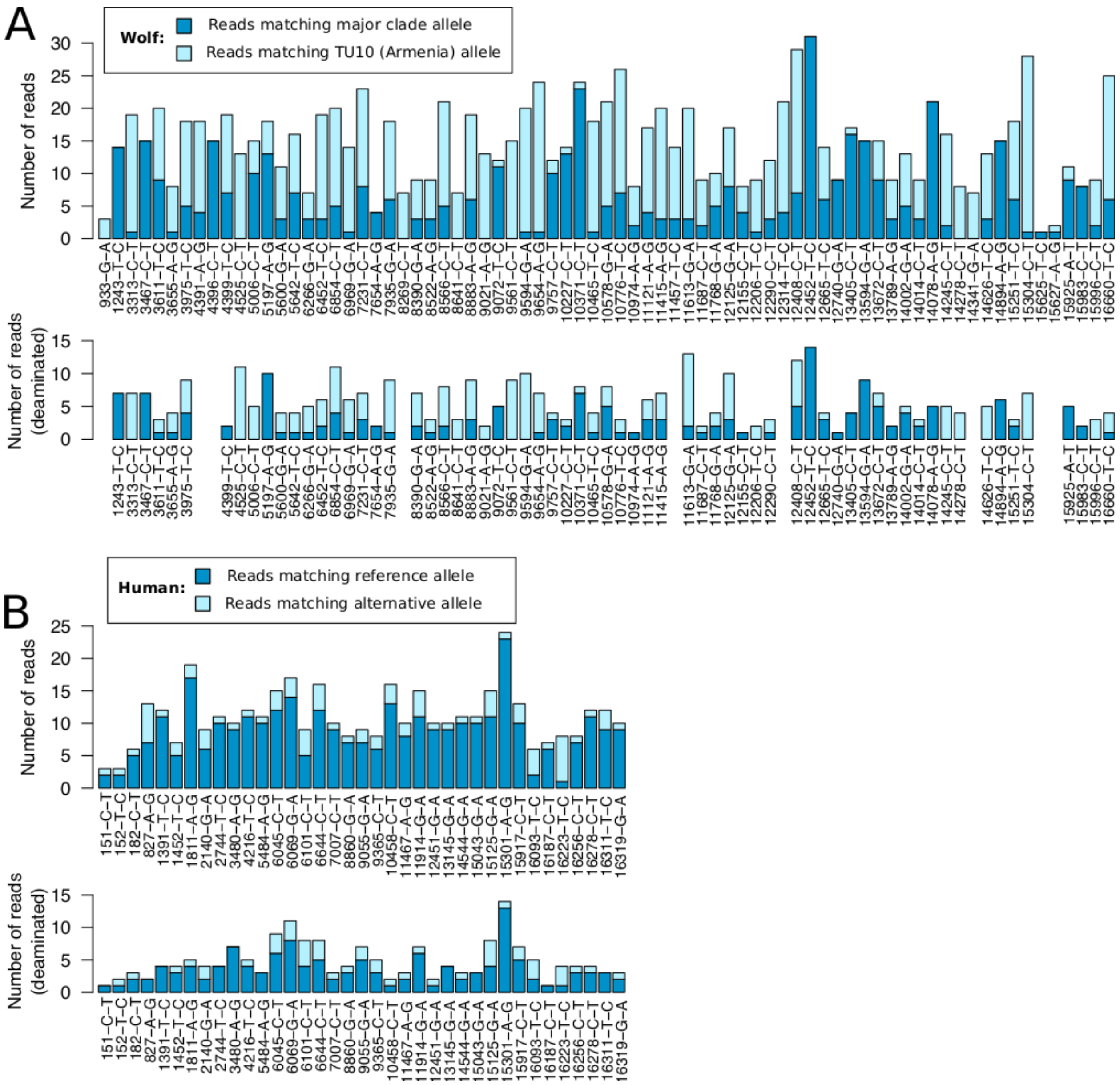

Figure S2: Evidence for polymorphism in the SAT29 wolf and human mitochondrial sequences, Related to Table 1: A) Sites that differ between the pre-LGM Armenian wolf TU10 and the major clade of wolf mitochondria were identified, and the read counts of the SAT29 sample at these sites displayed. The top row shows results using all reads, and the bottom row using only reads that display evidence of deamination. These results suggest that the retrieved DNA derives from more than one

wolf individual. B) Sites that display some evidence of possible polymorphism in the human SAT29 mitochondrial data were identified (Table S2), and the reads counts of the SAT29 sample at these sites displayed The top row shows results using all reads, and the bottom row using only reads that display evidence of deamination. The results suggest that much of the diversity signal in the human data is explained by damage, but some allele observations could also reflect genuine, low-level diversity in the sample.

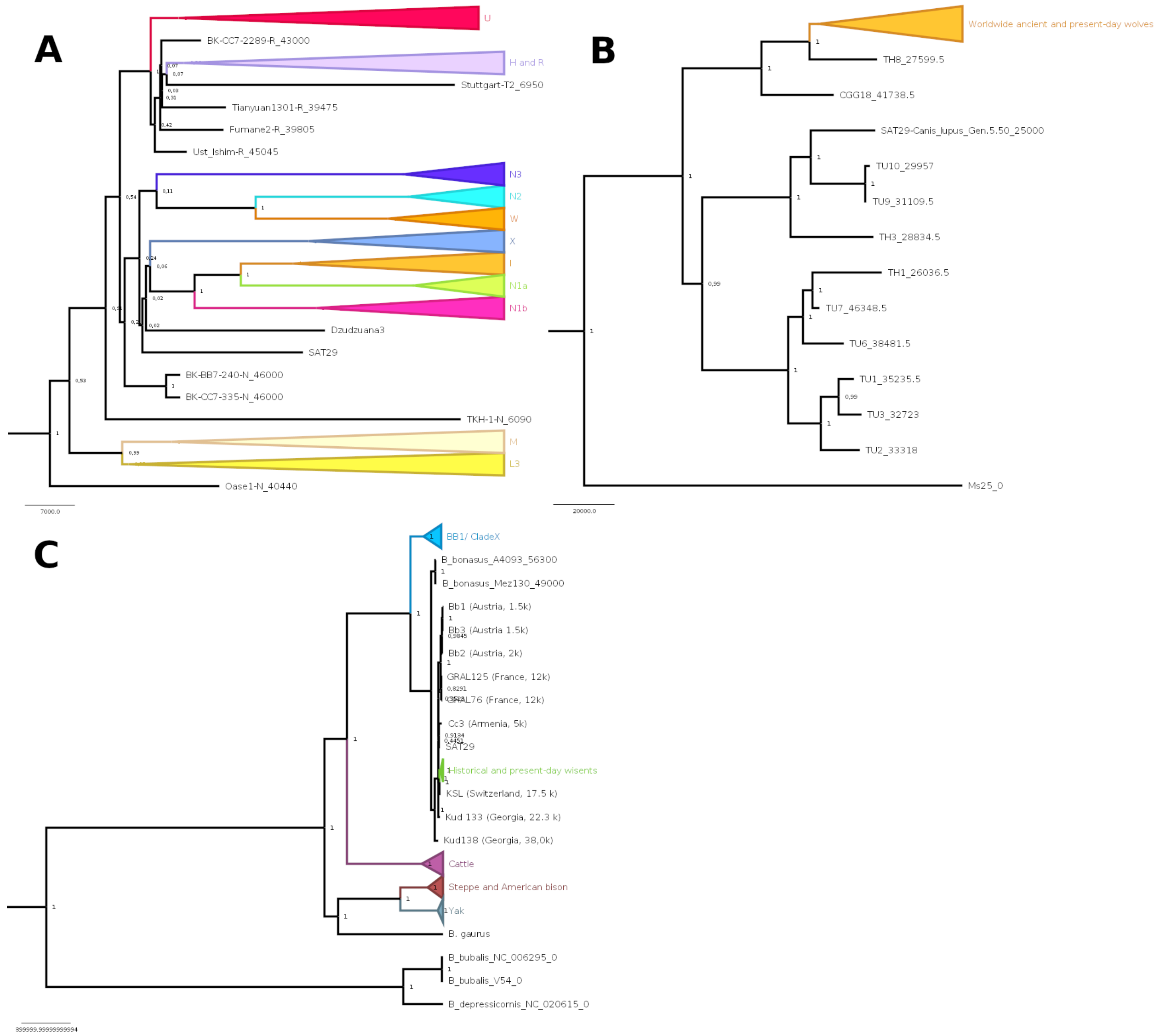

Figure S3, Related to Figures 3, 4 and 5: Maximum likelihood trees performed with the mitochondrial datasets. The node values represent the bootstrapping value. **A)** Homo, **B)** Canis and **C)** Bison.

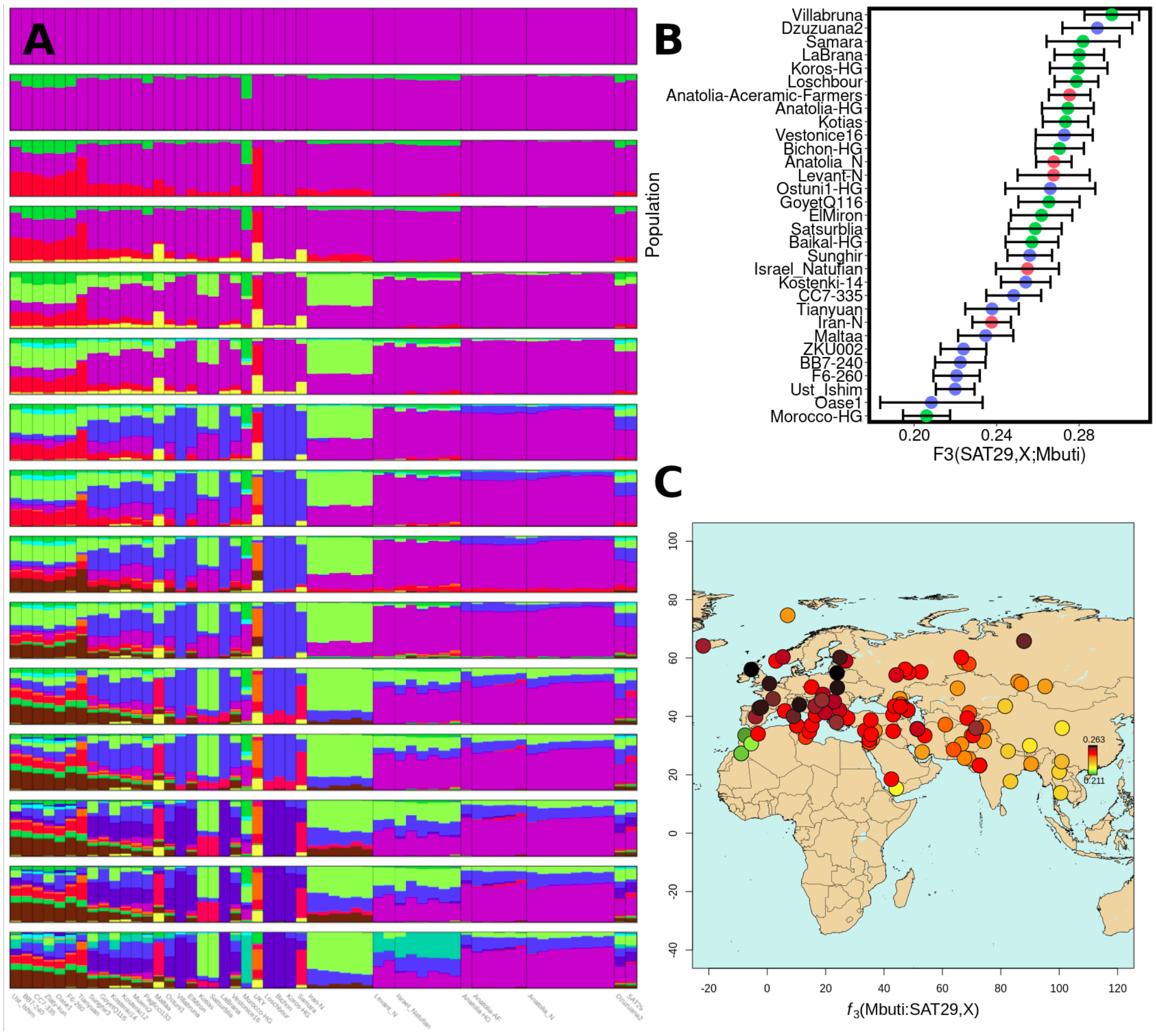

Figure S4: Human Population Genetics, Related to Figure 3: A) ADMIXTURE plots using K from 1 to 15 on the human dataset, **B)** Values of outgroup-f_3 statistics of the form f_3(SAT29,X;Mbuti). Only statistics with more than 2,000 SNPs were included. Green dots represent post-LGM samples, violet dots represent pre-LGM samples and red dots represent farmer samples. Error bars denote one standard error. **C**) Values of the statistic f_3 (SAT29,Modern-population;Mbuti), the intensity of color is proportional to the amount of shared drift.

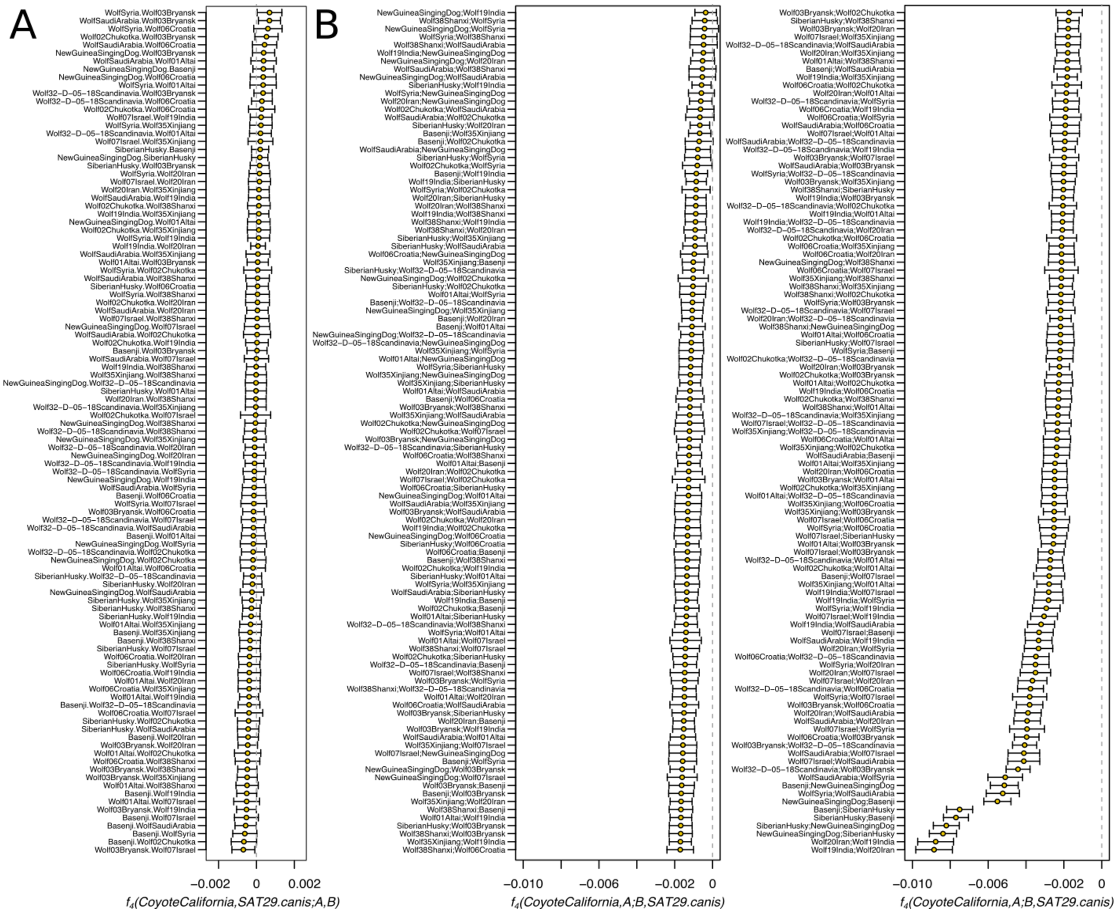

Figure S5: The SAT29 *Canis* genome is symmetrically related to modern wolves and dogs, and the latter share drift to the exclusion of SAT29, Related to Figure 4: f_4-statistics evaluating the basal ancestry status of the SAT29 canid genome in relation to a set of 15 diverse Eurasian present-day wolves and dogs. Error bars denote ±3 standard errors. A) Statistics of the form f_4(CoyoteCalifornia,SAT29.*Canis*;A,B) are consistent with being distributed around 0, indicating that SAT29 is symmetrically related to present-day individuals. A few statistics fall close to or slightly outside $|Z| > 3$ (largest $|Z| = 3.43$), and involve wolves from Southwestern and South Asia (WolfSyria, WolfSaudiArabia, Wolf07Israel, Wolf19India). However, the values of these statistics are in the direction of SAT29 being closer to other wolves than to these wolves, and thus likely reflects some basal ancestry in these present-day wolves, e.g. due to admixture

from non-wolf canids. B) Statistics of the form f_4(CoyoteCalifornia,A;B,SAT29.*Canis*) are all negative, indicating that all of the present-day wolves and dogs share genetic drift that is not shared with the SAT29 sample.

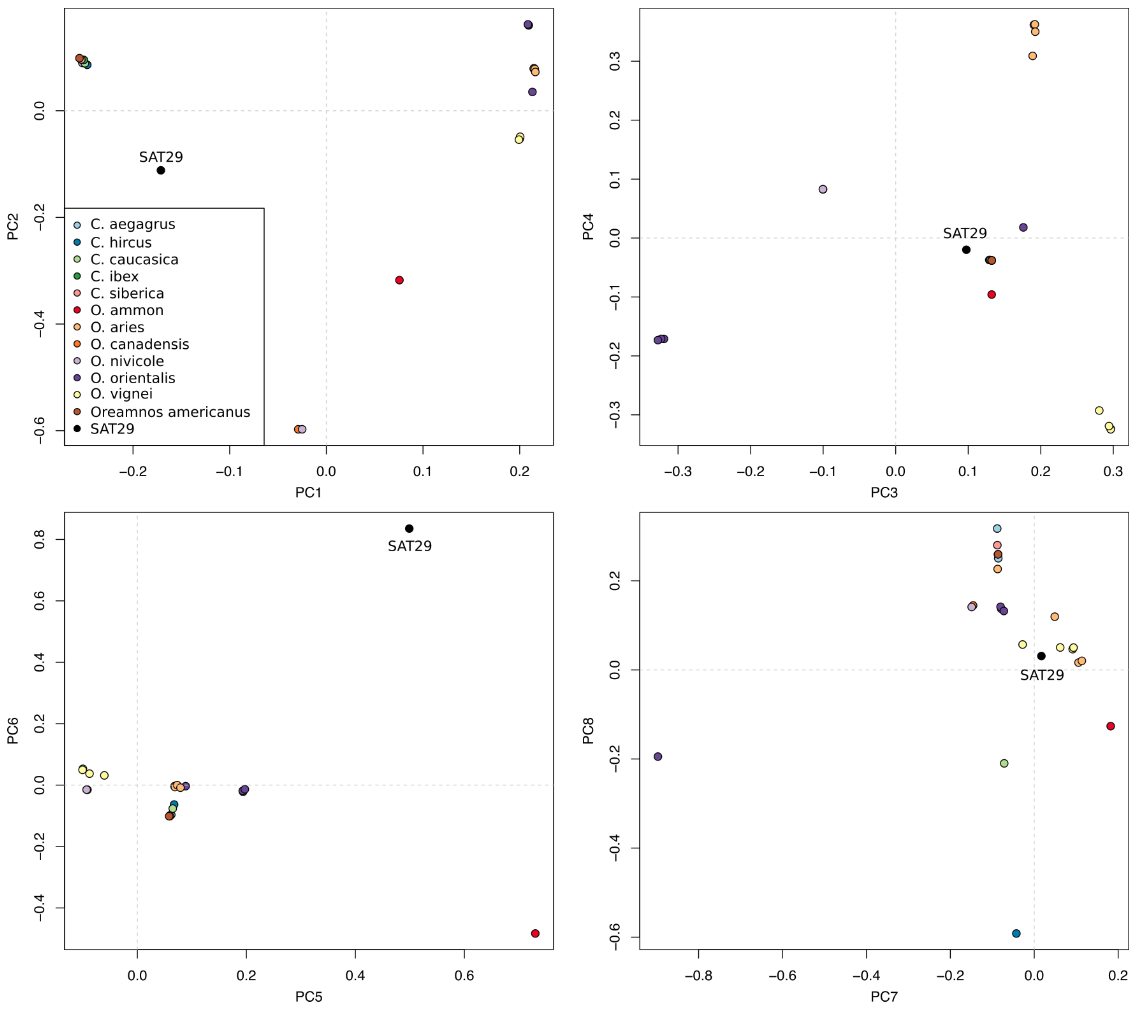

Figure S6: Principal component analysis on the *Ovis* and *Capra* dataset, Related to Figure 2: The analysis was performed on a distance matrix comparing per-sample profiles of all possible f_4-statistics of the form f_4(X,A;B,C) where X is the focal sample.

Supplementary References

S1. Pinhasi, R., Meshveliani, T., Matskevich, Z., Bar-Oz, G., Weissbrod, L., Miller, C.E., Wilkinson, K., Lordkipanidze, D., Jakeli, N., Kvavadze, E., et al. (2014). Satsurblia: new insights of human response and survival across the Last Glacial Maximum in the southern Caucasus. PLoS One *9*, e111271.

S2. Lazaridis, I., Patterson, N., Mittnik, A., Renaud, G., Mallick, S., Kirsanow, K., Sudmant, P.H., Schraiber, J.G., Castellano, S., Lipson, M., et al. (2014). Ancient human genomes suggest three ancestral populations for present-day Europeans. Nature *513*, 409–413.

S3. Mathieson, I., Lazaridis, I., Rohland, N., Mallick, S., Patterson, N., Roodenberg, S.A., Harney, E., Stewardson, K., Fernandes, D., Novak, M., et al. (2015). Genome-wide patterns of selection in 230 ancient Eurasians. Nature *528*, 499–503.

S4. Feldman, M., Fernández-Domínguez, E., Reynolds, L., Baird, D., Pearson, J., Hershkovitz, I., May, H., Goring-Morris, N., Benz, M., Gresky, J., et al. (2019). Late Pleistocene human genome suggests a local origin for the first farmers of central Anatolia. Nat. Commun. *10*, 1218.

S5. Yu, H., Spyrou, M.A., Karapetian, M., Shnaider, S., Radzevičiūtė, R., Nägele, K., Neumann, G.U., Penske, S., Zech, J., Lucas, M., et al. (2020). Paleolithic to Bronze Age Siberians Reveal Connections with First Americans and across Eurasia. Cell.

S6. Jones, E.R., Gonzalez-Fortes, G., Connell, S., Siska, V., Eriksson, A., Martiniano, R., McLaughlin, R.L., Gallego Llorente, M., Cassidy, L.M., Gamba, C., et al. (2015). Upper Palaeolithic genomes reveal deep roots of modern Eurasians. Nat. Commun. *6*, 8912.

S7. Lazaridis, I., Belfer-Cohen, A., Mallick, S., Patterson, N., Cheronet, O., Rohland, N., Bar-Oz, G., Bar-Yosef, O., Jakeli, N., Kvavadze, E., et al. (2018). Paleolithic DNA from the Caucasus reveals core of West Eurasian ancestry. bioRxiv, 423079.

S8. Yang, M.A., Gao, X., Theunert, C., Tong, H., Aximu-Petri, A., Nickel, B., Slatkin, M., Meyer, M., Pääbo, S., Kelso, J., et al. (2017). 40,000-Year-Old Individual from Asia Provides Insight into Early Population Structure in Eurasia. Curr. Biol. *27*, 3202–3208.e9.

S9. Fu, Q., Posth, C., Hajdinjak, M., Petr, M., Mallick, S., Fernandes, D., Furtwängler, A., Haak, W., Meyer, M., Mittnik, A., et al. (2016). The genetic history of Ice Age Europe. Nature *534*, 200–205.

S10. Olalde, I., Allentoft, M.E., Sánchez-Quinto, F., Santpere, G., Chiang, C.W.K., DeGiorgio, M., Prado-Martinez, J., Rodríguez, J.A., Rasmussen, S., Quilez, J., et al. (2014). Derived immune and ancestral pigmentation alleles in a 7,000-year-old Mesolithic European. Nature *507*, 225–228.

S11. Narasimhan, V.M., Patterson, N., Moorjani, P., Rohland, N., Bernardos, R., Mallick, S., Lazaridis, I., Nakatsuka, N., Olalde, I., Lipson, M., et al. (2019). The formation of human populations in South and Central Asia. Science *365*.

S12. Lazaridis, I., Nadel, D., Rollefson, G., Merrett, D.C., Rohland, N., Mallick, S., Fernandes, D., Novak, M., Gamarra, B., Sirak, K., et al. (2016). Genomic insights into the origin of farming in the ancient Near East. Nature *536*, 419–424.

S13. van de Loosdrecht, M., Bouzouggar, A., Humphrey, L., Posth, C., Barton, N., Aximu-Petri, A., Nickel, B., Nagel, S., Talbi, E.H., El Hajraoui, M.A., et al. (2018). Pleistocene North African genomes link Near Eastern and sub-Saharan African human populations. Science *360*, 548.

S14. Fu, Q., Hajdinjak, M., Moldovan, O.T., Constantin, S., Mallick, S., Skoglund, P., Patterson, N., Rohland, N., Lazaridis, I., Nickel, B., et al. (2015). An early modern human from Romania with a recent Neanderthal ancestor. Nature *524*, 216–219.

S15. Fu, Q., Li, H., Moorjani, P., Jay, F., Slepchenko, S.M., Bondarev, A.A., Johnson, P.L.F., Aximu-Petri, A., Prüfer, K., de Filippo, C., et al. (2014). Genome sequence of a 45,000-year-old modern human from western Siberia. Nature *514*, 445–449.

S16. Raghavan, M., Skoglund, P., Graf, K.E., Metspalu, M., Albrechtsen, A., Moltke, I., Rasmussen, S., Stafford, T.W., Jr, Orlando, L., Metspalu, E., et al. (2014). Upper Palaeolithic Siberian genome reveals dual ancestry of Native Americans. Nature *505*, 87–91.

S17. Seguin-Orlando, A., Korneliussen, T.S., Sikora, M., Malaspinas, A.-S., Manica, A., Moltke, I., Albrechtsen, A., Ko, A., Margaryan, A., Moiseyev, V., et al. (2014). Paleogenomics. Genomic structure in Europeans dating back at least 36,200 years. Science *346*, 1113–1118.

S18. Sikora, M., Seguin-Orlando, A., Sousa, V.C., Albrechtsen, A., Korneliussen, T., Ko, A., Rasmussen, S., Dupanloup, I., Nigst, P.R., Bosch, M.D., et al. (2017). Ancient genomes show social and reproductive behavior of early Upper Paleolithic foragers. Science *358*, 659–662.

S19. Gamba, C., Jones, E.R., Teasdale, M.D., McLaughlin, R.L., Gonzalez-Fortes, G., Mattiangeli, V., Domboróczki, L., Kővári, I., Pap, I., Anders, A., et al. (2014). Genome flux and stasis in a five millennium transect of European prehistory. Nat. Commun. *5*, 5257.

S20. Hajdinjak, M., Mafessoni, F., Skov, L., Vernot, B., Hübner, A., Fu, Q., Essel, E., Nagel, S., Nickel, B., Richter, J., et al. (2021). Initial Upper Palaeolithic humans in Europe had recent Neanderthal ancestry. Nature *592*, 253–257.

S21. Prüfer, K., Posth, C., Yu, H., Stoessel, A., Spyrou, M.A., Deviese, T., Mattonai, M., Ribechini, E., Higham, T., Velemínský, P., et al. (2021). A genome sequence from a modern human skull over 45,000 years old from Zlatý kůň in Czechia. Nat Ecol Evol.

S22. Posth, C., Renaud, G., Mittnik, A., Drucker, D.G., Rougier, H., Cupillard, C., Valentin, F., Thevenet, C., Furtwängler, A., Wißing, C., et al. (2016). Pleistocene Mitochondrial Genomes Suggest a Single Major Dispersal of Non-Africans and a Late Glacial Population Turnover in Europe. Curr. Biol. *26*, 827–833.

S23. Bollongino, R., Nehlich, O., Richards, M.P., Orschiedt, J., Thomas, M.G., Sell, C., Fajkosová, Z., Powell, A., and Burger, J. (2013). 2000 years of parallel societies in Stone Age Central Europe. Science *342*, 479–481.

S24. Fu, Q., Mittnik, A., Johnson, P.L.F., Bos, K., Lari, M., Bollongino, R., Sun, C., Giemsch, L., Schmitz, R., Burger, J., et al. (2013). A revised timescale for human evolution based on ancient mitochondrial genomes. Curr. Biol. *23*, 553–559.

S25. Fu, Q., Meyer, M., Gao, X., Stenzel, U., Burbano, H.A., Kelso, J., and Pääbo, S. (2013). DNA analysis of an early modern human from Tianyuan Cave, China. Proc. Natl. Acad. Sci. U. S. A. *110*, 2223–2227.

S26. Benazzi, S., Slon, V., Talamo, S., Negrino, F., Peresani, M., Bailey, S.E., Sawyer, S., Panetta, D., Vicino, G., Starnini, E., et al. (2015). Archaeology. The makers of the Protoaurignacian and implications for Neandertal extinction. Science *348*, 793–796.

S27. Ermini, L., Olivieri, C., Rizzi, E., Corti, G., Bonnal, R., Soares, P., Luciani, S., Marota, I., De Bellis, G., Richards, M.B., et al. (2008). Complete mitochondrial genome sequence of the Tyrolean Iceman. Curr. Biol. *18*, 1687–1693.

S28. Gilbert, M.T.P., Jenkins, D.L., Götherstrom, A., Naveran, N., Sanchez, J.J., Hofreiter, M., Thomsen, P.F., Binladen, J., Higham, T.F.G., Yohe, R.M., 2nd, et al. (2008). DNA from pre-Clovis human coprolites in Oregon, North America. Science *320*, 786–789.

S29. Günther, T., Malmström, H., Svensson, E.M., Omrak, A., Sánchez-Quinto, F., Kılınç, G.M., Krzewińska, M., Eriksson, G., Fraser, M., Edlund, H., et al. (2018). Population genomics of Mesolithic Scandinavia: Investigating early postglacial migration routes and high-latitude adaptation. PLoS Biol. *16*, e2003703.

S30. Vai, S., Sarno, S., Lari, M., Luiselli, D., Manzi, G., Gallinaro, M., Mataich, S., Hübner, A., Modi, A., Pilli, E., et al. (2019). Ancestral mitochondrial N lineage from the Neolithic “green” Sahara. Sci. Rep. *9*, 3530.

S31. Hublin, J.-J., Sirakov, N., Aldeias, V., Bailey, S., Bard, E., Delvigne, V., Endarova, E., Fagault, Y., Fewlass, H., Hajdinjak, M., et al. (2020). Initial Upper Palaeolithic Homo sapiens from Bacho Kiro Cave, Bulgaria. Nature, 1–4.

S32. Soares, P., Alshamali, F., Pereira, J.B., Fernandes, V., Silva, N.M., Afonso, C., Costa, M.D., Musilová, E., Macaulay, V., Richards, M.B., et al. (2012). The Expansion of mtDNA Haplogroup L3 within and out of Africa. Mol. Biol. Evol. *29*, 915–927.

S33. Gonder, M.K., Mortensen, H.M., Reed, F.A., de Sousa, A., and Tishkoff, S.A. (2007). Whole-mtDNA genome sequence analysis of ancient African lineages. Mol. Biol. Evol. *24*, 757–768.

S34. Torroni, A., Achilli, A., Macaulay, V., Richards, M., and Bandelt, H.-J. (2006). Harvesting the fruit of the human mtDNA tree. Trends Genet. *22*, 339–345.

S35. Behar, D.M., Villems, R., Soodyall, H., Blue-Smith, J., Pereira, L., Metspalu, E., Scozzari,

R., Makkan, H., Tzur, S., Comas, D., et al. (2008). The dawn of human matrilineal diversity. Am. J. Hum. Genet. *82*, 1130–1140.

S36. Barbieri, E., and Sestili, P. (2012). Reactive oxygen species in skeletal muscle signaling. J. Signal Transduct. *2012*, 982794.

S37. Behar, D.M., van Oven, M., Rosset, S., Metspalu, M., Loogväli, E.-L., Silva, N.M., Kivisild, T., Torroni, A., and Villems, R. (2012). A "Copernican" reassessment of the human mitochondrial DNA tree from its root. Am. J. Hum. Genet. *90*, 675–684.

S38. Derenko, M., Malyarchuk, B., Bahmanimehr, A., Denisova, G., Perkova, M., Farjadian, S., and Yepiskoposyan, L. (2013). Complete mitochondrial DNA diversity in Iranians. PLoS One *8*, e80673.

S39. Harich, N., Costa, M.D., Fernandes, V., Kandil, M., Pereira, J.B., Silva, N.M., and Pereira, L. (2010). The trans-Saharan slave trade - clues from interpolation analyses and high-resolution characterization of mitochondrial DNA lineages. BMC Evol. Biol. *10*, 138.

S40. Cerný, V., Fernandes, V., Costa, M.D., Hájek, M., Mulligan, C.J., and Pereira, L. (2009). Migration of Chadic speaking pastoralists within Africa based on population structure of Chad Basin and phylogeography of mitochondrial L3f haplogroup. BMC Evol. Biol. *9*, 63.

S41. Batini, C., Lopes, J., Behar, D.M., Calafell, F., Jorde, L.B., van der Veen, L., Quintana-Murci, L., Spedini, G., Destro-Bisol, G., and Comas, D. (2011). Insights into the demographic history of African Pygmies from complete mitochondrial genomes. Mol. Biol. Evol. *28*, 1099–1110.

S42. Ingman, M., Kaessmann, H., Pääbo, S., and Gyllensten, U. (2000). Mitochondrial genome variation and the origin of modern humans. Nature *408*, 708–713.

S43. Podgorná, E., Soares, P., Pereira, L., and Cerný, V. (2013). The genetic impact of the lake chad basin population in North Africa as documented by mitochondrial diversity and internal variation of the L3e5 haplogroup. Ann. Hum. Genet. *77*, 513–523.

S44. Vyas, S., Zaganjor, E., and Haigis, M.C. (2016). Mitochondria and Cancer. Cell *166*, 555–566.

S45. Pennarun, E., Kivisild, T., Metspalu, E., Metspalu, M., Reisberg, T., Moisan, J.-P., Behar, D.M., Jones, S.C., and Villems, R. (2012). Divorcing the Late Upper Palaeolithic demographic histories of mtDNA haplogroups M1 and U6 in Africa. BMC Evol. Biol. *12*, 234.

S46. Olivieri, A., Achilli, A., Pala, M., Battaglia, V., Fornarino, S., Al-Zahery, N., Scozzari, R., Cruciani, F., Behar, D.M., Dugoujon, J.-M., et al. (2006). The mtDNA legacy of the Levantine early Upper Palaeolithic in Africa. Science *314*, 1767–1770.

S47. González, A.M., Larruga, J.M., Abu-Amero, K.K., Shi, Y., Pestano, J., and Cabrera, V.M. (2007). Mitochondrial lineage M1 traces an early human backflow to Africa. BMC Genomics *8*, 223.

S48. Fernandes, V., Alshamali, F., Alves, M., Costa, M.D., Pereira, J.B., Silva, N.M., Cherni, L., Harich, N., Cerny, V., Soares, P., et al. (2012). The Arabian cradle: mitochondrial relicts of the first steps along the southern route out of Africa. Am. J. Hum. Genet. *90*, 347–355.

S49. Olivieri, A., Pala, M., Gandini, F., Hooshiar Kashani, B., Perego, U.A., Woodward, S.R., Grugni, V., Battaglia, V., Semino, O., Achilli, A., et al. (2013). Mitogenomes from two uncommon haplogroups mark late glacial/postglacial expansions from the near east and neolithic dispersals within Europe. PLoS One *8*, e70492.

S50. Greenspan, B. (2008). Family Tree DNA.

S51. Janssen, A.J.M., Trijbels, F.J.M., Sengers, R.C.A., Wintjes, L.T.M., Ruitenbeek, W., Smeitink, J.A.M., Morava, E., van Engelen, B.G.M., van den Heuvel, L.P., and Rodenburg, R.J.T. (2006). Measurement of the energy-generating capacity of human muscle mitochondria: diagnostic procedure and application to human pathology. Clin. Chem. *52*, 860–871.

S52. Schönberg, A., Theunert, C., Li, M., Stoneking, M., and Nasidze, I. (2011). High-throughput sequencing of complete human mtDNA genomes from the Caucasus and West Asia: high diversity and demographic inferences. Eur. J. Hum. Genet. *19*, 988–994.

S53. Kushniarevich, A., Sivitskaya, L., Danilenko, N., Novogrodskii, T., Tsybovsky, I., Kiseleva, A., Kotova, S., Chaubey, G., Metspalu, E., Sahakyan, H., et al. (2013). Uniparental genetic heritage of belarusians: encounter of rare middle eastern matrilineages with a central European mitochondrial DNA pool. PLoS One *8*, e66499.

S54. Costa, M.D., Pereira, J.B., Pala, M., Fernandes, V., Olivieri, A., Achilli, A., Perego, U.A., Rychkov, S., Naumova, O., Hatina, J., et al. (2013). A substantial prehistoric European ancestry amongst Ashkenazi maternal lineages. Nat. Commun. *4*, 1–10.

S55. Derbeneva, O.A., Starikovskaya, E.B., Wallace, D.C., and Sukernik, R.I. (2002). Traces of early Eurasians in the Mansi of northwest Siberia revealed by mitochondrial DNA analysis. Am. J. Hum. Genet. *70*, 1009–1014.

S56. Shlush, L.I., Atzmon, G., Weisshof, R., Behar, D., Yudkovsky, G., Barzilai, N., and Skorecki, K. (2008). Ashkenazi Jewish centenarians do not demonstrate enrichment in mitochondrial haplogroup J. PLoS One *3*, e3425.

S57. Kujanová, M., Pereira, L., Fernandes, V., Pereira, J.B., and Cerný, V. (2009). Near eastern neolithic genetic input in a small oasis of the Egyptian Western Desert. Am. J. Phys. Anthropol. *140*, 336–346.

S58. Costa, M.D., Cherni, L., Fernandes, V., Freitas, F., Ammar El Gaaied, A.B., and Pereira, L. (2009). Data from complete mtDNA sequencing of Tunisian centenarians: testing haplogroup association and the “golden mean” to longevity. Mech. Ageing Dev. *130*, 222–226.

S59. Fraumene, C., Belle, E.M.S., Castrì, L., Sanna, S., Mancosu, G., Cosso, M., Marras, F., Barbujani, G., Pirastu, M., and Angius, A. (2006). High resolution analysis and phylogenetic

network construction using complete mtDNA sequences in sardinian genetic isolates. Mol. Biol. Evol. *23*, 2101–2111.

S60. Raule, N., Sevini, F., Li, S., Barbieri, A., Tallaro, F., Lomartire, L., Vianello, D., Montesanto, A., Moilanen, J.S., Bezrukov, V., et al. (2014). The co-occurrence of mtDNA mutations on different oxidative phosphorylation subunits, not detected by haplogroup analysis, affects human longevity and is population specific. Aging Cell *13*, 401–407.

S61. Achilli, A., Olivieri, A., Pala, M., Hooshiar Kashani, B., Carossa, V., Perego, U.A., Gandini, F., Santoro, A., Battaglia, V., Grugni, V., et al. (2011). Mitochondrial DNA backgrounds might modulate diabetes complications rather than T2DM as a whole. PLoS One *6*, e21029.

S62. Pereira, L., Gonçalves, J., Franco-Duarte, R., Silva, J., Rocha, T., Arnold, C., Richards, M., and Macaulay, V. (2007). No evidence for an mtDNA role in sperm motility: data from complete sequencing of asthenozoospermic males. Mol. Biol. Evol. *24*, 868–874.

S63. Thalmann, O., Shapiro, B., Cui, P., Schuenemann, V.J., Sawyer, S.K., Greenfield, D.L., Germonpré, M.B., Sablin, M.V., López-Giráldez, F., Domingo-Roura, X., et al. (2013). Complete mitochondrial genomes of ancient canids suggest a European origin of domestic dogs. Science *342*, 871–874.

S64. Loog, L., Thalmann, O., Sinding, M.-H.S., Schuenemann, V.J., Perri, A., Germonpré, M., Bocherens, H., Witt, K.E., Samaniego Castruita, J.A., Velasco, M.S., et al. (2020). Ancient DNA suggests modern wolves trace their origin to a Late Pleistocene expansion from Beringia. Mol. Ecol. *29*, 1596–1610.

S65. Skoglund, P., Ersmark, E., Palkopoulou, E., and Dalén, L. (2015). Ancient wolf genome reveals an early divergence of domestic dog ancestors and admixture into high-latitude breeds. Curr. Biol. *25*, 1515–1519.

S66. Matsumura, S., Inoshima, Y., and Ishiguro, N. (2014). Reconstructing the colonization history of lost wolf lineages by the analysis of the mitochondrial genome. Mol. Phylogenet. Evol. *80*, 105–112.

S67. Björnerfeldt, S., Webster, M.T., and Vilà, C. (2006). Relaxation of selective constraint on dog mitochondrial DNA following domestication. Genome Res. *16*, 990–994.

S68. Chen, L., and Zhang, H.H. (2009). Canis lupus chanco mitochondrion, complete genome.

S69. Zhang, H., Zhang, J., Chen, L., and Liu, G. (2014). The complete mitochondrial genome of Chinese Shinjang wolf. Mitochondrial DNA *25*, 106–108.

S70. Pang, J.-F., Kluetsch, C., Zou, X.-J., Zhang, A.-B., Luo, L.-Y., Angleby, H., Ardalan, A., Ekström, C., Sköllermo, A., Lundeberg, J., et al. (2009). mtDNA data indicate a single origin for dogs south of Yangtze River, less than 16,300 years ago, from numerous wolves. Mol. Biol. Evol. *26*, 2849–2864.

S71. Arnason, U., Gullberg, A., Janke, A., and Kullberg, M. (2007). Mitogenomic analyses of caniform relationships. Mol. Phylogenet. Evol. *45*, 863–874.

S72. Arnason, U., Gullberg, A., Janke, A., and Kullberg, M. (2007). Mitogenomic analyses of caniform relationships. Mol. Phylogenet. Evol. *45*, 863–874.

S73. Gopalakrishnan, S., Sinding, M.-H.S., Ramos-Madrigal, J., Niemann, J., Samaniego Castruita, J.A., Vieira, F.G., Carøe, C., Montero, M. de M., Kuderna, L., Serres, A., et al. (2018). Interspecific Gene Flow Shaped the Evolution of the Genus Canis. Curr. Biol. *28*, 3441–3449.e5.

S74. Hassanin, A., Delsuc, F., Ropiquet, A., Hammer, C., Jansen van Vuuren, B., Matthee, C., Ruiz-Garcia, M., Catzeflis, F., Areskoug, V., Nguyen, T.T., et al. (2012). Pattern and timing of diversification of Cetartiodactyla (Mammalia, Laurasiatheria), as revealed by a comprehensive analysis of mitochondrial genomes. C. R. Biol. *335*, 32–50.

S75. Douglas, K.C., Halbert, N.D., Kolenda, C., Childers, C., Hunter, D.L., and Derr, J.N. (2011). Complete mitochondrial DNA sequence analysis of Bison bison and bison-cattle hybrids: function and phylogeny. Mitochondrion *11*, 166–175.

S76. Zeyland, J., Wolko, L., Bocianowski, J., Szalata, M., Słomski, R., Dzieduszycki, A.M., Ryba, M., Przystałowska, H., and Lipiński, D. (2013). Complete mitochondrial genome of wild aurochs (Bos primigenius) reconstructed from ancient DNA. Pol. J. Vet. Sci. *16*, 265–273.

S77. Zhang, H., Paijmans, J.L.A., Chang, F., Wu, X., Chen, G., Lei, C., Yang, X., Wei, Z., Bradley, D.G., Orlando, L., et al. (2013). Morphological and genetic evidence for early Holocene cattle management in northeastern China. Nat. Commun. *4*, 2755.

S78. Huang, C., Zhang, Q., Wu, X., Dawa, Y., Fu, D., Jiang, H., Chu, M., Dingkao, R., Guo, X., Basang, W., et al. (2019). The complete mitochondrial genome sequence and phylogenetic analysis of Niangya yak (Bos grunniens). Mitochondrial DNA B Resour *5*, 202–203.

S79. Chunnian, L., Wu, X., Ding, X., Wang, H., Guo, X., Chu, M., Bao, P., and Yan, P. (2016). Characterization of the complete mitochondrial genome sequence of wild yak (Bos mutus). Mitochondrial DNA A DNA Mapp Seq Anal *27*, 4266–4267.

S80. Kirillova, I.V., Zanina, O.G., Chernova, O.F., Lapteva, E.G., Trofimova, S.S., Lebedev, V.S., Tiunov, A.V., Soares, A.E.R., Shidlovskiy, F.K., and Shapiro, B. (2015). An ancient bison from the mouth of the Rauchua River (Chukotka, Russia). Quat. Res. *84*, 232–245.

S81. Węcek, K., Hartmann, S., Paijmans, J.L.A., Taron, U., Xenikoudakis, G., Cahill, J.A., Heintzman, P.D., Shapiro, B., Baryshnikov, G., Bunevich, A.N., et al. (2016). Complex Admixture Preceded and Followed the Extinction of Wisent in the Wild. Mol. Biol. Evol. *34*, 598–612.

S82. Soubrier, J., Gower, G., Chen, K., Richards, S.M., Llamas, B., Mitchell, K.J., Ho, S.Y.W., Kosintsev, P., Lee, M.S.Y., Baryshnikov, G., et al. (2016). Early cave art and ancient DNA record the origin of European bison. Nat. Commun. *7*, 13158.

S83. Massilani, D., Guimaraes, S., Brugal, J.-P., Bennett, E.A., Tokarska, M., Arbogast, R.-M., Baryshnikov, G., Boeskorov, G., Castel, J.-C., Davydov, S., et al. (2016). Past climate changes, population dynamics and the origin of Bison in Europe. BMC Biol. *14*, 93.

S84. Miretti, M.M., Pereira, H.A., Jr, Poli, M.A., Contel, E.P.B., and Ferro, J.A. (2002). African-derived mitochondria in South American native cattle breeds (Bos taurus): evidence of a new taurine mitochondrial lineage. J. Hered. *93*, 323–330.

S85. Qian, J.X., Dong, K.J., Huang, Y.J., Yang, B.Z., He, M., Liu, Z.J., and Li, J. (2004). Bubalus carabanensis mitochondrion, complete genome.

S86. San, A., Wang, W.A., and Yu, L. (2007). Bos grunniens mitochondrion, complete genome.

S87. Chung, H.Y., and Ha, J.M. (2005). Bos taurus mitochondrion, complete genome.

S88. Achilli, A., Olivieri, A., Pellecchia, M., Uboldi, C., Colli, L., Al-Zahery, N., Accetturo, M., Pala, M., Hooshiar Kashani, B., Perego, U.A., et al. (2008). Mitochondrial genomes of extinct aurochs survive in domestic cattle. Curr. Biol. *18*, R157–8.

S89. Lipinski, D., Zeyland, J., Kowalska, M., Szalata, M., Dzieduszycki, A.M., Ryba, M.S., and Slomski, R. (2009). Bos javanicus mitochondrion, complete genome.

S90. Edwards, C.J., Magee, D.A., Park, S.D.E., McGettigan, P.A., Lohan, A.J., Murphy, A., Finlay, E.K., Shapiro, B., Chamberlain, A.T., Richards, M.B., et al. (2010). A complete mitochondrial genome sequence from a mesolithic wild aurochs (Bos primigenius). PLoS One *5*, e9255.

S91. Zeyland, J., Wolko, L., Lipiński, D., Woźniak, A., Nowak, A., Szalata, M., Bocianowski, J., and Słomski, R. (2012). Tracking of wisent-bison-yak mitochondrial evolution. J. Appl. Genet. *53*, 317–322.

S92. Na, R.-S., Zhao, Y.-J., Gao, H.-J., An, T.-W., Huang, Y.-F., and E, G.-X. (2016). Complete mitochondrial genome of the Yakow (Bos primigenius taurus × Bos grunniens) in China. Mitochondrial DNA A DNA Mapp Seq Anal *27*, 3826–3827.

S93. Marsolier-Kergoat, M.-C., Palacio, P., Berthonaud, V., Maksud, F., Stafford, T., Bégouën, R., and Elalouf, J.-M. (2015). Hunting the Extinct Steppe Bison (Bison priscus) Mitochondrial Genome in the Trois-Frères Paleolithic Painted Cave. PLoS One *10*, e0128267.

S94. Derr, J.N., Halbert, N.D., Gogan, P., and Douglas, K.C. (2016). Bison bonasus mitochondrion, complete genome.
